# Environment Rearrangement Slows Down the Tunneling Rates of Proton Transfer in Sandwich-Like Molecular Clusters

**DOI:** 10.1021/acs.jpclett.6c01033

**Published:** 2026-07-30

**Authors:** Jingling Hong, Ziye Qi, Yao Wang, Denis S. Tikhonov, Wei Fang, Xiao Zheng, Mingfei Zhou, Weixing Li

**Affiliations:** † Department of Chemistry, State Key Laboratory of Porous Materials for Separation and Conversion, Shanghai Key Laboratory of Molecular Catalysis and Innovative Materials, 12478 Fudan University, Songhu Rd. 2005, 200438 Shanghai, China; ‡ Hefei National Research Center for Physical Sciences at the Microscale, 12652 University of Science and Technology of China, Hefei, Anhui 230026, China; ¶ Center for Free-Electron Laser Science CFEL, 28332 Deutsches Elektronen-Synchrotron DESY, Notkestr. 85, 22607 Hamburg, Germany; § Department of Physics, University of Hamburg, 22607 Hamburg, Germany

## Abstract

We present the measurement of tunneling splitting for double proton transfer in a propiolic acid-formic acid dimer embedded within sandwich-like molecular clusters with fluorobenzene and helium, utilizing rotational spectroscopy. We systematically compare our new experimental results with previously measured experimental tunneling splittings for similar carboxylic acid molecular clusters, namely a propiolic acid-formic acid dimer and a formic acid dimer with and without fluorobenzene. The data reveal a consistent trend: each added molecular layer further suppresses the tunneling rate, with the ternary FA-PA@PhF cluster exhibiting a dramatically reduced splitting (50.89 MHz) relative to the bare dimer (291.43 MHz), and an additional decrease upon helium tagging (47.58 MHz). The comparison is also supported with theory, using ring-polymer instanton theory and by solving effective one-dimensional Schrödinger equation models. The observed trend of decreasing tunneling rates with increasing environment complexity suggests that the environment acts as a penalty on proton transfer, as explained by the Marcus-type environment reorganization model. These findings provide not only the most complex molecular system to date with spectroscopically resolved proton tunneling, but also a general conceptual framework for understanding and potentially engineering quantum proton dynamics in tailored molecular environments.

## Graphical abstract

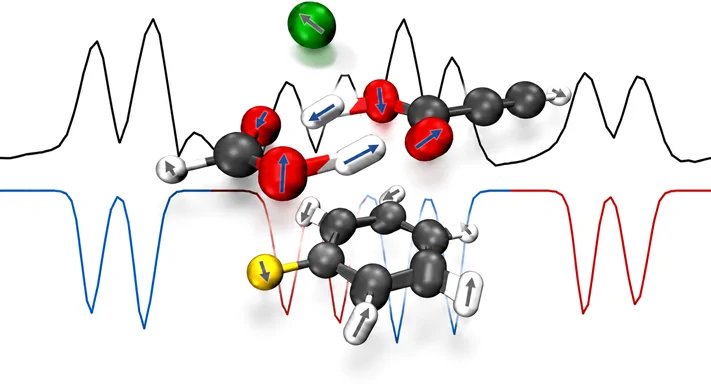

Proton transfer through hydrogen bonds via the Grotthuss mechanism (A – H···B ⇆ A···H – B)^1,2^ represents a fundamental chemical reaction happening in a double-well potential. Despite energy barriers often exceeding the thermal energy (tens or even hundreds of kJ/mol *vs RT* = 2.3 kJ/mol at the room temperature),^3^ protons can traverse these barriers via quantum tunneling, due to their low mass.^4–11^ The tunneling is most pronounced in symmetric double-well potentials, enabling measurable coherent tunneling rates even for high barriers.^12^ In a symmetric double-well potential, as shown in Figure 1, the doubly degenerate vibrational level splits into two sublevels of opposite parity, one symmetric (0^+^) and the other antisymmetric (0^–^) with energies $E_{0^{\pm }}$ , respectively. The energy difference between the two substates $\Delta E=E_{0^{-}}-E_{0^{+}}$ is termed tunneling splitting, and it corresponds to the oscillation frequency of the molecule shuttling between the wells with the respective period τ = *h*/Δ*E*.^13^ Such oscillations are experimentally measurable.^14^

**Figure 1:**
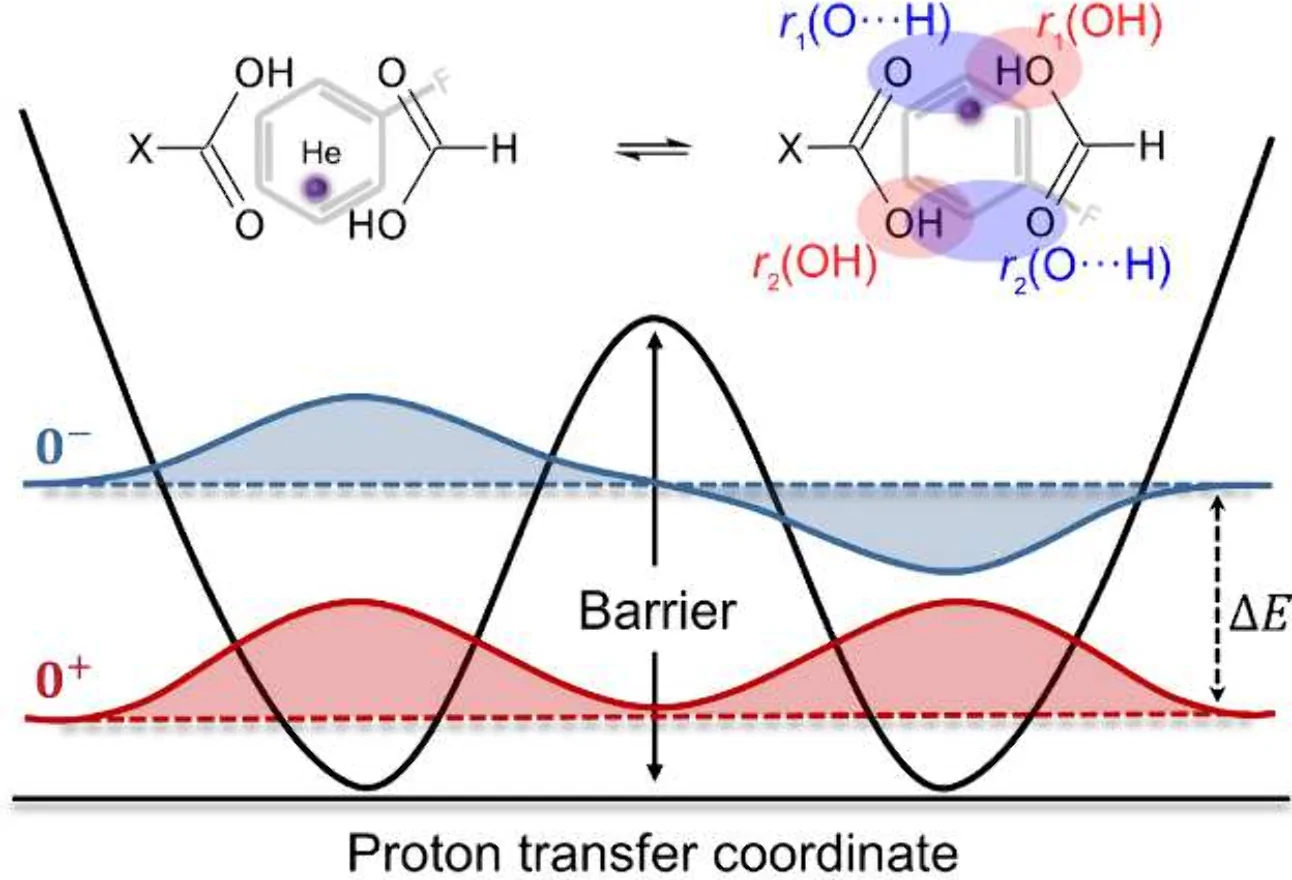
A schematic potential energy surface and split ground-state energy levels for the double proton transfer within a carboxylic acid dimer embedded in a sandwich-like molecular complex. The oxygen–hydrogen distances (*r*(OH)) that map the proton transfer motion are also shown in the structure on the right.

This physical meaning can be easily extracted from the semiclassical Gamow approximation to the tunneling splitting,^15,16^ in which the splitting energy Δ*E* is given as

1 $$\Delta E=\frac{h\nu_{0}}{\pi }\;\exp\left(-\frac{S}{\hbar }\right)$$

where ν_0_ is the vibrational frequency of the motion within a given potential well, *h* and *ℏ* are normal and reduced Planck constants, and *S* is the absolute value of the imaginary action for the underbarrier motion. In the case of a rectangular potential, this action can be expressed as $S=\sqrt{2m\cdot BH}\cdot L$ , where *m* is the mass of the moving particle, BH is the barrier height for the tunneling to surpass, and *L* is the distance the tunneling particle has to travel to cross the barrier. The tunneling frequency between potential wells ν = Δ*E*/*h* is thus proportional to the frequency of the particles hitting the barrier (ν_0_), multiplied by the probability of tunneling through the barrier (exp­(−*S*/*ℏ*)).

One of the systems exhibiting the proton transfer via the Grotthus mechanism are the carboxylic acid dimers. They exist as eight-membered rings formed via two hydrogen bonds and can serve as prototypes for studying concerted double proton transfer in adenine-thymine (AT) base pairs, having been extensively investigated through both experimental and theoretical approaches.^17–24^ While proton tunneling in isolated donor–acceptor systems has been extensively studied,^25–27^ environmental effects on tunneling dynamics remain elusive, despite their critical role in open quantum systems spanning biomolecular catalysts to quantum sensors.^28–30^ The influence of the environment on the tunneling dynamics was extensively studied theoretically over the years.^31–33^ As for the experiment, several studies previously observed the environmental influence of the proton transfer in porphyrins and water clusters on various surfaces.^34–36^ In the only existing gas-phase study of environmental influence on proton tunneling systems, we showed that the tunneling splitting of the formic acid dimer decreased upon clustering with fluorobenzene, which we attributed to the environment’s reorganization penalty.^37^ However, no systematic picture of the effects of different molecular environments on the proton transfer reaction is available due to the lack of comparable experimentally measured tunneling splittings for environment-modulated proton transfer reactions.

In this work, we systematically investigate the mechanisms that modulate proton transfer rate by coupling with surrounding atomic and molecular species. We do this by extending the gas-phase experimental data on carboxylic acid dimers by measuring the formic acid (FA)-propiolic acid (PA) dimer embedded within a “sandwich-like” molecular cluster with fluorobenzene (PhF). In addition to that, the helium-tagged version of this cluster was observed. In total, with the two new molecular species measured in this work, we took the proton transfer tunneling splittings from the literature data for pristine FA-PA dimer^17,18^ and the data on the free and PhF-bound FA-FA dimer (commonly abbreviated as FAD in previous studies).^37,38^ This produced a data set of five gas-phase molecular species with experimental tunneling splittings allowed for monitoring the environmental effects on the double hydrogen exchange reaction in carboxylic acid dimers. We employed three theoretical approaches to characterize the effects of external molecular influences on proton motions. We used the three *ab initio* methods: ring-polymer instanton theory (RPIT), effective one-dimensional Schrödinger equation (E1DSE) approach, and Bohmian methadynamics (BMTD) simulations. In addition to that, we applied the Marcus-type^29^ environment reorganization model (ERM) approach, introduced in ref 37, which we generalized to an arbitrary number of coupled environment motions. The theoretical comparison revealed a general mechanistic paradigm: environmental flexibility, rather than rigidity, slows down quantum tunneling by complicating the pathway of the motion, which is supported by excellent quantitative agreement with experimental measurements.

The molecular clusters were formed via supersonic expansion, which cools their rotational temperature to a few Kelvin and isolates them from collisions during measurement, enabling the formation and observation of low-energy conformers. A gas mixture containing FA, PA, and PhF was seeded into helium, the carrier gas, with a backing pressure of 10 bar. Each component was present at a concentration of approximately 0.5%. Then, the rotational spectra of the clusters were measured using the chirp-polarization Fourier transform microwave (CP-FTMW) spectrometer within 2–8 GHz (see Methods in the Supporting Information (SI) for details).^39,40^ In general, molecular monomers and smaller clusters, such as FA, PA, PhF, FA dimer (which has no permanent dipole moment), FA-PA, and FA-FA@PhF in this experiment,^17,18,37,41–43^ are the most abundant species in the molecular beam. After removing the spectral lines of known abundant species, a set of weak spectra with a characteristic doublet splitting pattern was observed (Figure 2A), which have been assigned to the FA-PA@PhF cluster. Coherent tunneling of particles in a double-well potential lifts the degeneracy of energy levels, resulting in the splitting of each rotational level into two sublevels (denoted as 0^+^ and 0^–^ for the vibrational ground state). In the spectra (Figure 2A), transitions associated with these sublevels are marked in red (0^+^) and blue (0^–^), respectively. The observed spectral splittings have been fitted into a two-state tunneling-rotation coupled Hamiltonian, as described in the SI. In addition to assigning the two-layer cluster FA-PA@PhF, we also detected another set of weaker signals in the spectra, which have been identified as the cluster FA-PA@PhF··· He (Figure 2D). The resulting spectroscopic parameters of the two species are summarized in Table 1.

**Figure 2:**
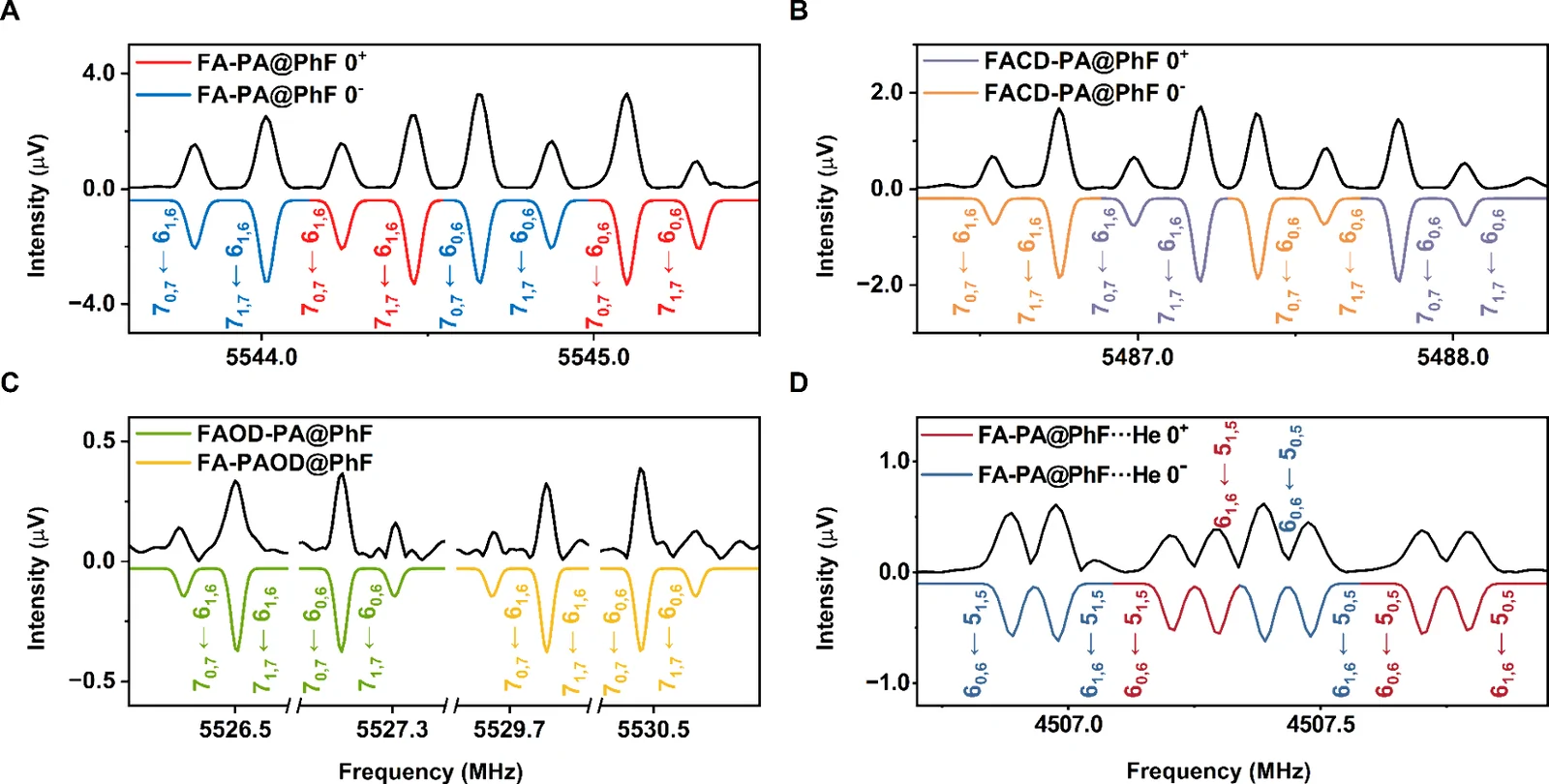
Sections of CP-FTMW spectra of different molecular complexes, including (A) FA-PA@PhF, (B) FACD-PA@PhF, (C) FAOD-PA@PhF and FA-PAOD@PhF, and (D) FA-PA@PhF··· He. The black trace represents the experimental spectra, while the colored spectra with negative intensity are the simulations derived from the fitted spectral parameters, calculated dipole moments, and a temperature of approximately 1 K. The quantum numbers follow the standard nomenclature for the rotational energy levels of an asymmetric top, denoted as $J_{K_{a}K_{c}}$ , where *J* represents the total rotational angular momentum quantum number, while *K*_*a*_ and *K*_*c*_ correspond to the projections of *J* onto the principal rotational axes (*a* and *c*) in the limiting cases of prolate and oblate symmetric tops, respectively.

**Table 1:** Spectroscopic Constants for FA-PA@PhF and FA-PA@PhF···He Complexes^a^

	Experiment^a^		Theory	
	Parent(0^+^)	Parent(0^–^)	Conformer #1	Conformer #2
FA-PA@PhF				
*A* [MHz]	712.4895(28)	712.4881(28)	721.8	789.1
*B* [MHz]	528.07048(65)	528.07143(57)	535.8	473.8
*C* [MHz]	382.1440(28)	382.1438(28)	386.3	363.3
*D*_*J*_ [kHz]	0.1127(10)			
*D*_*JK*_ [kHz]	0.2357(62)			
*d*_1_ [kHz]	–0.03593(61)			
Δ*E*_01_ [MHz]	50.8949(61)			
*F*_*bc*_ [MHz]	12.4488(13)			
*F*_*ca*_ [MHz]	18.191(26)			
Δ*E* [MHz]	50.8949(61)		68.82^e^	20.16^*f*^
μ_*a*_/μ_*b*_/μ_*c*_ [D]^b^	intra/intra-/inter-		1.8/1.2/0.2	0.3/0.5/0.1
σ [kHz]^c^	4.7			
*N* ^d^	203			
FA-PA@PhF···He				
*A* [MHz]	643.1463(68)	643.1496(70)	657.1	749.0
*B* [MHz]	522.4166(17)	522.4195(17)	531.3	447.1
*C* [MHz]	358.9677(69)	358.9678(69)	366.8	365.1
*D*_*J*_ [kHz]	0.1581(31)			
*D*_*K*_ [kHz]	0.465(22)			
*D*_*JK*_ [kHz]	–0.065(14)			
*d*_1_ [kHz]	–0.0572(16)			
*F*_*bc*_ [MHz]	9.2729(86)			
*F*_*ca*_ [MHz]	16.905(57)			
Δ*E* [MHz]	47.579(38)		59.74^e^	12.71^e^
μ_*a*_/μ_*b*_/μ_*c*_ [D]^b^	intra/intra-/inter-		1.8/1.2/0.2	0.3/0.4/0.0
σ [kHz]^c^	4.7			
*N* ^d^	229			

a *A*, *B*, and *C* represent the rotational constants; *D*_*J*_, *D*_*JK*_, *D*_*K*_, *d*_1_, and *d*_2_ denote the quartic centrifugal distortion constants; *F*_*bc*_ and *F*_*ca*_ signify the Coriolis coupling parameters in the two-state Hamiltonian; Δ*E* is the tunneling splitting between states 0^+^ and 0^–^.

b The experimental results were derived using the *I* ^*r*^ representation of Watson’s *S* reduction Hamiltonian, with standard deviations in parentheses expressed in units of the last two digits.

c Intra- and inter- denote intrastate (0^+^ ↔ 0^+^ or 0^–^↔ 0^–^) and interstate (0^+^ ↔ 0^–^) transitions between the tunneling substates, respectively. In this work, only the intrastate transitions (*a*- and *b*-type) were observed; the interstate transitions (*c*-type) were not detected due to the small magnitudes of their dipole moments.

d Root-mean-square deviation of the fit.

e Number of the lines in the fit.

f Theoretical values were obtained with RPIT model at the CCSD­(T)-F12-RI/cc-pVDZ-F12//B3LYP-D3­(BJ)/def2-TZV level of theory.

To identify the energetically preferred structures of the molecular clusters, a conformational search was conducted using the CREST software^44^ on a semiempirical GFN2-xTB^45^ potential energy surface (PES). Then the initial structures were subsequently optimized at the B3LYP-D3­(BJ)/def2-TZVPP level of theory,^46–48^ and their relative single point energies at these geometries were calculated at the DLPNO­(TightPNO)-CCSD­(T)/aug-cc-pVTZ level of theory.^49–51^ Among the resulting structures, the two most stable configurations of FA-PA@PhF (Figure 3A), designated as “conformer #1” and “conformer #2,” were identified. The computational analysis revealed that “conformer #1”, in which FA interacts with the polar C–F moiety and the PA lies on top of the benzene moiety, is energetically more favorable than “conformer #2”, where the FA and PA are swapped in places. For the FA-PA@PhF···He cluster, the most stable configuration features a helium atom positioned above the FA-PA dimer of conformer #1 of FA-PA@PhF, effectively sandwiching the carboxylic acid dimer between PhF and He. A comparable structure in which the helium atom interacts with conformer #2 of FA-PA@PhF was found to be 2.2 kJ/mol less stable (Figure 3B). Additionally, two configurations, with the helium atom positioned laterally to the FA-PA@PhF cluster, are optimized at energies 0.7 and 2.4 kJ/mol above the global minimum ().

**Figure 3:**
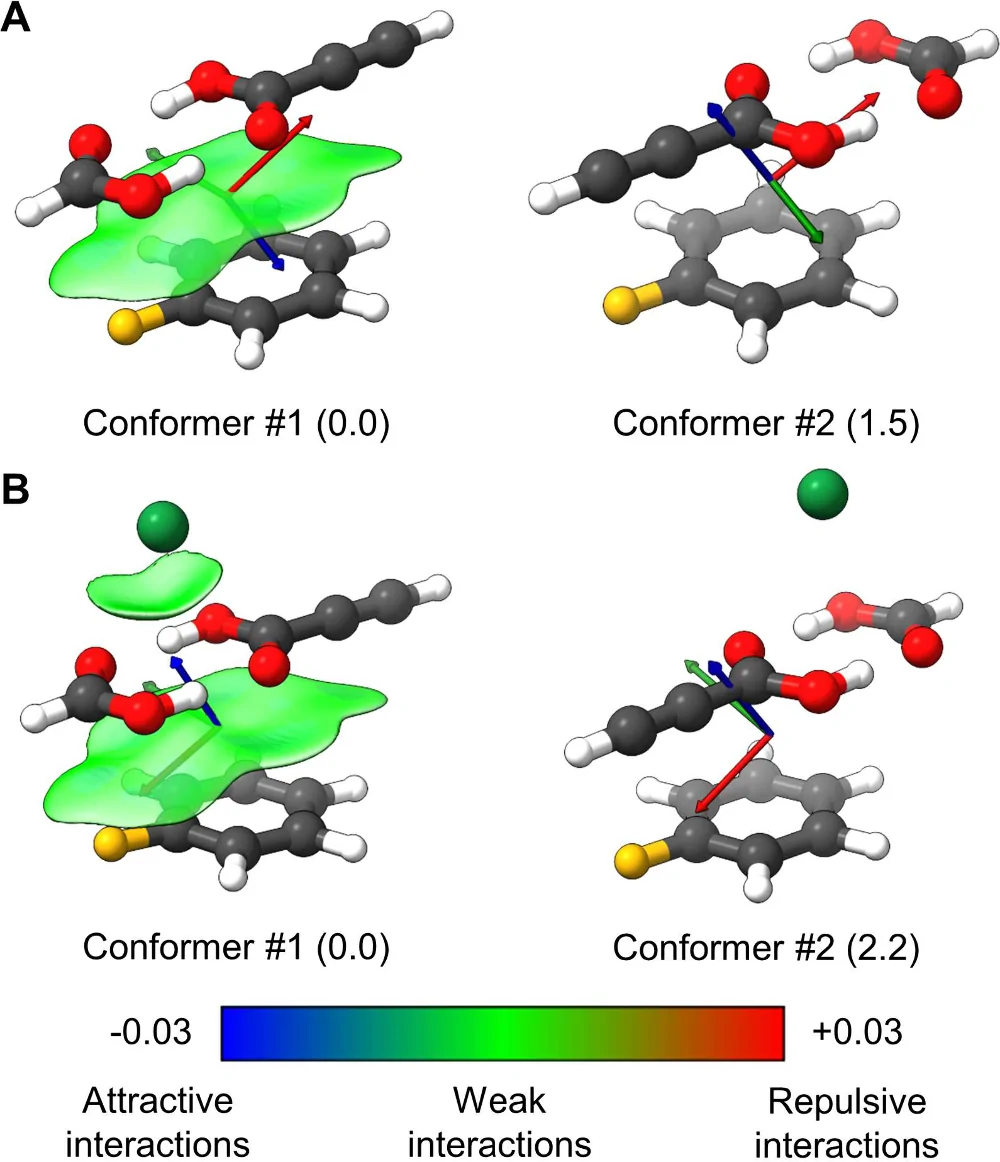
Two low-lying conformers of (A) FA-PA@PhF and (B) FA-PA@PhF···He, respectively. Relative energies (kJ/mol), calculated and corrected for ZPE and BSSE at the B3LYP-D3­(BJ)/def2-TZVPP level, are given in parentheses. The principal axes *a*, *b*, and *c* are depicted in red, blue, and green, respectively. The isosurfaces are derived from IGMH calculation (isovalue of δ_*g*_ = 0.0015 au; δ_*g*_ is a function of electron density gradient reflecting the interaction) and colored on a BGR scale corresponding to the value of sign­(λ_2_)­ρ, where ρ is the actual electron density and λ_2_ represents the second largest eigenvalue of the electron density Hessian matrix.

We employed the Independent Gradient Model based on Hirshfeld partition (IGMH) method^52^ to investigate the primary interlayer interactions in conformer #1 of FA-PA@PhF and FA-PA@PhF···He, treating FA-PA as a single moiety. As shown in Figure 3, the green isosurfaces (δ_*g*_ = 0.0015 au), colored by sign­(λ_2_)­ρ on a BGR scale (where λ_2_ is the second largest eigenvalue of the electron density Hessian matrix), reveal that the interlayer interactions are dominated by weak van der Waals forces, mainly due to π–π stacking. The scatter plots of sign­(λ_2_)­ρ versus δ_*g*_ were generated to further support the IGMH analysis, as presented in Figure S6 in the SI. To elucidate this conformational preference of FA-PA clustering with PhF moiety, we conducted two-body symmetry-adapted perturbation theory (SAPT) calculations^53^ for the two energetically preferred conformers of FA-PA@PhF and FA-PA@PhF···He, respectively. From these calculations (Figure S7 in the SI), we observed that for both FA-PA@PhF and FA-PA@PhF···He, the relative orientation of the FA-PA dimer with respect to PhF in conformer #1 enhances the electrostatic interaction between the fragments by 1.9 and 2.5 kJ/mol, respectively, compared to conformer #2. This effect likely arises from a favorable electrostatic attraction between the electronegative F and the positively charged C atom in FA. Additionally, the dispersion interaction in conformer #1 is approximately 1.4 and 2 kJ/mol stronger than in conformer #2, respectively, likely due to enhanced π–π interactions between the extended C≡C group of PA and the nonpolar phenyl group of PhF.

The experimental rotational constants (Table 1) indicate that the observed rotational spectra for FA-PA@PhF and FA-PA@PhF···He both correspond to conformer #1 rather than conformer #2 or any other predictions. The maximum deviation of rotational constants between the experiment and calculation of conformer #1 for FA-PA@PhF is about 1.5% and about 2.0% for FA-PA@PhF···He. To validate our assignment, we further measured the singly deuterated species FACD-PA@PhF, where the C–H hydrogen in FA is substituted by deuterium. This allowed us to determine the magnitudes of the substituted atom’s coordinates in the principal inertial coordinate system of the parent molecule using Kraitchman’s equations. This approach is referred to as the *r*_*s*_-structure.^56^ As illustrated in Figure S5 in the SI, the *r*_*s*_ coordinates of FA-PA@PhF match very well with the theoretically calculated result of conformer #1, which unambiguously confirms our assignment.

The tunneling splittings Δ*E* determined for FA-PA@PhF and FA-PA@PhF···He, as well as for previously determined FA-FA,^38^ FA-PA,^18^ and FA-FA@PhF^37^ species, are given in Table 2. To verify these experimental values, we have turned to the complementary theoretical methods used in literature for computing these tunneling splittings, namely, RPIT^57,58^ at the CCSD­(T)-F12-RI/cc-pVDZ-F12//B3LYP-D3­(BJ)/def2-TZVP^46–48,59,59–62^ level of theory and E1DSE^54,55^ approach with the DLPNO­(TightPNO)-CCSD­(T)/aug-cc-pVTZ//B3LYP-D3­(BJ)/def2-TZVPP^46–49,51,63^ approximation. Both of these methods used a well-benchmarked B3LYP exchange-correlation functional to compute the reaction pathway, as it provides good structures and vibrational frequencies, but lowers the proton transfer barriers.^64–67^ This drawback was corrected by single-point coupled cluster^59,60^ calculations on the reaction pathways. To characterize the performance of these theoretical methods, we calculated several statistical measures showing the correlation and deviation between experimental values and the theoretical predictions. To numerically depict the correlation, we used the Pearson correlation coefficient (PCC). The relative deviation between the theoretical and experimental values was computed in the form of the root-mean-square relative error (RMSRE):

2 $$RMSRE=\sqrt{\frac{1}{K}\overset{K}{\underset{k=1}{\sum }}{\left(\frac{\Delta E_{k}^{\left(\exp\right)}-\Delta E_{k}^{\left(theor\right)}}{\Delta E_{k}^{\left(\exp\right)}}\right)}^{2}}\times 100\%$$

where *k* enumerates the molecular species for which we have both experimental and corresponding theoretical data, *K* is the total number of such data points, and upper indexes “exp” and “theor” denote experiment and theory, respectively. As the tunneling splittings are rather sensitive to the quality of the PES and to the nuclear motion computational model, the more reasonable expectation is to obtain the order of magnitude of the splitting. To characterize this match of the order of magnitude, we also computed the logarithmic version of the RMSRE (lRMSRE) defined as

3 $$lRMSRE=\sqrt{\frac{1}{K}\overset{K}{\underset{k=1}{\sum }}{\left(\frac{\ln\left(\Delta E_{k}^{\left(\exp\right)}\right)-\ln\left(\Delta E_{k}^{\left(theor\right)}\right)}{\ln\left(\Delta E_{k}^{\left(\exp\right)}\right))}\right)}^{2}}\times 100\%$$

**Table 2:** Comparison of Experimental and Theoretical Tunneling Splittings (Δ*E*)

	Δ*E* [MHz]				
Cluster/Metric	Experiment	RPIT^b^	E1DSE^c^	BMTD^d^	ERM^e^
FA-FA	341.0(28)^38^	248	196	108	
FA-PA	291.428(5)^18^	207	246	83	
FA-FA@PhF	267.608(1)^37^	312	45	36	147
FA-PA@PhF^a^	50.8949(61)	69	2	9	34
FA-PA@PhF···He^a^	47.579(38)	60	45		46
					
PCC		0.90	0.77	0.87	
RMSRE, %		56	74	83	
lRMSRE, %		6	40	32	

a Values are given for conformer #1.

b Theoretical values were produced by RPIT calculations at the CCSD­(T)-F12-RI/cc-pVDZ-F12//B3LYP-D3­(BJ)/def2-TZV level of theory.

c Theoretical values were computed with a one-dimensional model using mass-dependent at the composite level of theory DLPNO­(TightPNO)-CCSD­(T)/aug-cc-pVTZ//B3LYP-D3­(BJ)/def2-TZVPP for the double proton reaction path, obtained from the nudged elastic band (NEB) calculations at the B3LYP-D3­(BJ)/def2-TZVPP level of theory. Theoretical approach is described in refs 54 and 55 . Details on simulations are available in SI.

d Theoretical values were computed with a one-dimensional Schrödinger equation with free-energy potentials obtained from BMTD simulations at the PBEh-3c level of theory. Details are available in SI.

e ERM model was used to extrapolate from the experimental splitting values for FA-FA, FA-PA, and FA-PA@PhF to FA-FA@PhF, FA-PA@PhF, and FA-PA@PhF···He*a*, respectively. This was done by using eq 7 and theoretical reorganization energies and effective vibrational frequencies from Table 3. As the ERM values extrapolate the given experimental splitting to the corresponding value for the perturbed system, the whole set of values is not self-consistent; therefore, a statistical analysis of this set of semiexperimental values was not performed.

The resulting comparison of the experimental and the theoretical tunneling splittings, as well as PCC, RMSRE, and lRMSRE values, is given in Table 2. The RPIT approach performs best among all tested methods, yielding a very high correlation between the experimental and theoretical values (PCC = 0.90). While the actual values from RPIT can be almost 50% off from the experimental values, according to the RMSRE, the order of magnitude of the splitting is always restored with high precision, shown by the lRMSRE value of only 6%. Given such a good match, we used the RPIT to take a deeper look at the observed trends in our molecular clusters.

Instanton represents the minimum action tunneling pathway between two degenerate wells, and the tunneling splitting is given by an equation

4 $$\Delta E_{RPIT}=\frac{2\hbar }{\Phi }\sqrt{\frac{S}{2\pi \hbar }}\exp\left(-\frac{S}{\hbar }\right)$$

where *S* is the Euclidean action of the instanton and Φ is a measure of the fluctuations around the instanton. One can see a clear resemblance of eq 4 to eq 1. The two main quantities that determine the splitting size are *S* and Φ (eq 4), of which the former is determined by the tunneling path length and the barrier along it, while the latter is primarily influenced by the orthogonal vibrational mode(s) coupled to the instanton. The contribution of each atom to the tunneling process is quantified by calculating its squared mass-weighted tunneling path length (SMWTPL) derived from the instanton trajectory. These values are then normalized as percentages relative to the total SMWTPL across all atoms. For FA-PA@PhF, the instanton trajectory reveals that the attached PhF participates in the tunneling process (Figure 4A), contributing 14% to the SMWTPL. As a result, the tunneling path length is greatly increased, thereby increasing the action of the instanton path compared to pure FA-PA. We also note that the weak interactions from the PhF adsorbate impact Φ as well, suggesting that fluctuations, due to soft adsorbate vibrational modes, are crucial in determining tunneling dynamics, underscoring the significance of multidimensional effects. The effect of the exponential term *S* is more pronounced; hence, the tunneling splitting is dramatically reduced from 207 to 69 MHz upon adsorption of FA-PA onto PhF. When a helium atom is attached as a top layer of this ”quantum sandwich”, RPIT predicts that the helium atom has little influence on the energy and length of the tunneling path, contributing 0.5% of the squared mass-weighted tunneling path length (Figure 4B), only slightly affecting *S*. However, this still results in a slightly smaller tunneling splitting of 60 MHz.

**Figure 4:**
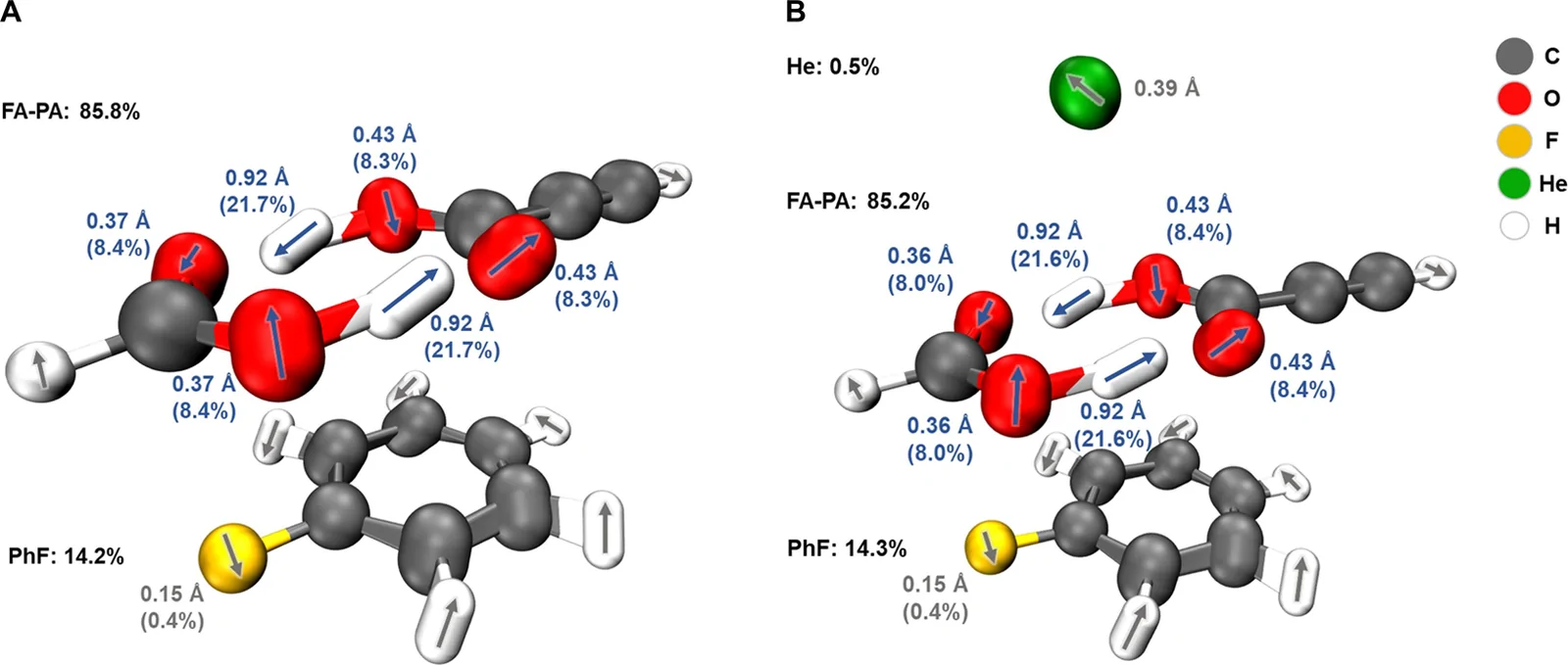
RPIT tunneling trajectories of conformer #1 for (A) FA-PA@PhF and (B) FA-PA@PhF···He. Arrows indicate the tunneling direction, the values (Å) give atomic displacements, and the percentages show the contributions of atoms/molecules/fragments to the total squared mass-weighted tunneling path length (SMWTPL).

The E1DSE, although also a full-dimensional approach like the RPIT, uses several approximations that make it significantly less accurate than the RPIT. First, all the bath degrees of freedom, orthogonal to the proton transfer coordinate, are treated at the harmonic approximation, which automatically disregards the anharmonic effects for these motions. Second, the bath is always taken to be in the ground vibrational state, treated in the adiabatic approximation, which ignores vibrational nonadiabatic effects that become important for low-frequency motions. Third, the computational procedure for computing the effective mass along the motion, which encapsulates the nonlinear nature of the reactive coordinate, is numerically cumbersome and introduces additional numerical noise. Taking these facts into consideration, we can now explain that, despite using a similar level of theory in the E1DSE case as in the RPIT calculations, we observe a reduction in correlation between experiment and theory from high to moderate (PCC = 0.77) and a further increase in relative deviations of tunneling splitting values (RMSRE = 74%). Nevertheless, we see that E1DSE can still be applied to swiftly estimate the order of magnitude for the tunneling splittings (with lRMSRE = 40%).

In addition to these two known tunneling splitting computation approaches, we also applied another way of computing the proton transfer tunneling splitting by solving the one-dimensional Schrödinger equation with free-energy potentials obtained from BMTD simulations.^68^ Unlike the standard metadynamics, the BMTD also provides the value of the reduced mass of the sampled motion, which allows us to directly set the Schrödinger equation to solve after the free-energy bias potential is sampled. As a justification for using free energy as a bath-averaged PES, we imagined that thermal motion along low-frequency delocalized bath modes resembles quantum fluctuations, whereas the local high-frequency modes are less important to the reaction dynamics due to their local character and adiabatic separation, given their much shorter vibrational periods. Therefore, we performed the BMTD simulations at 100 K to reduce the probability of dissociation of our weakly bound clusters and to emulate zero-point vibrational effects for vibrations with frequencies below 70 cm^–1^. In the BMTD, we could not use a correction of the reaction pathway with high-level quantum-chemical calculations for the sampled bias potential, and we also needed to keep the computational cost moderate to produce sufficient numbers of metadynamics trajectories. Thus, we turned to the PBEh-3c^69^ model, which showed good performance for double proton transfer reactions in carboxylic acid dimers using the E1DSE model.^37^ A detailed description of the theoretical calculations performed is available in the SI. As we can see from Table 2, the relative deviation of the resulting splittings in the BMTD case is worse than for the E1DSE due to much lower splitting values (RMSRE = 83%). However, the trend of the splittings is restored correctly, with a rather high PCC value of 0.87, and the order of magnitude is restored to the same level as for the E1DSE, as can be seen from lRMSRE = 32%.

In general, all the tunneling-splitting calculations we performed here followed the recommended best-practice protocols for quantum-chemical methods.^70^ Therefore, despite the different levels of theory in each case due to technical and software limitations, the results from RPIT, E1DSE, and BMTD calculations are, in general, comparable and show a general correlation between the experiment and theoretical values. The RPIT, as the method with the least theoretical approximations, outperforms both E1DSE and BMTD, which showed rather similar quality of tunneling splittings prediction. In general, the BMTD simulations were the most computationally expensive among the RPIT and E1DSE models, as they require multiple long *ab initio* molecular dynamics trajectories. Unlike the other two methods, the BMTD splittings also cannot be improved by using the higher-accuracy single-point energies. However, BMTD is agnostic to the conformational state of the system and can therefore be easily used for large molecular ensembles (1000 or more atoms) with reactive force fields or machine-learned potentials.^71,72^

Despite significantly different tunneling splittings were observed for our molecular clusters, both experimentally and theoretically, according to the reaction path calculations with the nudged elastic band (NEB) method^73^ at the B3LYP-D3­(BJ)/def2-TZVPP level of theory,^46–48^ the proton transfer reaction barriers in all species are the same, namely 8 kcal/mol; therefore, the barrier change does not account for the observed slowing down of the proton transfer rate. We can also see that the decrease in experimental Δ*E* values strongly anticorrelates with the number of atoms in the molecules (PCC = – 0.85), which suggests that the tunneling rate slows down with the increasing complexity of the environment. However, this dependency is not as straightforward, e.g., it is structure-dependent. To check for that, we have also computed the tunneling splittings of conformer #2 of FA–PA@PhF and FA–PA@PhF··· He with RPIT, yielding values of 20 and 13 MHz, respectively. These values are several times smaller than those of conformer #1. Therefore, the slowdown of the tunneling rate depends not only on the size of the system but also on the geometric arrangement.

While RPIT provides a detailed explanation of the observed trends, this analysis is rather complicated. Therefore, to more simply rationalize the observed environmental slowing of proton tunneling, we turned to the ERM, in which proton transfer is linearly coupled to the environment-bath modes. It was first introduced in ref. 37 in the case of one environmental mode coupled to the proton transfer. Here, we extended it to an arbitrary number of coupled motions, obtaining the following expression for the proton transfer splitting in the vibrational ground state in the second-order perturbation theory approximation (see SI for detailed derivation)

5 $$\Delta E_{ERM}=\Delta E_{0}\left(1-\overset{N}{\underset{i=1}{\sum }}\frac{\lambda_{i}}{2hc{\mathrm{\nu ̃}}_{i}}\right)$$

where Δ*E*_0_ and Δ*E*_ERM_ denote the bare and environment-coupled tunneling splittings, respectively, λ_*i*_ are the mode-specific reorganization energies, ${\mathrm{\nu ̃}}_{i}$ are the associated vibrational frequencies of the bath modes, given in wavenumbers, and *c* is the speed of light. The reorganization energy λ in the proton transfer case can be imagined as a payoff for a proton transfer with an unrelaxed position along the environment bath mode. From this model, it can be deduced that the more degrees of freedom are involved in the reaction, the slower the proton transfer will proceed. The relationship between the ERM (eq 5) and the general semiclassical expression for tunneling splitting (eq 1) appears the least straightforward. However, based on the ERM’s fundamental assumption that the frequency of the proton transfer in a given potential well remains unchanged, the correction proportional to reorganization energies can be ascribed to the change in action *δS*, which is expressed via the approximation exp­(−*δS*/*ℏ*) ≈ 1 – *δS*/*ℏ*. In this regard, the ERM provides an explicit expression for the change in action of proton transfer due to the environmental relaxation as

6 $$\delta S=\overset{N}{\underset{i=1}{\sum }}\frac{\lambda_{i}}{4\pi c{\mathrm{\nu ̃}}_{i}}$$

The change in action as the main source of trends in the system-dependent tunneling splitting also agrees with the RPIT results (see ).

While the environment rearrangement can be clearly seen in the splitting calculations we performed, e.g., in the RPIT tunneling trajectories (Figure 4), the practical application of eq 5 for our molecular systems requires knowledge of all coupling energies and vibrational frequencies for all vibrational modes, which is computationally challenging to obtain because the internal coordinates can be parametrized and treated in multiple ways. Therefore, instead of determining the mode-specific reorganization energies, we use the representative mean frequency ⟨ν̃⟩ to extract the semiexperimental mean environmental reorganization energy ⟨λ⟩ for a given molecular moiety. In our sandwich clusters, the two moieties of interest are the PhF molecule and the helium atom. The vibrational frequencies were extracted from theoretical calculations by examining the shapes of harmonic vibrations and averaging the vibrational frequencies associated with the reorganization of given parts of the molecule. The extraction of relevant frequencies was performed by manual inspection of the shapes of the normal modes (the chosen vibrations are shown in the SI). With these definitions, eq 5 yields the effective reorganization energy, as shown below:

7 $$\left\langle \lambda \right\rangle =2hc\left\langle \mathrm{\nu ̃}\right\rangle \left(1-\frac{\Delta E}{\Delta E_{0}}\right)$$

where the tunneling splittings come from the experiment, and the mean vibrational frequency is obtained from quantum-chemical calculations (details are given in SI). To extract these reorganization energies from eq 7, pairs of clusters with and without a given molecular fragment are constructed. We have considered three cases for extracting ⟨λ⟩ values. The first one was the reorganization of PhF moiety in FA-FA@PhF, in which case Δ*E* was for FA-FA@PhF, while Δ*E*_0_ was for FA-FA. The second one was the reorganization PhF moiety in FA-PA@PhF, in which case Δ*E* was for FA-PA@PhF, while Δ*E*_0_ was for FA-PA. The third case was the reorganization of helium in FA-PA@PhF··· He, in which case Δ*E* was for FA-PA@PhF··· He, while Δ*E*_0_ was for FA-PA@PhF.

The resulting effective reorganization energies are summarized in Table 3. The values are consistent with chemical intuition. The PhF reorganization energies in FA-PA@PhF and FA-FA@PhF are of the same order of magnitude ( ∼10 cm^–1^), whereas for He the reorganization energy is 2 orders of magnitude smaller ( ∼1 cm^–1^), reflecting its much weaker dispersion interaction due to the low polarizability. Furthermore, the reorganization energy of PhF in FA-PA@PhF is about three times larger than in FA-PA@PhF, consistent with the stronger interaction of the FA-PA fragment with PhF compared to FA-FA (see Table S16 in the SI for SAPT analysis).

**Table 3:** Semiexperimental (⟨*λ*⟩_se_) and Theoretical (⟨*λ*⟩_t_) Effective Reorganization Energy Values and the Corresponding Theoretical Effective Environment Frequencies ⟨ν̃⟩

	Value, cm^–1^		
	PhF in FA-FA	PhF in FA-PA	He in FA-PA@PhF
⟨ν̃⟩	27	22	22
⟨λ⟩_t_	31	39	5
⟨λ⟩_se_	12	36	5

To compare the semiexperimental reorganization energy values with the quantum-chemical analogs, we performed the intrinsic reaction coordinate (IRC) scans for FA-FA@PhF and conformers #1 of FA-PA@PhF and FA-PA@PhF···He clusters from the transition states (TS) of the proton transfer reaction at the B3LYP-D3­(BJ)/def2-TZVP level of theory. The IRC calculation yielded slightly different structures for the forward and backward directions, as the TS is not perfectly symmetric. The backward scan structures were exactly the global minimum structures, whereas the forward scan structures were higher-energy minima, differing in the orientations of the PhF and He moieties. Upon optimization of these higher-energy structures of FA-PA@PhF and FA-PA@PhF···He clusters at the B3LYP-D3­(BJ)/def2-TZVPP level of theory, we found two additional conformers, where the positions of FA-PA and FA-PA···He moieties are suboptimal with respect to the PhF fragment. For the FA-FA@PhF, however, the forward structure of the IRC scan converged to the mirror image of the conformer #1; thus, for FA-FA@PhF, the two ends of the IRC were used as estimates for environment-relaxed and unrelaxed structures. The energy differences between these structures can thus be interpreted as the reorganization energy of the PhF for the FA-FA@PhF and FA-PA@PhF clusters, and the sum of PhF and He reorganization energies for the FA-PA@PhF···He cluster. For these pairs of geometries of FA-FA@PhF, FA-PA@PhF, and FA-PA and FA-PA···He clusters at the B3LYP-D3­(BJ)/def2-TZVPP level of theory, we computed the single-point energies at the DLPNO­(TightPNO)-CCSD­(T1)/aug-cc-pVTZ level of theory, and their differences were taken as theoretical reorganization energies ⟨λ⟩_t_, summarized in Table 3. The resulting energies show good agreement with their semiexperimental counterparts (PCC = 0.82). We also used the theoretically computed effective frequencies and theoretical reorganization energies to extrapolate from the experimental tunneling splitting values of unsubstituted molecular clusters to their substituted counterparts (Table 2). The resulting corrected values are close to their experimental counterparts, demonstrating the potential of this approach for aiding experimental data assignment and experimental design.

In summary, we have spectroscopically resolved and characterized the concerted double proton transfer tunneling in two sandwich-like molecular clusters: the ternary cluster FA-PA@PhF and the quaternary cluster FA-PA@PhF···He. The experimentally determined tunneling splittings are 50.8949(61) MHz and 47.579(38) MHz, respectively. This systematic reduction in tunneling splitting, compared to the FA-PA dimer (291.428(5) MHz)^18^ and the previously reported FA-FA@PhF cluster (267.6080(13) MHz),^37^ demonstrates that the surrounding molecular environment exerts a profound and multidimensional modulating effect on proton transfer dynamics. Our integrated spectroscopic and theoretical analysis reveals that the underlying mechanism is not merely a simple perturbation of the minimum energy path or barrier height. Instead, the modulation occurs through concerted multidimensional coupling: environmental molecular moieties (e.g., PhF) participate synchronously in the tunneling motion, increasing the effective tunneling path length (as evidenced by the increased tunneling action) while the intrinsic barrier remains largely unchanged. Furthermore, our theoretical results indicate a significant conformational dependence of the tunneling rate, even for isoenergetic or quasi-degenerate conformers (e.g., Conformer #1 vs #2). This suggests that the spatial arrangement of the environment, not just its chemical identity, is a critical factor in quantum tunneling dynamics. These findings provide a deeper, mechanistic understanding of environmental effects on quantum tunneling in multicomponent systems. They highlight the potential for strategic control of proton-transfer kinetics and coherence through precise manipulation of the surrounding molecular architecture, a principle with potential implications for fields ranging from catalysis and biochemistry to molecular quantum sensing and information processing.

## Supplementary Material





## Notes

NEB, SAPT, metadynamics, and tunneling splitting calculation results files, along with the corresponding experimental fitting files and associated scripts (ZIP)
Supporting text, Figures S1–S11, Tables S1–S27, and references (PDF)
