# Supplementary material for: Environment Rearrangement Slows Down the Tunneling Rates of Proton Transfer in Sandwich-Like Molecular Clusters
Source: J Phys Chem Lett. 2026 Jul 30;17(32):9112–21. doi: 10.1021/acs.jpclett.6c01033 (PMC13488451; doi:10.1021/acs.jpclett.6c01033)
Supplement: Supplementary file 1 [file jz6c01033_si_002.pdf]

Supporting Information for

Environment rearrangement slows down the tunneling rates of proton transfer in sandwich-like molecular clusters

Jingling Hong, Ziye Qi, Yao Wang, Denis S. Tikhonov, Wei Fang, Xiao Zheng, Mingfei Zhou, and Weixing Li

*Corresponding authors:
D.S. Tikhonov (denis.tikhonov@desy.de)
W. Fang (wei_fang@fudan.edu.cn)
W. Li (weixingli@fudan.edu.cn)

This PDF file includes:

Supporting Text
Figs. S1 to S17
Tables S1 to S29

Contents

S1 Methods **S3**
S1.1 Details of experimental instrument and method . . . S3
S1.1.1 Materials and instrument . . . S3
S1.1.2 Experimental procedure and spectra . . . S3
S1.2 Theoretical calculations . . . S4
S1.3 Marcus-type theoretical model of the proton transfer in the coupled environment . . S5
S1.3.1 Vibrational modes employed in the simplified linear Marcus-type model S7

S2 Theoretical calculations for the stable structures of FA-PA@PhF complex **S8**

S3 Theoretical calculations for the stable structures of FA-PA@PhF···He complex **S10**

S4 Experimental parameters of the FA-PA@PhF complex and its isotopologues **S14**

S5 Substitute structure of FA-PA@PhF **S16**

S6 Experimental parameters of the FA-PA@PhF···He complex **S17**

S7 The calculation results of IGMH analysis **S18**

S8 The calculation results of SAPT analysis **S19**

S9 Comparison of experimental tunneling splitting energy and tunneling period **S20**

S10 Instanton theory calculations **S21**
S10.1 Theoretical details . . . S21
S10.2 Reorganization energy estimation from the intrinsic reaction coordinate optimization S28

S11 Rotational transitions of the FA-PA@PhF complex and its isotopologues **S30**

S12 Rotational transitions of the FA-PA@PhF···He complex **S43**

S13 Metadynamics simulations **S48**

S1 Methods

S1.1 Details of experimental instrument and method

S1.1.1 Materials and instrument

We purchased commercial samples, including formic acid from Aladdin (purity $\geq$ 98%), fluorobenzene from Aladdin (purity $\geq$ 99%), and propiolic acid from Admas-beta (purity $\geq$ 98%). We also use formic acid-d_2 from Isotope (purity $\geq$ 98%) for deuterium substitution experiments. Due to the high purity of the samples, which meet the experimental requirements, all samples were used without further purification. For experiments, we use the chirped-pulse Fourier transform microwave (CP-FTMW) spectrometer at Fudan University to acquire microwave spectroscopy signals. The spectrometer primarily consists of a 50 GS/s arbitrary waveform generator, two kinds of power amplifiers: a 300 W traveling wave tube amplifier (TWTA) and a 200 W solid-state power amplifier (SSPA), a pair of broadband horn antennas, a limiter, a radio-frequency coaxial switch, a low-noise microwave signal amplifier, an oscilloscope, and a rubidium atomic clock, following a similar operating principle as reported previously[1,2]. The instrument can operate in either the 2–8 GHz or 8–16 GHz range by switching configurations. According to maginitudes of the theoretical constants and cluster size, we select the 2–8 GHz range as the optimal window for detecting the FA-PA@PhF and FA-PA@PhF$\cdots$He complexes.

S1.1.2 Experimental procedure and spectra

The microwave spectra of the samples, including fluorobenzene, formic acid, and formic acid-d_2, were measured using a chirped-pulse Fourier transform microwave (CP-FTMW) spectrometer. These samples were directly mixed in a sample container connected to the gas line, with helium used as the carrier gas. The helium stream passed over the sample container and subsequently expanded into the vacuum chamber (5×10^{-5} Pa) through a 1 mm diameter pulsed nozzle operating at 8 Hz. Simultaneously, the propiolic acid sample was placed in a custom-made reservoir and heated to 50°C to increase its vapor pressure prior to supersonic expansion. During the expansion, the molecules and their complexes were rotationally cooled to 1–2 K, allowing the most energetically favorable configurations to stabilize under the given conditions. After a delay of approximately 910 μs, six back-to-back chirped pulses, each 4 μs in duration and spanning the 2–8 GHz frequency range, were broadcast into the vacuum chamber via a horn antenna to interact with each molecular beam pulse. These pulses were generated using an arbitrary waveform generator (AWG) and amplified by a traveling wave tube amplifier (TWTA). Subsequently, six free induction decay (FID) signals, reflecting the macroscopic dipole moment of the molecular ensemble, were collected over 40 μs using another horn antenna. The signals were amplified, recorded in the time domain with a digital oscilloscope, and converted into the frequency domain using a fast Fourier transform (FFT). To improve measurement efficiency and reduce sample consumption, the oscilloscope's fast-frame acquisition mode was employed, allowing six excitation and detection cycles per supersonic expansion.

The resulting spectra exhibited high signal-to-noise ratios after long-term accumulation, with frequency accuracy better than 15 kHz and spectral resolution better than 25 kHz. For the PA-FA@PhF and its deuterated species, 2.6 million FID signals were collected, while for the larger cluster PA-FA@PhF$\cdots$He, a total of 12.1 million FID signals were acquired in the time domain. The resulting spectra exhibit rich structural information: for the former, approximately 3,600 spectral lines with intensities above 0.00015 μV were observed; for the latter, about 4,500 lines with intensities above 0.0008 μV were detected. To simplify spectral analysis, we removed the overlapping signals with the previously measured FA-PA spectrum from our lab, as well as signals corresponding to the fluorobenzene monomer and the PhF–He dimer. The processed spectra are presented in Fig. S1, containing signals of the two clusters FA-PA@PhF and FA-PA@PhF$\cdots$He.

Further spectral analysis was conducted using the AABS package[3] and Pickett's SPFIT program[4]. For FA-PA@PhF, FACD-PA@PhF, and FA-PA@PhF$\cdots$He, 203, 160, and 232 transitions,

respectively, were fitted into a two-state proton transfer-rotation coupled Hamiltonian:

$$\mathbf{H} = \begin{pmatrix} \mathbf{H}^{\mathrm{R}}_{0+} + \mathbf{H}^{\mathrm{CD}} & \mathbf{H}_{\mathrm{int}} \\ \mathbf{H}_{\mathrm{int}} & \mathbf{H}^{\mathrm{R}}_{0-} + \mathbf{H}^{\mathrm{CD}} + \Delta E \end{pmatrix}, \tag{1}$$

with

$$\mathbf{H}_{\mathrm{int}} = F_{ab}(\mathbf{P}_a\mathbf{P}_b + \mathbf{P}_b\mathbf{P}_a) + F_{bc}(\mathbf{P}_b\mathbf{P}_c + \mathbf{P}_c\mathbf{P}_b) + F_{ca}(\mathbf{P}_c\mathbf{P}_a + \mathbf{P}_a\mathbf{P}_c), \tag{2}$$

Here, $\mathbf{H}^{\mathrm{R}}_{0+}$ and $\mathbf{H}^{\mathrm{R}}_{0-}$ are the semirigid rotor terms for two splitting substates, $\mathbf{H}^{\mathrm{CD}}$ denotes centrifugal distortion terms, ΔE is the tunneling splitting between energy eigenstates 0^+ and 0^-, $F_{\alpha\beta}$ $(\alpha, \beta = a, b, c)$ are the Coriolis coupling terms, and $\mathbf{P}_\alpha$ are the angular momentum operators.

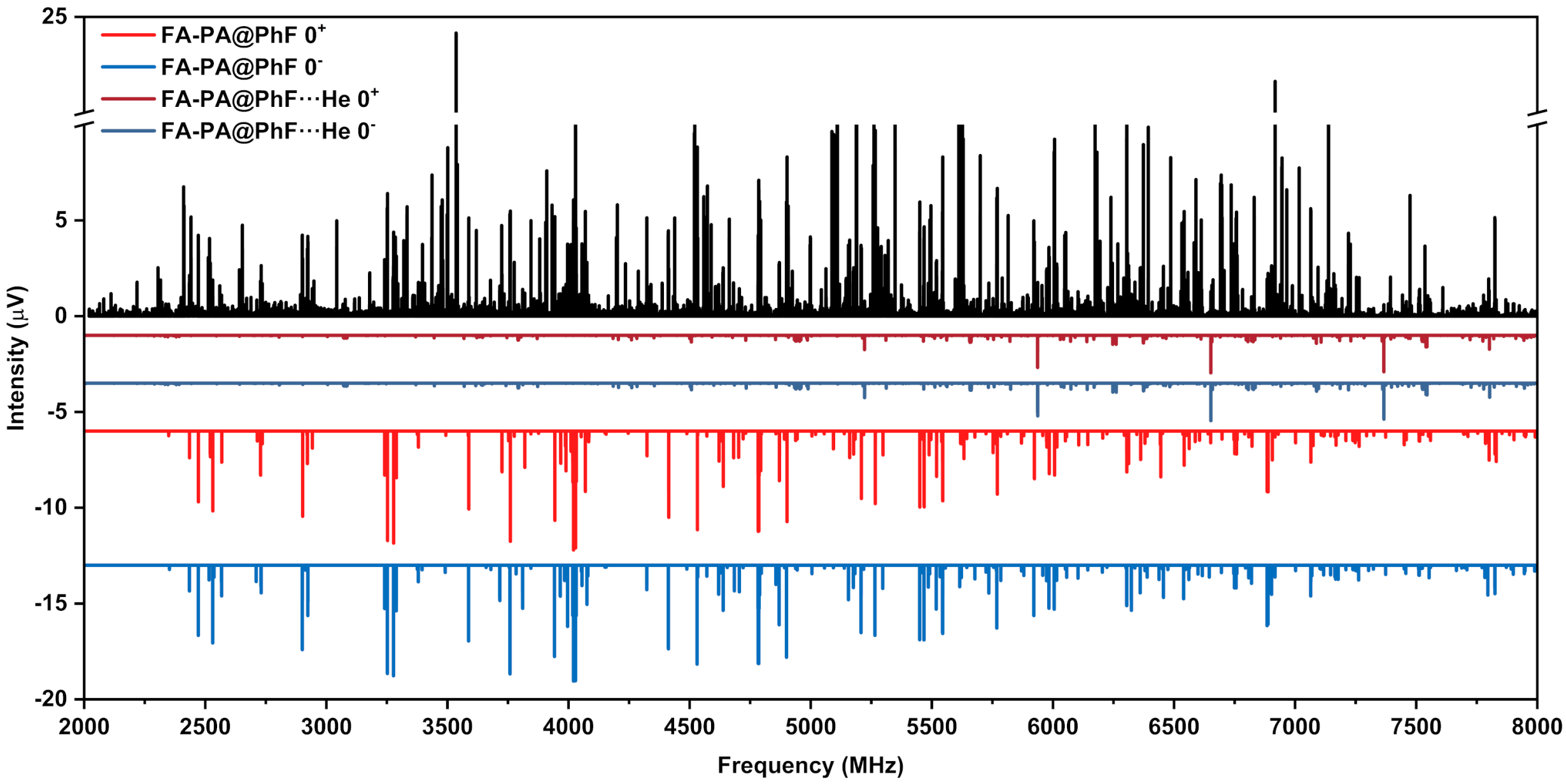

Figure S1: Broadband rotational spectra of FA-PA@PhF and FA-PA@PhF···He. The black upper trace corresponds to the simplified experimental spectrum (12.1 million FID average), obtained by mixing FA, PA, and PhF samples and using helium as the carrier gas. The lower traces in different colors represent the simulated spectra of FA-PA@PhF and FA-PA@PhF···He, generated from the experimental spectroscopic parameters.

S1.2 Theoretical calculations

The theoretical search for stable geometries was performed with the CREST software[5], an efficient tool for creating and analyzing structure ensembles. The resulting conformers were optimized using B3LYP-D3(BJ)/def2-TZVPP method[6,7,8,9,10] sets with the ORCA 5[11] quantum chemical suite. Harmonic frequency calculations were performed to obtain the zero-point energies (ZPE), and the basis set superposition error (BSSE) in the cluster binding energies was corrected using the counterpoise method at the same level of theory. The symmetry-adapted perturbation theory (SAPT) calculation was performed using PSI4[12], with a 2+(3) approximation and the aug-cc-pVDZ basis set[13]. The proton transfer pathways for the FA-FA, FA-PA, FA-FA@PhF, and FA-PA@PhF were computed using the nudged elastic band (NEB) approach[14] using the same quantum chemical method. For each of the images in the optimized path, a Hessian at the B3LYP-D3(BJ)/def2-TZVPP and a single point energy at the DLPNO(TightPNO)-CCSD(T)/aug-cc-pVTZ level of theory were computed[15,16,17].

Here, we investigate the interplay of changes in both the external and internal molecular environments on the model reaction of proton transfer. The symmetric double proton transfer in the carboxylic acids is described via a formal reaction:

$$\mathrm{X1COOH1 + H2OOCX2 \leftrightarrow X1COOH2 + H1OOCX2}\ , \tag{3}$$

where H1 and H2 are the exchanged protons, and X1, X2 are the substituents, or the intramolecular environment. The investigated systems are composed of formic acid (FA, X=H), propiolic acid (PA, X=CCH), fluorobenzene (PhF), and helium atom: the formic acid dimer (FA-FA), the mixed dimer of formic acid with propiolic acid (FA-PA), these two dimers complexed with fluorobenzene (FA-FA@PhF and FA-PA@PhF), and FA-PA sandwiched between fluorobenzene and helium (He@FA-PA@PhF). Replacing hydrogen in the FA for the ethynyl group changes the molecular environment in the direct vicinity of the reactive center, while the complexation with the PhF and He changes the weakly-bounded intermolecular environment.
The resulting tunneling splittings were computed based on the effective one-dimensional Schrödinger equation[18]

$$\left(-\frac{\hbar^2}{2}\frac{d}{d\xi}\frac{1}{\mu(\xi)}\frac{d}{d\xi} + V(\xi) + \mathrm{ZPVE}(\xi)\right)\psi = E\psi(\xi) \ , \tag{4}$$

$$\xi = \frac{r_1(\mathrm{OH}) + r_2(\mathrm{OH}) - r_1(\mathrm{O}\cdots\mathrm{H}) - r_2(\mathrm{O}\cdots\mathrm{H})}{2\sqrt{2}} \ , \tag{5}$$

where E and ψ are the stationary state's energy and wavefunction, V is the high-level potential energy surface, ZPVE is the zero-point vibrational energy of all the rest of vibrational modes excluding the reaction coordinate ξ, and μ is the coordinate-dependent effective mass. ξ is the reaction coordinate for double proton transfer. $r(\mathrm{OH})$ and $r(\mathrm{O}\cdots\mathrm{H})$ denote initially covalent and hydrogen bond distances. The potential energy wells from both sides of the barrier were taken from the harmonic equilibrium potential. The splittings were found as an energy difference between the two lowest vibrational states. To find the ΔE_{free}, the potential of the FA-FA dimer was taken with ZPVE set to zero and μ set to an ideal value of 1 a.m.u.. The calculations were done using scripts from the MOLINC repository (`https://gitlab.desy.de/denis.tikhonov/molinc`). The theoretical details of the approach are discussed in Refs. 18 and 19.

The nudged elastic band (NEB) with DFT potential at B3LYP-D3(BJ)/def2-TZVPP approximation was used to obtain the reaction path for the double proton transfer reaction in four carboxylic acid dimers, including FA-PA, FA-PA@PhF, and FA-PA@PhF$\cdots$He (see Fig. S2).

S1.3 Marcus-type theoretical model of the proton transfer in the coupled environment

The detailed derivation of the model for a single bath mode is given in Ref. 20. Here, we will provide a basic outline of the derivation and extend it to multiple environmental degrees of freedom. The starting point is the system-bath Hamiltonian of the form[20,21]

$$\hat{H}_{\mathrm{sb}} = \hat{H}_{\mathrm{s}}(q) + \overbrace{\sum_j h\nu_j\left(\hat{n}_j + \frac{1}{2}\right)}^{\hat{H}_{\mathrm{b}}} \overbrace{-\frac{1}{2}\sum_j \sqrt{h\nu_j\lambda_j}\cdot q\cdot(\hat{a}_j + \hat{a}_j^\dagger)}^{\hat{W}_{\mathrm{sb}}} = \hat{H}_{\mathrm{s}}(q) + \hat{H}_{\mathrm{b}} + \hat{W}_{\mathrm{sb}} \ , \tag{6}$$

where $\hat{H}_{\mathrm{s}}$ is the system Hamiltonian, describing only the proton transfer reaction, $\hat{H}_{\mathrm{b}}$ is the bath Hamiltonian, consisting of harmonic oscillators describing individual modes of molecular environment, $\hat{W}_{\mathrm{sb}}$ is the system-bath interaction energy, ν_j and λ_k are the harmonic vibrational frequency and reorganization energy, associated with j-th bath mode, $\hat{a}_j^\dagger$ and $\hat{a}_j$ are the creation and annihilation operators for the j-th bath's harmonic mode, $\hat{n}_j = \hat{a}_j^\dagger\hat{a}_j$. The pure system's Hamiltonian has two lowest eigenstates $|\pm\rangle = \frac{1}{\sqrt{2}}(|-\xi_0\rangle \pm |+\xi_0\rangle)$ with eigenenergies $\varepsilon_\pm$, and thus the environment-less splitting $\Delta E_{\mathrm{free}} = \varepsilon_- - \varepsilon_+$. The localized solutions $|\pm\xi_0\rangle$ correspond to the wavepacket localized in the left and right potential wells at the mean positions $\langle\pm\xi_0|\xi|\pm\xi_0\rangle = \pm\xi_0$, and at the same time $\langle\pm\xi_0|\xi|\mp\xi_0\rangle = 0$. Thus, by defining $q = \xi/\xi_0$ as the dimension-less proton transfer coordinate, we get $\langle\pm|q|\pm\rangle = 0$ and $|\langle\pm|q|\mp\rangle| = 1$. Applying the perturbation theory up to the second order[22], the

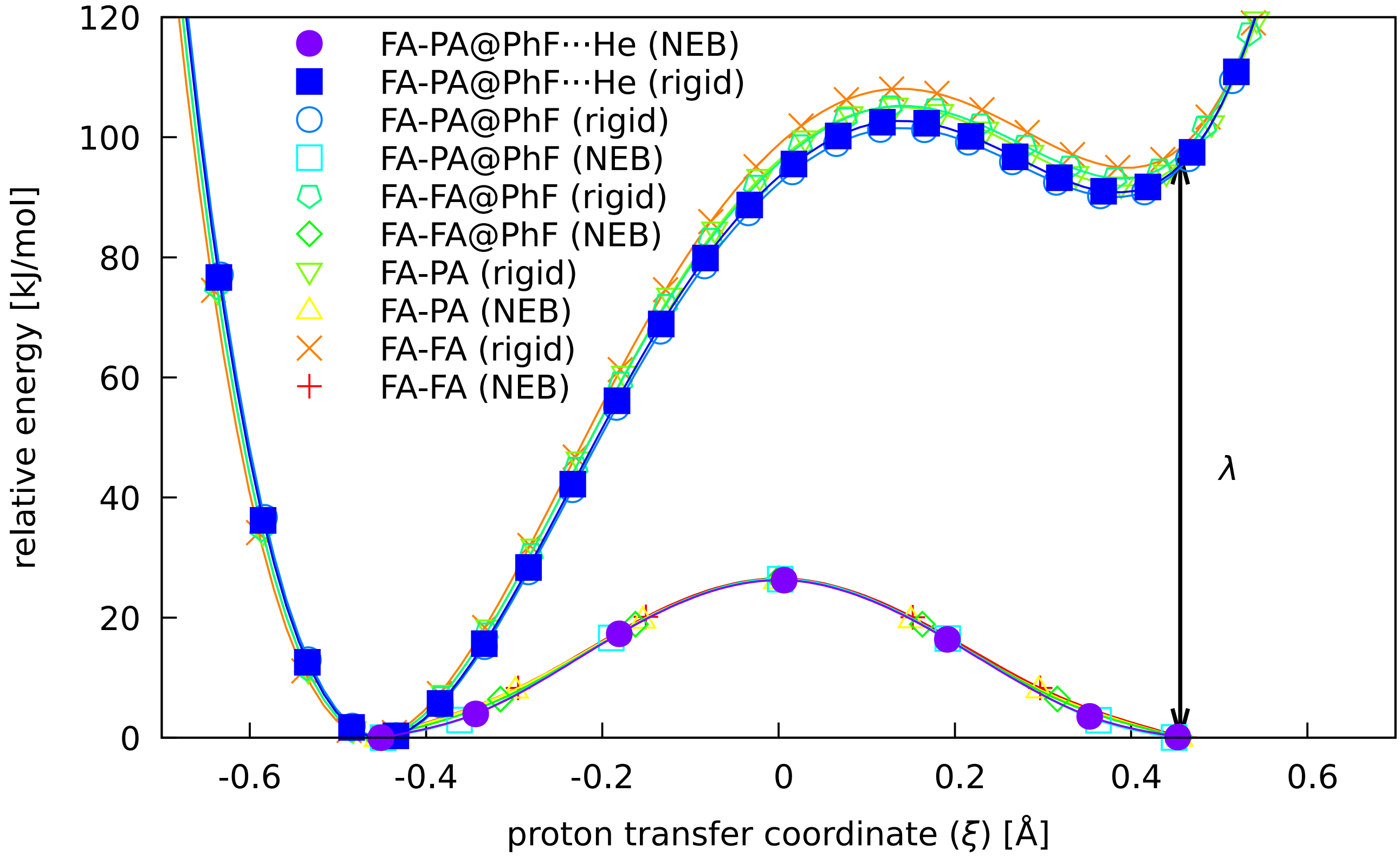

Figure S2: Theoretical cuts through the double proton transfer potential energy surfaces in four carboxylic acid dimers. NEB curves correspond to the fully relaxed reaction path, whereas rigid scans correspond to all degrees of freedom being frozen except for the reaction coordinate. The results are given at the B3LYP-D3(BJ)/def2-TZVPP level of theory.

corrected values ($\tilde{\varepsilon}_\pm$) for the bath ground state of the system become

$$\begin{cases} \tilde{\varepsilon}_- = \varepsilon_- + \sum\limits_j \frac{h\nu_j}{2} - \frac{1}{4}\sum\limits_j \frac{h\nu_j\lambda_j}{h\nu_j - \Delta E_{\text{free}}} \\ \tilde{\varepsilon}_+ = \varepsilon_+ + \sum\limits_j \frac{h\nu_j}{2} - \frac{1}{4}\sum\limits_j \frac{h\nu_j\lambda_j}{h\nu_j + \Delta E_{\text{free}}} \end{cases} . \tag{7}$$

Therefore, the environment-influcenced splitting will be (assuming $h\nu_j \gg \Delta E_{\text{free}}$)

$$\Delta E_{\text{env}} = \tilde{\varepsilon}_- - \tilde{\varepsilon}_+ = \Delta E_{\text{free}} - \frac{\Delta E_{\text{free}}}{2}\sum_j \frac{h\nu_j\lambda_j}{h^2\nu_j^2 - \Delta E_{\text{free}}^2} \approx \Delta E_{\text{free}} \cdot \left(1 - \frac{1}{2}\sum_j \frac{\lambda_j}{h\nu_j}\right) . \tag{8}$$

The assumption $h\nu_j \gg \Delta E_{\text{free}}$ is justified, as our numerical estimate for ΔE_{free} is 0.3 cm^{-1} (7872 MHz), while the lowest vibrational frequency for FA-PA@PhF$\cdots$He cluster at B3LYP-D3(BJ)/def2-TZVPP level of theory is 1.8 cm^{-1}.

For any arbitrary bath state $|\mathbf{n}\rangle$ defined by quantum numbers n_j for j-th bath mode arranged into a vector $\mathbf{n}$, the Eq. 7 are being modified into

$$\begin{cases} \tilde{\varepsilon}_{\mathbf{n}-} = \varepsilon_- + \sum\limits_j h\nu_j\left(n_j + \frac{1}{2}\right) - \frac{1}{4}\sum\limits_j h\nu_j\lambda_j \frac{h\nu_j + (2n_j+1)\Delta E_{\text{free}}}{h^2\nu_j^2 - \Delta E_{\text{free}}^2} \\ \tilde{\varepsilon}_{\mathbf{n}+} = \varepsilon_+ + \sum\limits_j h\nu_j\left(n_j + \frac{1}{2}\right) - \frac{1}{4}\sum\limits_j h\nu_j\lambda_j \frac{(2n_j+1)h\nu_j - \Delta E_{\text{free}}}{h^2\nu_j^2 - \Delta E_{\text{free}}^2} \end{cases} . \tag{9}$$

Therefore, for this given bath state $|\mathbf{n}\rangle$, the splitting between the two proton transfer states will be

$$\begin{aligned} \Delta E_{\text{env}} &= \tilde{\varepsilon}_{\mathbf{n}-} - \tilde{\varepsilon}_{\mathbf{n}+} \\ &= \Delta E_{\text{free}} - \frac{h\nu_j\lambda_j}{2}\sum_j \frac{(n_j+1)\Delta E_{\text{free}} - nh\nu_j}{h^2\nu_j^2 - \Delta E_{\text{free}}^2} \\ &\approx \Delta E_{\text{free}} \cdot \left(1 - \frac{1}{2}\sum_j \frac{(n_j+1)\lambda_j}{h\nu_j}\right) + \frac{1}{2}\sum_j n_j\lambda_j \end{aligned} \tag{10}$$

This expression, in principle, allows the determination of the reorganization energies from measurements of splitting for vibrationally excited states of molecules. To get the thermal-averaged splitting for the bath state at temperature T, one can simply replace the given quantum number n_j in the Eq. 10 by its thermal value given as $\langle n_j \rangle (T) = \left(\exp\left(\frac{h\nu_j}{k_\mathrm{B} T} \right) - 1 \right)^{-1}$, where k_B is the Boltzmann constant.

S1.3.1 Vibrational modes employed in the simplified linear Marcus-type model

Table S1: Vibrational modes with dominant contributions to tunneling, employed in the simplified Marcus-type model and their corresponding calculated coupling strengths.

Mode	$\tilde{\nu}_i$ (cm^{-1})	Mode illustration	$\langle \tilde{\nu} \rangle$ (cm^{-1})	$\langle \lambda \rangle$ (meV)	$\langle \lambda \rangle$ (kJ mol^{-1})
		**FA–FA@PhF**			
1	27.26	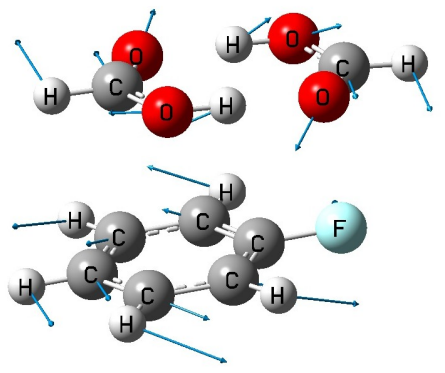	27.26	1.45	0.14
		**FA–PA@PhF**			
1	21.57	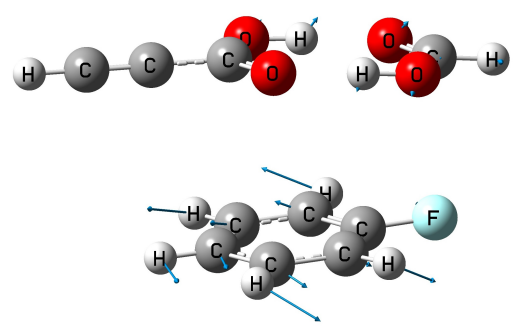	21.57	4.42	0.43
		**FA–PA@PhF$\cdots$He**			
1	18.66	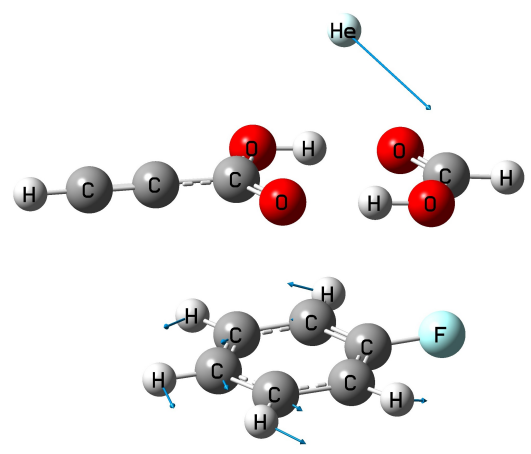			
2	21.74	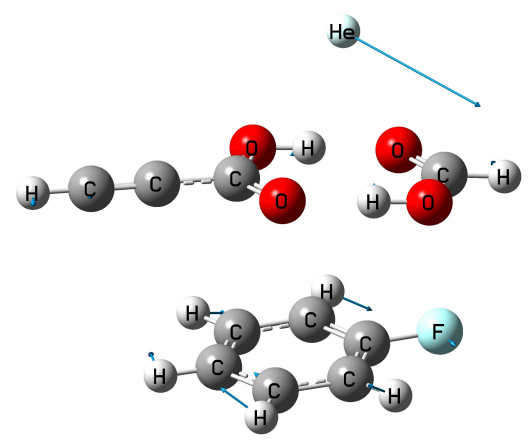	22.21	0.58	0.06
3	26.24	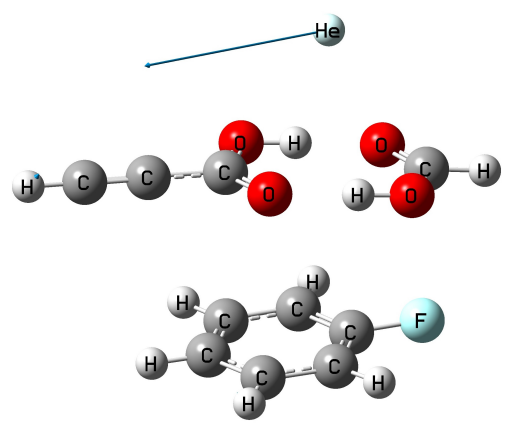			

All vibrational frequencies used to determine $\langle \tilde{\nu} \rangle$ were obtained from harmonic frequency calculations at the minimum-energy structure using the B3LYP-D3(BJ)/def2-TZVPP level of theory.

S2 Theoretical calculations for the stable structures of FA-PA@PhF complex

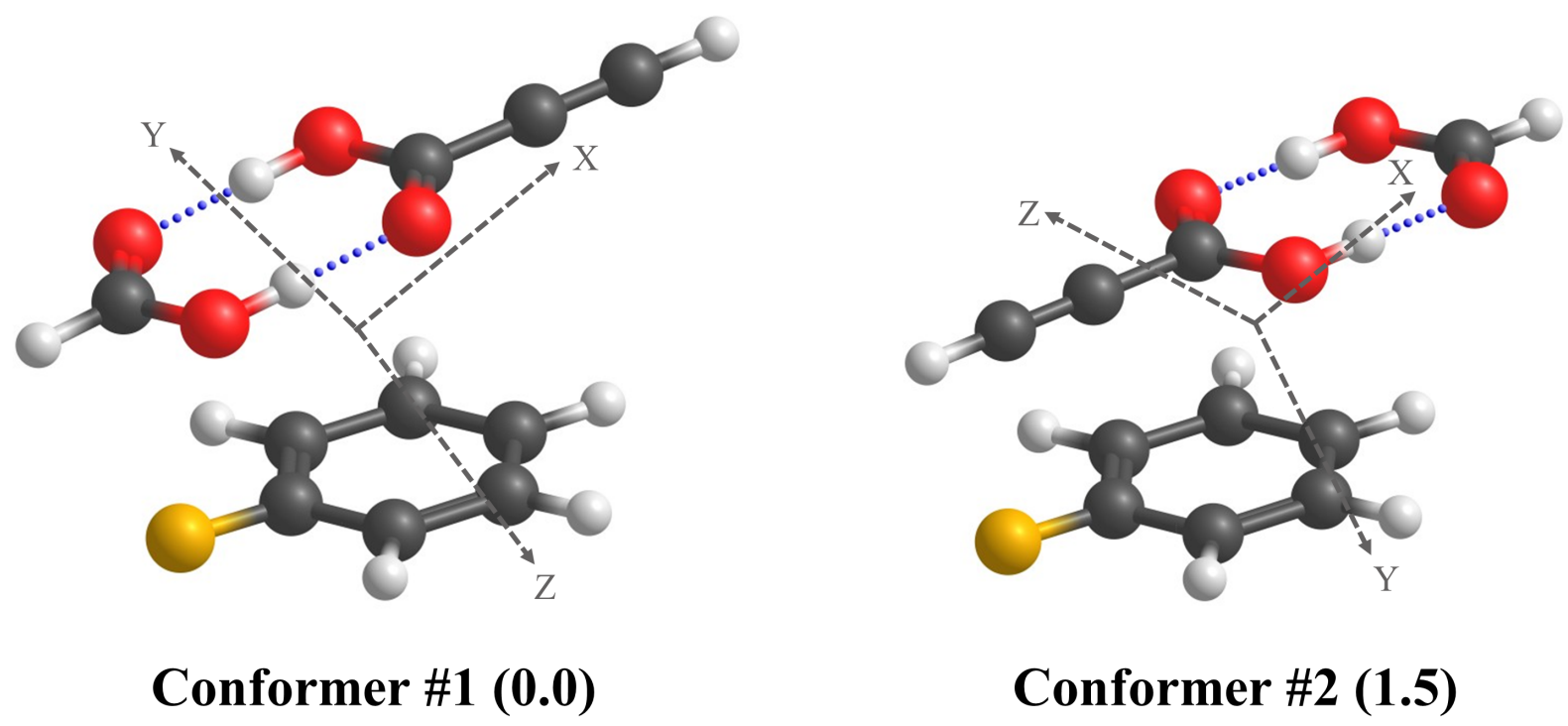

Figure S3: The equilibrium structures of the two lowest conformers of FA-PA@PhF complex at the B3LYP-D3BJ/def2-TZVP level of theory. The numbers in parentheses represent relative energies in kJ/mol after ZPE and BSSE corrections, while the gray dashed lines indicate the principal axes.

Table S2: Rotational constants and dipole moments of the equilibrium structures of the two lowest-energy conformers of the FA-PA@PhF complex at the B3LYP-D3BJ/def2-TZVP level of theory.

	Conformer #1	Conformer #2
A [MHz]	721.8	789.1
B [MHz]	535.8	473.8
C [MHz]	386.3	363.3
$\mu_a/\mu_b/\mu_c$ [D]	1.8/1.2/0.2	0.3/0.5/0.1
ΔE [kJ/mol]	0.0	1.5

Table S3: Cartesian coordinates of the equilibrium structures of the two lowest conformers of FA-PA@PhF complex at the B3LYP-D3BJ/def2-TZVP level of theory.

Conformer #1			
	X	Y	Z
C	4.214775741309	0.020062042569	-0.118374533354
H	5.210332650936	-0.351322211613	-0.161075775873
C	3.093094132149	0.440012125862	-0.070273308303
C	1.741902085570	0.953603607643	-0.061732441569
O	1.111227362331	1.089742993386	-1.100480453414
O	1.305938044376	1.248210407625	1.139288702960
H	0.348219112789	1.560676676927	1.101598104994
C	-1.835814320278	2.173941472211	0.008969728879
H	-2.880739331599	2.501216301562	0.009334461098
O	-1.197484786012	2.062607736709	1.041208540983
O	-1.397448573985	1.935732346016	-1.199334971520
H	-0.444966811185	1.617059189244	-1.178402946554
C	-1.121630393277	-1.584717692837	-1.154027899633
C	0.128365633253	-2.191739189219	-1.150235931017
C	0.810898290621	-2.391044112545	0.044774282306

Continued on next page

	X	Y	Z
C	0.242068975164	-1.980912865011	1.245590385467
C	-1.007775293645	-1.372404859943	1.260787201819
C	-1.662014429067	-1.191510769155	0.056526245058
H	-1.668755573745	-1.406471835031	-2.069328745454
H	0.573828159884	-2.497971447969	-2.087573584917
H	1.786996962688	-2.857174961644	0.039738491802
H	0.775498417014	-2.123965590147	2.176140051853
H	-1.467920385296	-1.032988788042	2.178314801020
F	-2.876717255592	-0.590842088163	0.060827518318

Conformer #2			
	X	Y	Z
C	-2.035350066370	2.885859732527	-0.234129453669
H	-2.979492785311	3.371970100755	-0.289225615220
C	-0.975356820197	2.330205398861	-0.172603279892
C	0.281322255675	1.622929966416	-0.142818227241
O	0.703810307460	1.043041774791	-1.134109025925
O	0.886961762736	1.676006711126	1.020685977944
H	1.727552897896	1.125953531259	1.003171296082
C	3.485603280528	-0.374316684671	-0.035471521864
H	4.411327926164	-0.961938635606	-0.026104723199
O	3.095484115376	0.218254326652	0.955150343629
O	2.897065303947	-0.405055758571	-1.201154477150
H	2.052846785245	0.146337434304	-1.188734696148
C	-1.546894175359	-0.868478485638	1.290011617841
C	-0.323171627560	-1.515340985238	1.422141322670
C	0.257657961189	-2.150142992425	0.330652611318
C	-0.384549753524	-2.135882729058	-0.902861095610
C	-1.607358329345	-1.493664262345	-1.051493534388
C	-2.164655377613	-0.874729562499	0.052450203952
H	-2.015491036598	-0.361935594348	2.122392367798
H	0.178243989836	-1.514685586550	2.381088543711
H	1.209963381713	-2.651902785204	0.438863458626
H	0.071321008195	-2.617782373892	-1.757842105591
H	-2.120459737972	-1.457532041088	-2.002611955194
F	-3.352764867380	-0.245300112902	-0.084631530380

S3 Theoretical calculations for the stable structures of FA-PA@PhF···He complex

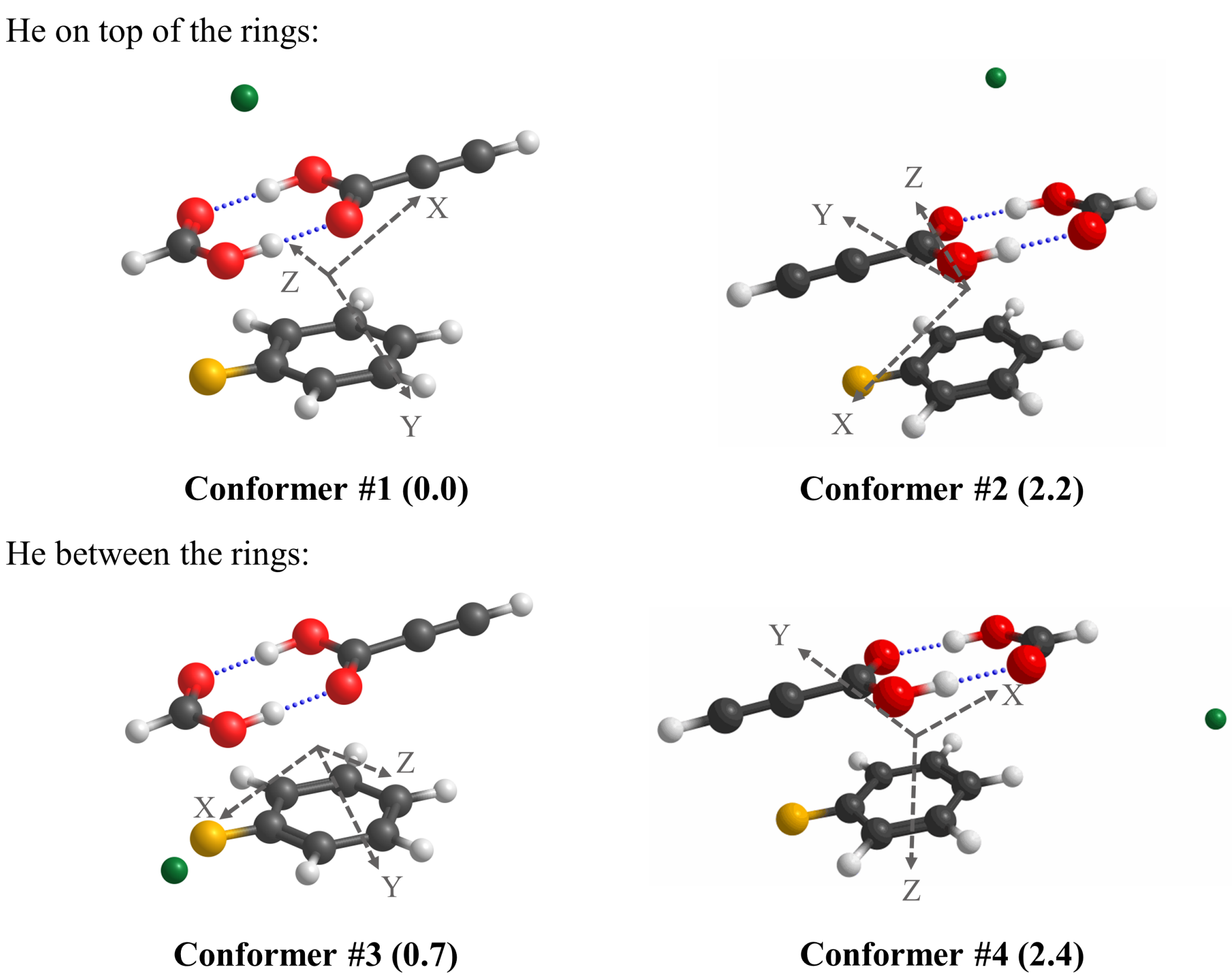

Figure S4: The equilibrium structures of the four lowest conformers of FA-PA@PhF···He complex at the B3LYP-D3(BJ)/def2-TZVP level of theory. The numbers in parentheses represent relative energies in kJ/mol after ZPE and BSSE corrections, while the gray dashed lines indicate the principal axes.

Table S6: Rotational constants and dipole moments of the equilibrium structures of the four lowest conformers of FA-PA@PhF···He complex at the B3LYP-D3(BJ)/def2-TZVP level of theory.

	Conformer #1	Conformer #2	Conformer #3	Conformer #4
A [MHz]	657.1	749.0	705.0	729.0
B [MHz]	531.3	447.1	485.6	407.7
C [MHz]	366.8	345.2	367.5	334.3
$\mu_a/\mu_b/\mu_c$ [D]	1.8/1.2/0.2	0.3/0.4/0.0	1.8/1.1/0.3	0.4/0.5/0.1
ΔE [kJ/mol]	0.0	2.2	0.7	2.4

Table S7: Cartesian coordinates of the equilibrium structures of the four lowest conformers of FA-PA@PhF···He complex at the B3LYP-D3(BJ)/def2-TZVP level of theory.

	Conformer #1		
	X	Y	Z
C	-1.290891375411	1.583637838673	-1.148780738317
C	-0.088365582486	2.278935043469	-1.185266047788

Continued on next page

	X	Y	Z
C	0.617781383688	2.526037517832	-0.013323420055
C	0.120303111899	2.074822141168	1.204218204597
C	-1.079988856849	1.375681131528	1.259393124306
C	-1.759735685623	1.148822411862	0.077220651949
H	-1.854215443395	1.366644570838	-2.045772723672
H	0.302289873442	2.617858531053	-2.135747247482
H	1.557194085874	3.061088905380	-0.049671666907
H	0.672220640941	2.255506176605	2.117284319014
H	-1.481178005372	1.000650275416	2.190656989005
F	-2.923895304156	0.457363375569	0.119077323071
He	1.097267358219	-4.123634479582	-0.380961726885
C	4.184290279809	0.352254406010	-0.100597900416
H	5.152162186166	0.790584409080	-0.145169200698
C	3.093599003118	-0.142313522663	-0.049517898801
C	1.780246867465	-0.745981745587	-0.039192866160
O	1.155432210494	-0.915895479568	-1.076380492991
O	1.370983488643	-1.081071628539	1.160841943997
H	0.440270586284	-1.465912565215	1.120263521320
C	-1.697204360514	-2.228609239193	0.017906284163
H	-2.716206638765	-2.629205540277	0.020517456783
O	-1.060043743006	-2.088963663351	1.047597523041
O	-1.286786526405	-1.938156326888	-1.188813237690
H	-0.361387024424	-1.548465042866	-1.166003289203

Conformer #2			
	X	Y	Z
C	1.778575415022	-0.703748218903	-1.225886418558
C	0.625558136039	-1.448647106944	-1.446776500513
C	0.051570577704	-2.178025440990	-0.412096355724
C	0.630992985227	-2.163440284553	0.852110830116
C	1.783931741511	-1.424986214273	1.089047129669
C	2.334480301931	-0.710715075498	0.040646152080
H	2.238052117156	-0.119221866198	-2.010846862112
H	0.170979489810	-1.447621366003	-2.428729810026
H	-0.847343878328	-2.753242630460	-0.589632254899
H	0.180029453046	-2.721120028970	1.662511906794
H	2.246956195135	-1.387926236570	2.065487247556
F	3.451131854583	0.017938569453	0.263096536064
He	-3.690900064430	2.396518478301	1.169753064107
C	1.791082001485	3.023811790832	0.133009697597
H	2.683605817079	3.599032077000	0.190044253401
C	0.790005015880	2.367980975839	0.069743409675
C	-0.390356944739	1.538905497587	0.052342260372
O	-0.755257178273	0.941248842034	1.055585218725
O	-0.995118125173	1.504473296924	-1.112570107653
H	-1.783709423946	0.882532640860	-1.076962980220
C	-3.384502591591	-0.764100218450	-0.002496477955
H	-4.248766298638	-1.438994806933	0.002512255137

Continued on next page

	X	Y	Z
O	-3.060161115441	-0.150352140389	-1.003867437855
O	-2.789190088853	-0.723194742255	1.159796100061
H	-2.001036794507	-0.094884625041	1.135048621139

Conformer #3			
	X	Y	Z
C	0.774682375348	1.347334572060	-1.364158080363
C	-0.478593620343	1.936838516731	-1.240820507732
C	-0.924978131097	2.388413901104	-0.004046968265
C	-0.114551549218	2.252167669814	1.117731345826
C	1.141144384007	1.666938308074	1.012811386207
C	1.557524795334	1.228787805566	-0.231095213088
H	1.141472864862	0.976308582878	-2.311067435809
H	-1.109303628880	2.030813182956	-2.115038263236
H	-1.904738683758	2.837737627696	0.087289485297
H	-0.463360981857	2.591747156176	2.084153450592
H	1.785344387782	1.536573963932	1.871225903231
F	2.773069416808	0.640800239230	-0.341576432100
He	4.167757936357	-0.529430743092	2.183896436236
C	-4.286918853250	-0.100947049227	0.336792982102
H	-5.286161072513	0.249184863368	0.435839004936
C	-3.161393039415	-0.497261535111	0.222975107962
C	-1.801728304918	-0.978720272417	0.133896490228
O	-1.090475513269	-1.049880293324	1.126305683466
O	-1.448915446272	-1.320273614626	-1.081879120508
H	-0.482430074165	-1.606839252342	-1.102653772819
C	1.794592678579	-2.113728539038	-0.148958336613
H	2.847082612797	-2.407005192455	-0.218420817858
O	1.074855486211	-2.064987037985	-1.131527099120
O	1.442592331867	-1.836633350815	1.079595959969
H	0.483341397509	-1.541534983789	1.116501540554

Conformer #4			
	X	Y	Z
C	-1.429098334264	-0.980587001417	1.331233896674
C	-0.168621474260	-1.564831317101	1.269970621784
C	0.289854385978	-2.123317203900	0.082846166467
C	-0.513956406608	-2.097608955967	-1.051942006556
C	-1.776627325550	-1.520309366249	-1.006656481214
C	-2.208149536671	-0.974563826238	0.188493025126
H	-1.803320077350	-0.528737058598	2.239441240522
H	0.457983644024	-1.571896618401	2.152182625019
H	1.272014042407	-2.574682672751	0.039676320355
H	-0.154478726597	-2.519663202517	-1.981298071638
H	-2.416280601579	-1.478667918334	-1.877479243092
F	-3.432945225304	-0.405236807855	0.239257507330

Continued on next page

	X	Y	Z
He	5.883845400691	-1.046324804024	3.056546928036
C	-2.322473364630	2.764083263315	0.035146418310
H	-3.299147855728	3.181157399891	0.089315772461
C	-1.224931609422	2.286311451072	-0.023156267560
C	0.069917433183	1.662983481667	-0.147239268946
O	0.430693593905	1.164323772710	-1.204644120193
O	0.777931425301	1.693282424879	0.957893062135
H	1.640008797214	1.192460593317	0.835017806316
C	3.367028906313	-0.156612398675	-0.444602240372
H	4.314052070693	-0.699006805327	-0.550825408888
O	3.042269656080	0.362803357490	0.608491453587
O	2.678698461160	-0.155007653475	-1.555513233285
H	1.811167888021	0.344920322991	-1.436673588753

S4 Experimental parameters of the FA-PA@PhF complex and its isotopologues

Table S12: The experimental and theoretical spectroscopic constants for FA-PA@PhF complex.

	Experiment[a]		Theory[b]	
	Parent(0^+)	Parent(0^-)	Conformer #1	Conformer #2
A [MHz]	712.4895(28)	712.4881(28)	721.8	789.1
B [MHz]	528.07048(65)	528.07143(57)	535.8	473.8
C [MHz]	382.1440(28)	382.1438(28)	386.3	363.3
D_J [kHz]	0.1127(10)			
D_{JK} [kHz]	0.2357(62)			
d_1 [kHz]	-0.03593(61)			
ΔE_{01} [MHz]	50.8949(61)			
F_{bc} [MHz]	12.4488(13)			
F_{ca} [MHz]	18.191(26)			
$\mu_a/\mu_b/\mu_c$ [D][c]	intra-/intra-/inter-		1.8/1.2/0.2	0.3/0.5/0.1
σ [kHz][d]	4.7			
N[e]	203			
ΔE [kJ/mol]	–		0	1.5

[a] The experimental results were derived using the I^r representation of Watson's S reduction Hamiltonian, with standard deviations in parentheses expressed in units of the last two digits.

[b] The theoretical rotational constants were obtained at the B3LYP-D3(BJ)/def2-TZVPP level of theory, while the relative energy ΔE was obtained at the same level and further adjusted through the ZPE and BSSE corrections.

[c] Intra- denotes intrastate transition; Inter- denotes inter-state transition. In this work, we successfully observed the intrastate transitions.

[d] Root-mean-square deviation of the fit.

[e] Number of the lines in the fit.

Table S13: The experimental spectroscopic constants for FA-PA@PhF isotopologues, -CD and -OD species.

	-CD species		-OD species	
	FACD-PA@PhF(0^+)	FACD-PA@PhF(0^-)	FA-PAOD@PhF	FAOD-PA@PhF
A [MHz]	706.6014(66)[a]	706.6070(56)	709.59818(58)	709.42265(68)
B [MHz]	523.5069(64)	523.5068(61)	527.69467(28)	527.93760(33)
C [MHz]	378.086(10)	378.086(10)	379.16882(17)	378.90115(22)
D_J [kHz]	0.0895(20)		0.1052(22)	0.1036(26)
D_{JK} [kHz]	0.326(13)		0.277(14)	0.261(16)
d_1 [kHz]	-0.0243(11)		-0.0322(11)	-0.0305(14)
ΔE_{01} [MHz]	52.16(13)			
F_{bc} [MHz]	12.646(36)			
F_{ca} [MHz]	17.403(55)			
$\mu_a/\mu_b/\mu_c$ [D]	Y/Y/N[b]		Y/Y/N	Y/Y/N
σ [kHz]	5.3[c]		4.4	5.9
N	160[d]		75	81

[a] The experimental results were derived using the I^r representation of Watson's S reduction Hamiltonian, with standard deviations in parentheses expressed in units of the last two digits.
[b] Y denotes successfully observed in the spectrum; N denotes not found in the spectrum.
[c] Root-mean-square deviation of the fit.
[d] Number of the lines in the fit.

S5 Substitute structure of FA-PA@PhF

Based on the experimental parameters of FA-PA@PhF and FACD-PA@PhF, we utilized Kraitchman's equation[23] to precisely determine the position of the hydrogen atom within the CH group of formic acid, described as the r_s structure. This method circumvents the need for theoretical parameters and structures to obtain the atomic coordinates; however, the signs of the coordinates require theoretical validation. The calculations were performed using the KRA software available on the PROSPE website (`http://info.ifpan.edu.pl/~kisiel/prospe.htm`). Fig. S5 presents a schematic comparison of the substitution structure with the calculated structure, further confirming our assignment.

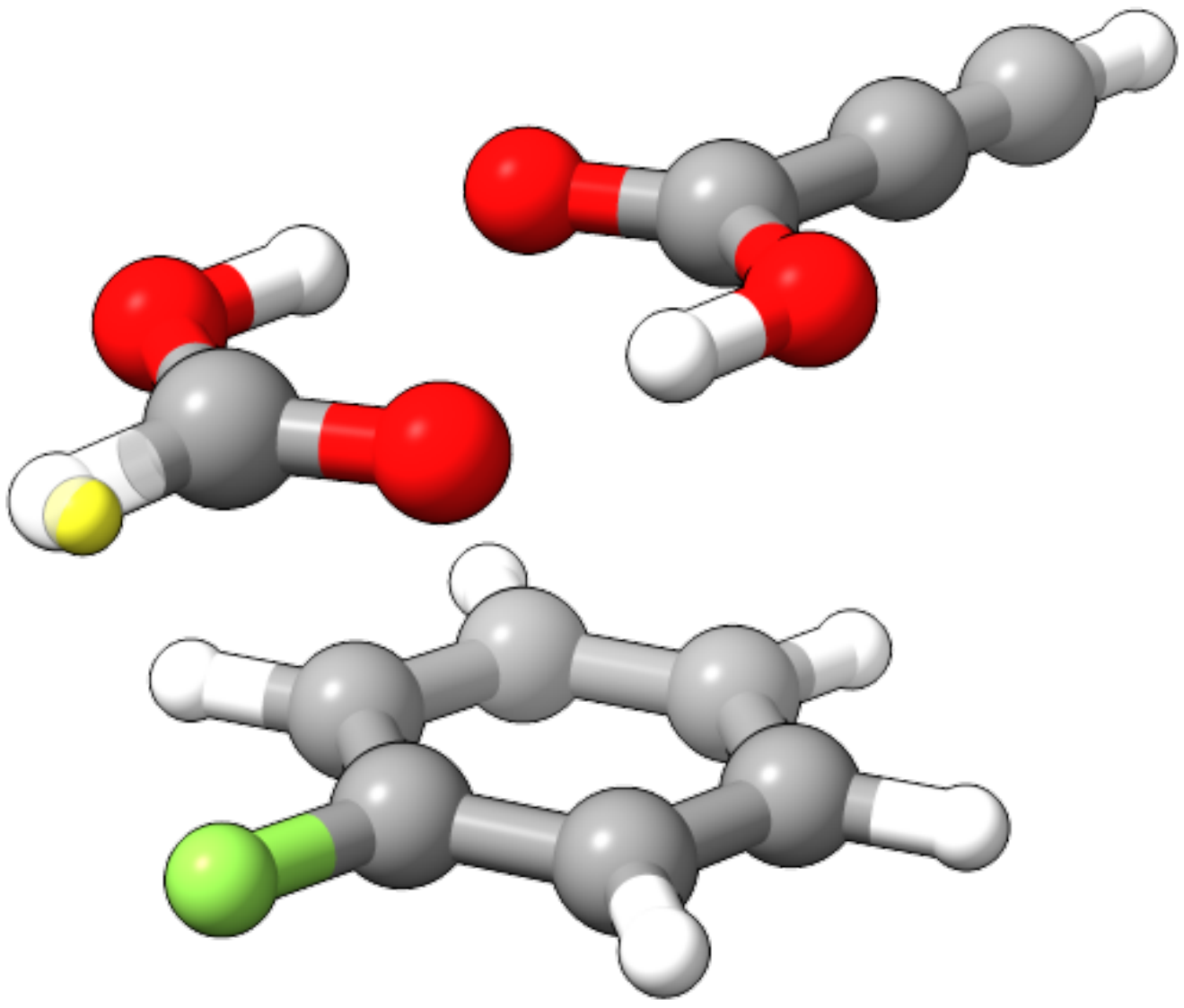

Figure S5: Substitute structure (r_s) of the FA-PA@PhF complex. The ball-and-stick model represents the calculated structure, superimposed with the r_s structural coordinates (yellow solid sphere).

Table S14: Coordinates of the H atom calculated using Kraitchman's equation

	X	Y	Z
		from 0^+	
H	2.84697 ± 0.00643	2.46374 ± 0.00786	0.17210 ± 0.11202
		from 0^-	
H	2.84774 ± 0.00635	2.46273 ± 0.00777	0.16452 ± 0.11590
		average	
H	2.84736±0.00452	2.46234±0.00553	0.16831±0.08059

S6 Experimental parameters of the FA-PA@PhF···He complex

Table S15: The experimental and theoretical spectroscopic constants for FA-PA@PhF···He complex.

	Experiment[a]		Theory[b]	
	Parent(0^+)	Parent(0^-)	Conformer #1	Conformer #2
A [MHz]	643.1463(68)	643.1496(70)	657.1	749.0
B [MHz]	522.4166(17)	522.4195(17)	531.3	447.1
C [MHz]	358.9677(69)	358.9678(69)	366.8	365.1
D_J [kHz]	0.1581(31)			
D_K [kHz]	0.465(22)			
D_{JK} [kHz]	-0.065(14)			
d_1 [kHz]	-0.0572(16)			
F_{bc} [MHz]	9.2729(86)			
F_{ca} [MHz]	16.905(57)			
ΔE_{01} [MHz]	47.579(38)			
$\mu_a/\mu_b/\mu_c$ [D][b]	intra-/intra-/inter-		1.8/1.2/0.2	0.3/0.4/0.0
σ [kHz][c]	4.7			
N[d]	229			
ΔE [kJ/mol]	–		0.0	2.2

[a] The experimental results were derived using the I^r representation of Watson's S reduction Hamiltonian, with standard deviations in parentheses expressed in units of the last two digits.

[b] The theoretical rotational constants were obtained at the B3LYP-D3(BJ)/def2-TZVPP level of theory, while the relative energy ΔE was obtained at the same level and further adjusted through the ZPE and BSSE corrections.

[c] Intra- denotes intrastate transition; Inter- denotes inter-state transition. In this work, we successfully observed the intrastate transitions.

[d] Root-mean-square deviation of the fit.

[e] Number of the lines in the fit.

S7 The calculation results of IGMH analysis

The Independent Gradient Model based on Hirshfeld partition (IGMH)[24] was employed to investigate the intermolecular interactions in the FA-PA@PhF and FA-PA@PhF$\cdots$He systems, as shown in Fig. S6. IGMH is an important graphical approach for visualizing both inter- and intrafragment interactions based on user-defined fragments, and it is implemented in the Multiwfn program[25]. In this study, we focus only on the interlayer interactions within the molecular clusters; for example, we define FA-PA as fragment 1, PhF as fragment 2, and He as fragment 3. Three-dimensional isosurfaces were generated at an isovalue of $\delta g = 0.0015$ a.u., where δg is a function of the electron density gradient that reflects the strength and nature of interactions. These isosurfaces are colored according to the sign$(\lambda_2)\rho$ function, which maps interaction types on a blue–green–red (BGR) scale within the range of $-0.03 < \text{sign}(\lambda_2)\rho < 0.03$. In addition to the 3D isosurfaces, two-dimensional scatter plots are also presented, colored using the same BGR scale. The scatter maps of sign$(\lambda_2)\rho$ versus δg^{inter} allow for straightforward identification of the correspondence between peaks in the scatter plots and features in the isosurfaces, providing a more intuitive interpretation of the spatial distribution and nature of interactions.

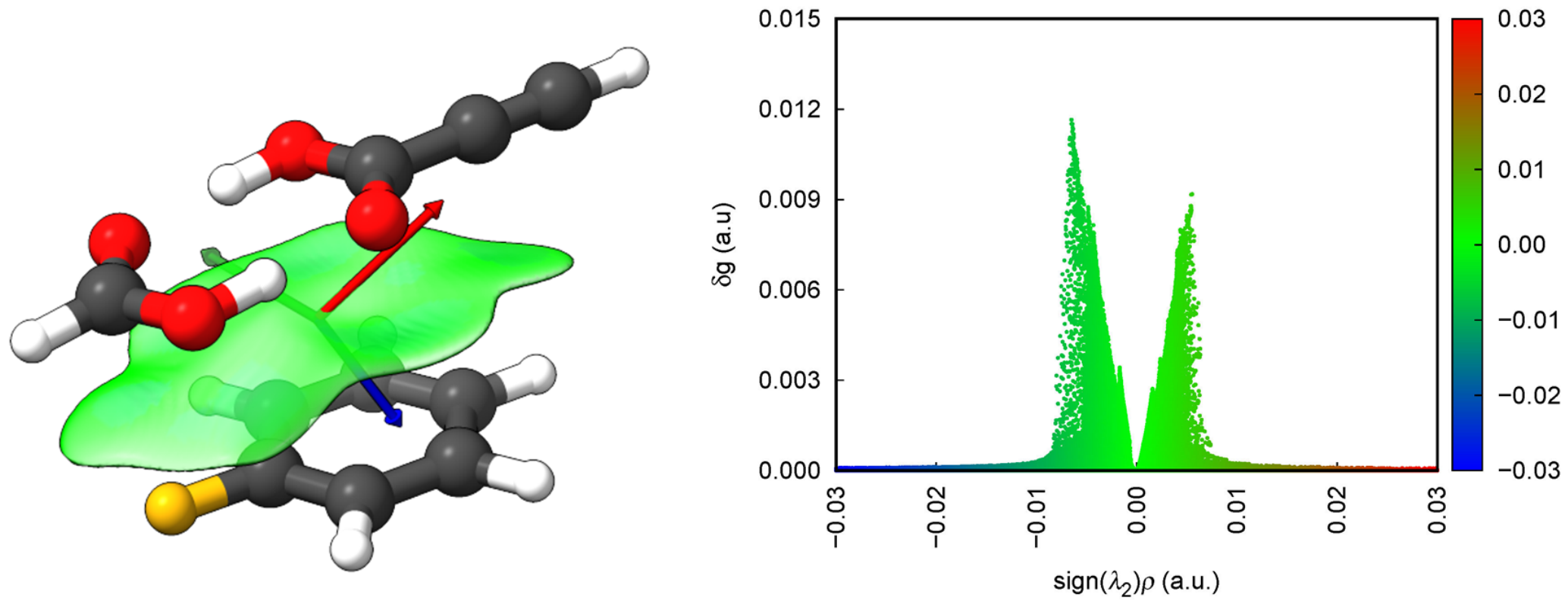

FA-PA@PhF

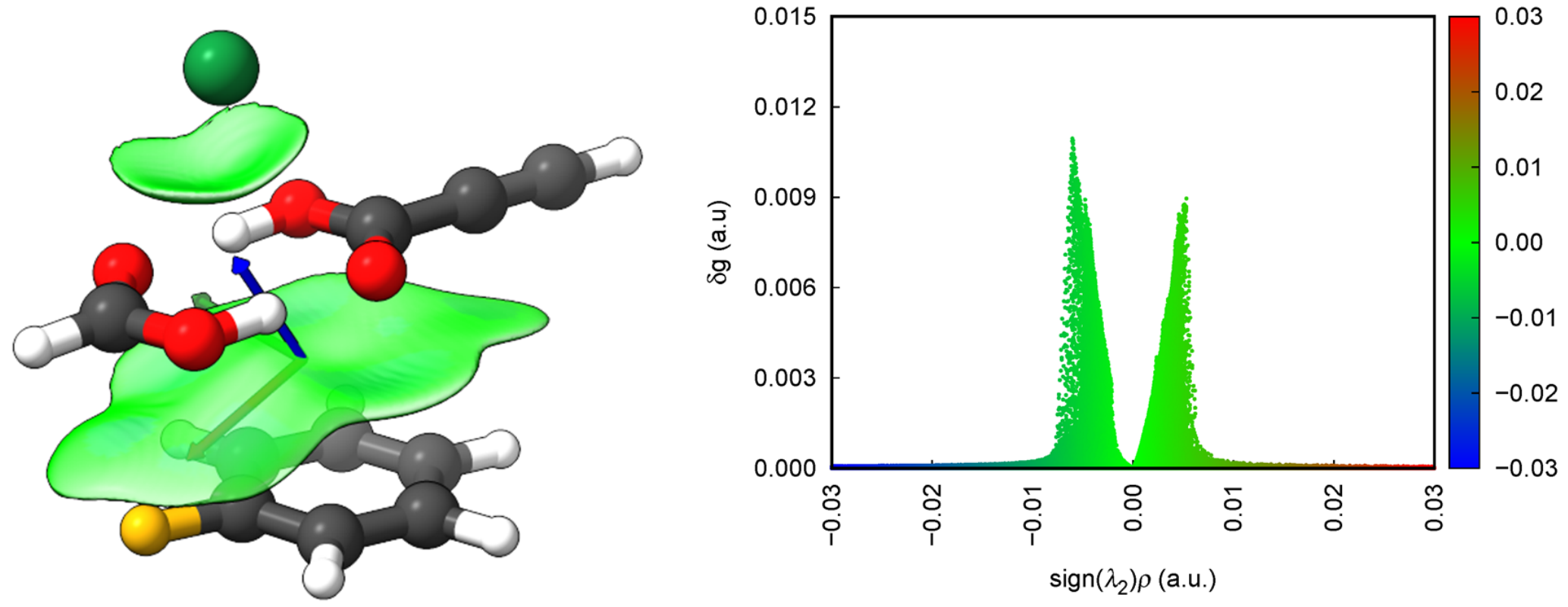

FA-PA@PhF···He

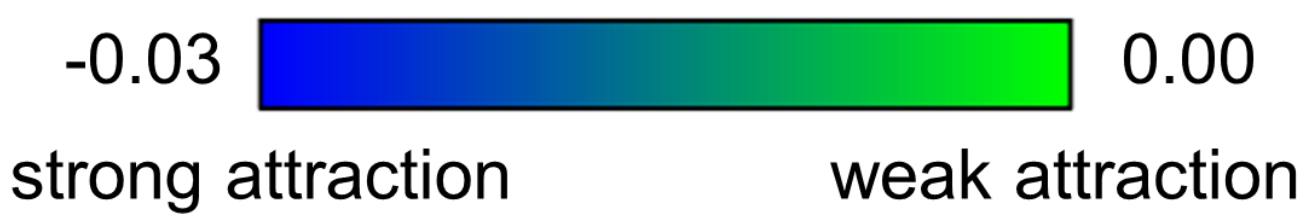

Figure S6: Three-dimensional isosurfaces and two-dimensional scatter plots obtained from IGMH analysis of FA-PA@PhF and FA-PA@PhF$\cdots$He. Isosurfaces were plotted at $\delta g = 0.0015$ a.u. and colored by sign$(\lambda_2)\rho$ within the range of -0.03 to 0.03 using a blue–green–red (BGR) scale. The 2D scatter plots show sign$(\lambda_2)\rho$ versus δg^{inter}, corresponding to the features observed in the isosurfaces.

S8 The calculation results of SAPT analysis

To rationalize the structural configurations of the observed clusters and interpret the experimental findings, we conducted a comprehensive two-body symmetry-adapted perturbation theory (SAPT) analysis[13] of the inter-fragment interactions in each cluster. This approach systematically delineates the magnitude of specific inter-fragment interactions, as summarized in the following sections.

Table S16: SAPT2+δMP2/aug-cc-pVTZ//B3LYP-D3(BJ)/def2-TZVPP interaction energies of the formic acid dimer (FA-FA) and formic acid-propiolic acid dimer (FA-PA) with the fluorobenzene (PhF) for the FA-FA@PhF and two conformers of the FA-PA@PhF complex. All values are given in kJ/mol.

	FA-FA@PhF	FA-PA@PhF	
		Conformer #1	Conformer #2
Exchange	28.6	33.9	33.4
Electrostatic	-14.7	-17.3	-15.4
Induction	-2.3	-2.8	-2.7
Dispersion	-33.4	-39.5	-38.1
Total	-21.9	-25.7	-22.8

Table S17: SAPT2+δMP2/aug-cc-pVTZ//B3LYP-D3(BJ)/def2-TZVPP interaction energies of FA-PA@PhF$\cdots$He complex. All values are given in kJ/mol.

Partitioning Scheme 1: He + FA-PA@PhF[a]		
	Conformer #1	Conformer #2
Exchange	1.21	1.10
Electrostatic	-0.23	-0.21
Induction	-0.06	-0.05
Dispersion	-2.00	-1.84
Total	-1.08	-0.99
Partitioning Scheme 2: PhF + FA-PA$\cdots$He [b]		
	Conformer #1	Conformer #2
Exchange	33.9	32.2
Electrostatic	-16.4	-13.9
Induction	-2.6	-2.4
Dispersion	-38.5	-36.5
Total	-23.6	-20.6

[a] Partitioning Scheme 1: He is defined as Fragment 1, and FA-PA@PhF as Fragment 2.

[b] Partitioning Scheme 2: PhF is defined as Fragment 1, and the remaining FA-PA$\cdots$He is defined as Fragment 2.

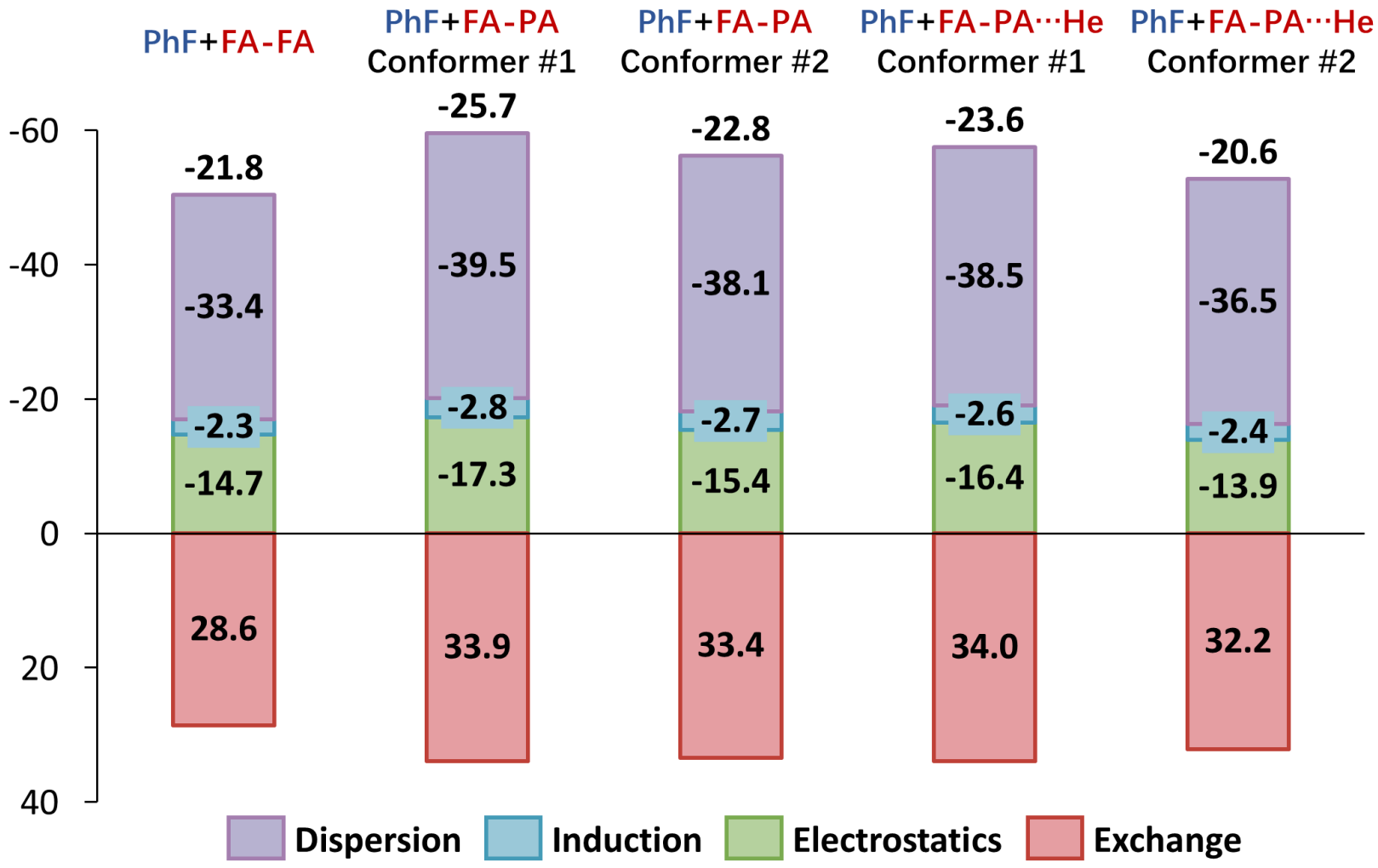

Figure S7: Two-body intermolecular interaction energies calculated by SAPT2+δMP2/aug-cc-pVTZ//B3LYP-D3(BJ)/def2-TZVPP, with the blue and red text denoting different fragments. All values in the figure are given in kJ/mol.

S9 Comparison of experimental tunneling splitting energy and tunneling period

The tunneling splitting energy ΔE_{01} refers to the energy gap between two levels into which the ground vibrational state is split due to tunneling. Based on the two-state proton transfer-rotation coupled Hamiltonian described in the Methods section, the tunneling splitting energy ΔE_{01} can be determined experimentally. This energy is directly related to the tunneling period τ, which describes the wavepacket motion between the potential wells beneath the energy barrier, and is given by $\tau = \frac{h}{\Delta E_{01}}$[26,27]. Here, we compare the experimental tunneling splitting energy and their corresponding tunneling periods for different systems, including FA-FA, FA-PA, FA-FA@PhF, FA-PA@PhF, and FA-PA@PhF$\cdots$He, as shown in Fig. S18.

Table S18: Comparison of experimental splitting energies (ΔE_{01}) and tunneling periods (τ).

Species	Splitting energies (ΔE_{01}) [MHz]	Tunneling periods (τ) [ns]
FA-FA	341.0(28)[28]	2.9
FA-PA	291.428(5)[29]	3.4
FA-FA@PhF	267.608(1)[20]	3.7
FA-PA@PhF	50.8949(61)	19.6
FACD-PA@PhF	52.16(13)	19.2
FA-PA@PhF$\cdots$He	47.579(18)	21.0

S10 Instanton theory calculations

S10.1 Theoretical details

Density functional theory (DFT) calculations were performed using the Gaussian16 package[30] at the B3LYP-D3(BJ)/def2-TZVP level. The instanton calculations were performed using the instanton program developed by Prof. Jeremy Richardson[31,32], in combination with Gaussian16 software for B3LYP-D3(BJ) functional calculations. To reduce the computational cost of the electronic structure calculation for a total number of more than 20 atoms, we employed a Gaussian process regression (GPR) assisted approach for instanton optimizations[33,34].

The initial data set contains around 10 potential and gradient data points, and 3 Hessian data. We start the iteration process from GPR instanton optimization with a relatively small number of beads at relatively high temperature. The geometries of two ends along the instanton trajectory are fixed at the two permutationally equivalent minimums, with their center of mass aligned on top of each other. They are not allowed to rotate about the center of mass during the optimization process. After one round of GPR optimization, we select 10-12 roughly evenly spaced geometries along the instanton, and compute the potential energy and gradients on these geometries to generate new *ab initio* data. This process is iterated, and as we iterate, we increase the number of beads and decrease the temperature. To prevent the dataset becoming unnecessarily large and inefficient, we only keep data from the most recent one iteration, and the number of Hessian data is limited to 3 each time we decrease the temperature. At the lowest temperature 7 K (the optimization employed at 5 K is to confirm the convergence of instanton at 7 K), we further reduce the optimization limit of GPR process to obtain more accurate instanton. When the instanton geometry barely changes during iterations at 7 K, we estimate the *ab initio* gradients on all the beads to check if the instanton optimization has fully converged as if the optimisation was done without GPR. The whole *ab initio* gradient is converged to below 5e-3 hartree/Angstrom for the instanton pathway of both systems. The GPR iteration process is shown in Fig. S9. Instanton tunneling splitting is given by

$$\Delta E_{\text{splitting}} = \frac{2\hbar}{\Phi}\sqrt{\frac{S}{2\pi\hbar}}\mathrm{e}^{-S/\hbar}, \tag{11}$$

where S is the Euclidean action of the instanton and Φ is a measure of the fluctuations around the instanton. The action S is corrected at the CCSD(T)-F12-RI/cc-pVDZ-F12 level[35,16,36], computed with ORCA[11] using a dual-level approach[37]. Finally, we compute the *ab initio* Hessian along the instanton pathway to obtain the accurate tunneling splitting. The convergence of $S/\hbar$ with respect to the number of beads (N) and the imaginary time length ($\beta\hbar$) are presented in Table S20. For both systems, $S/\hbar$ are converged with the computational setup of N = 2000 and $\beta\hbar$ with β value corresponding to a temperature of 7 K.

Additionally, the instanton pathways of the Conformer #2 of FA-PA@PhF and FA-PA@PhF$\cdots$He clusters have been optimized at exactly the same computational level as the Conformer #1. The whole *ab initio* gradients of both instantons are converged to below 5e-3 hartree/Angstrom with the computational setup of N = 2000 and a temperature of 7 K. It is confirmed that for both FA-PA@PhF and FA-PA@PhF$\cdots$He clusters, Conformer #2 would undergo a longer tunneling pathlength than Conformer #1 and therefore the proton tunneling process is less likely to occur in the Conformer #2. The results of the squared mass-weighted tunneling path length (SMWTPL) for each atom, based on instanton theory calculations, are summarized in Fig. S10 - Fig. S13. The contribution of each atom to the tunneling process is then quantified by computing the ratio between the SMWTPL of each atom and the total SMWTPL across all atoms. We also present the tunneling path length without considering atomic mass, that is, the pure distance that each atom moves during the tunneling process.

Table S19: Tunneling splittings (ΔE), instanton actions ($S/\hbar$), and fluctuation parameters (Φ) for the studied clusters.

**System**	ΔE_{expt} (MHz)	ΔE_{calc} (MHz)	$S/\hbar$	Φ (a.u.)
FA-FA	341.0(28)	247.97	15.76	12.02
FA-PA	291.428(5)	207.13	16.18	9.54
FA-PA@PhF conformer#1	50.8949(61)	68.82	19.06	1.76
FA-PA@PhF conformer#2	Not observed.	20.16	20.26	1.86
FA-PA@PhF···He conformer#1	47.579(18)	59.74	18.94	2.27
FA-PA@PhF···He conformer#2	Not observed.	12.71	20.50	2.14

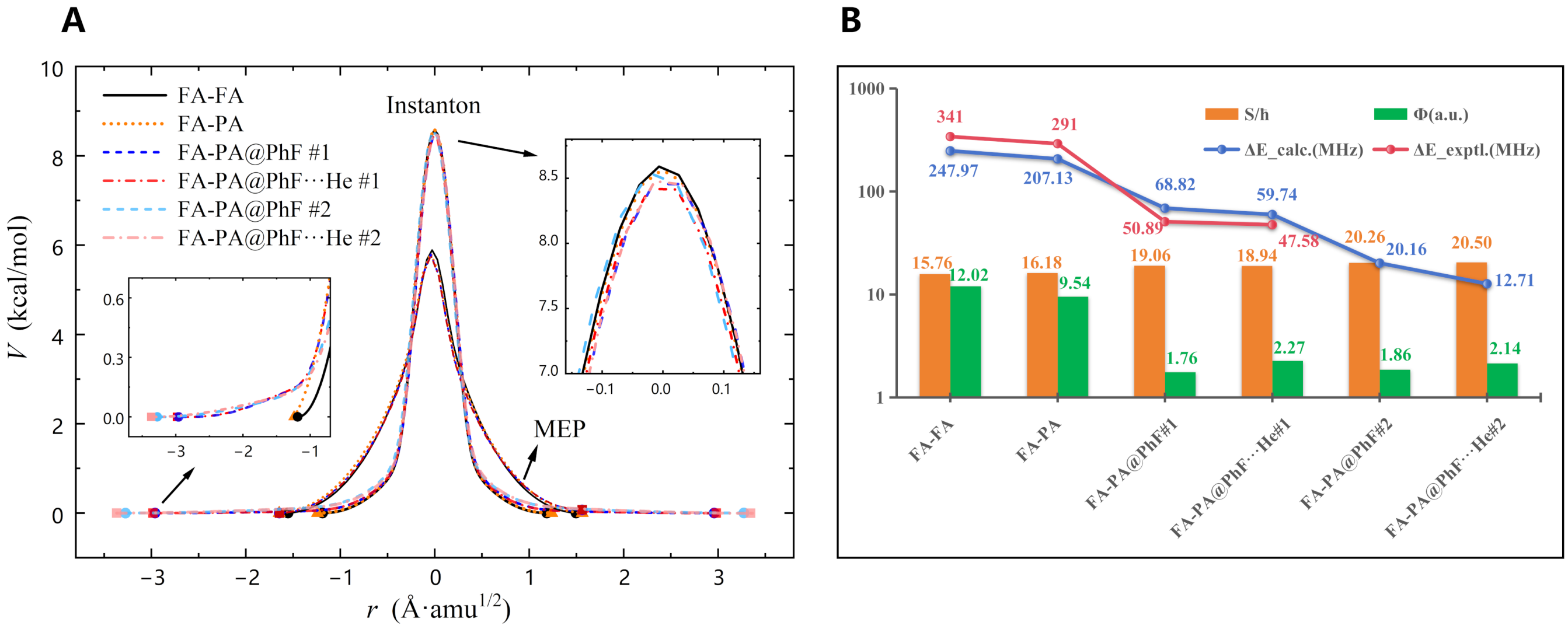

Figure S8: Comparison of tunneling paths and splittings. (A) Instanton tunneling paths (ITPs) and minimum energy paths (MEPs) of FA-FA, FA–PA, FA–PA@PhF, and FA–PA@PhF···He, together with (B) the comparison of tunneling splittings (ΔE_{01}) between experiment and instanton theory, as well as the instanton actions ($S/\hbar$) and instanton fluctuations (Φ). The full-dimensional instanton optimizations were performed at the B3LYP-D3(BJ)/def2-TZVP level, with single-point energy corrections computed at the CCSD(T)-F12-RI/cc-pVDZ-F12 level.

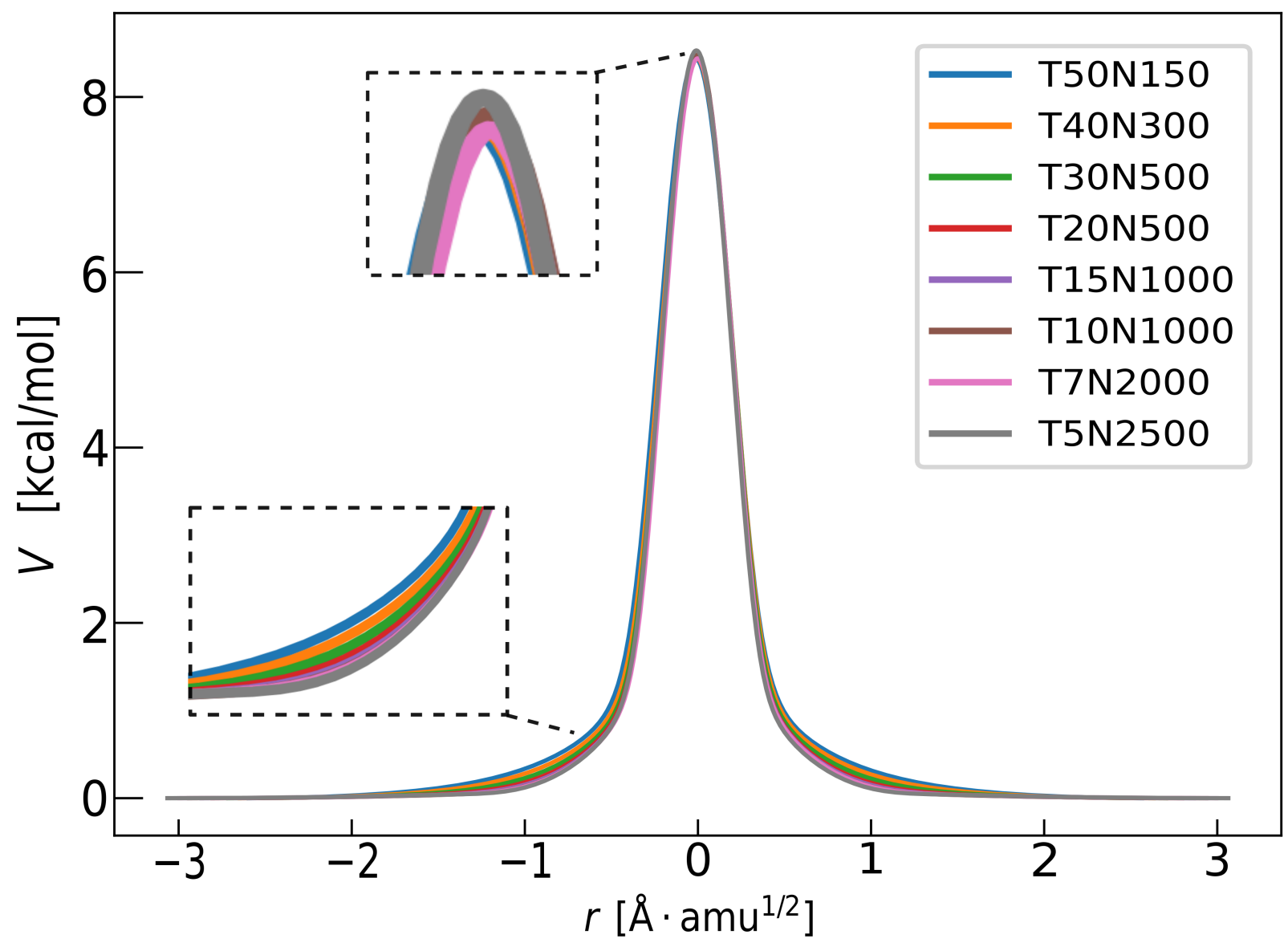

Figure S9: Plot of the GPR-optimized instanton trajectories of the FA-PA@PhF$\cdots$He Conformer #1 after different iterations, optmized at the B3LYP-D3(BJ)/def2-TZVP level.

Table S20: Convergence of S, optimized at the B3LYP-D3(BJ)/def2-TZVP level, with respect to the number of beads and the imaginary time length ($\beta\hbar$). The temperatures in the table correspond to β in the imaginary time length.

	$S/\hbar$	50K	40K	30K	20K	15K	10K	7K	5K
FA-PA@PhF	150 beads	20.96							
Conformer #1	300 beads		19.21						
	500 beads			17.46	15.80				
	1000 beads						14.48		
	2000 beads							14.37	
FA-PA@PhF	300 beads	24.78	22.33						
Conformer #2	500 beads		20.03	17.92					
	1000 beads				17.93	17.07	16.45		
	2000 beads						16.46	16.26	
FA-PA@PhF$\cdots$He	150 beads	21.15							
Conformer #1	300 beads	21.23	19.33	17.50					
	500 beads			17.53	15.82	15.02			
	1000 beads					15.00	14.35		
	2000 beads							14.22	
	2500 beads								14.16
FA-PA@PhF$\cdots$He	150 beads	25.41							
Conformer #2	300 beads	25.52	22.94	20.48					
	500 beads			20.48					
	1000 beads				18.33	17.39			
	2000 beads						16.85	16.97	

Atom	Movement (Å)	Length (Å·amu$^{1/2}$)	Devotion (%)
1 (C)	0.2902	1.0054	1.20
2 (H)	0.3313	0.3326	0.10
3 (C)	0.2757	0.9551	1.60
4 (C)	0.2559	0.8866	2.60
5 (O)	0.4253	1.7011	8.30
6 (O)	0.4251	1.7002	8.30
7 (H)	0.9245	0.9281	21.70
8 (C)	0.1958	0.6782	3.10
9 (H)	0.2426	0.2435	0.40
10 (O)	0.3745	1.4981	8.40
11 (O)	0.3737	1.4947	8.40
12 (H)	0.9244	0.9280	21.70
13 (C)	0.3784	1.3110	2.10
14 (C)	0.4493	1.5563	2.80
15 (C)	0.3500	1.2123	1.60
16 (C)	0.4506	1.5611	2.80
17 (C)	0.3789	1.3125	2.10
18 (C)	0.0515	0.1783	0.00
19 (H)	0.6466	0.6492	0.50
20 (H)	0.7273	0.7301	0.60
21 (H)	0.4828	0.4847	0.20
22 (O)	0.7295	0.7323	0.60
23 (C)	0.6465	0.6490	0.50
24 (F)	0.1458	0.6357	0.40

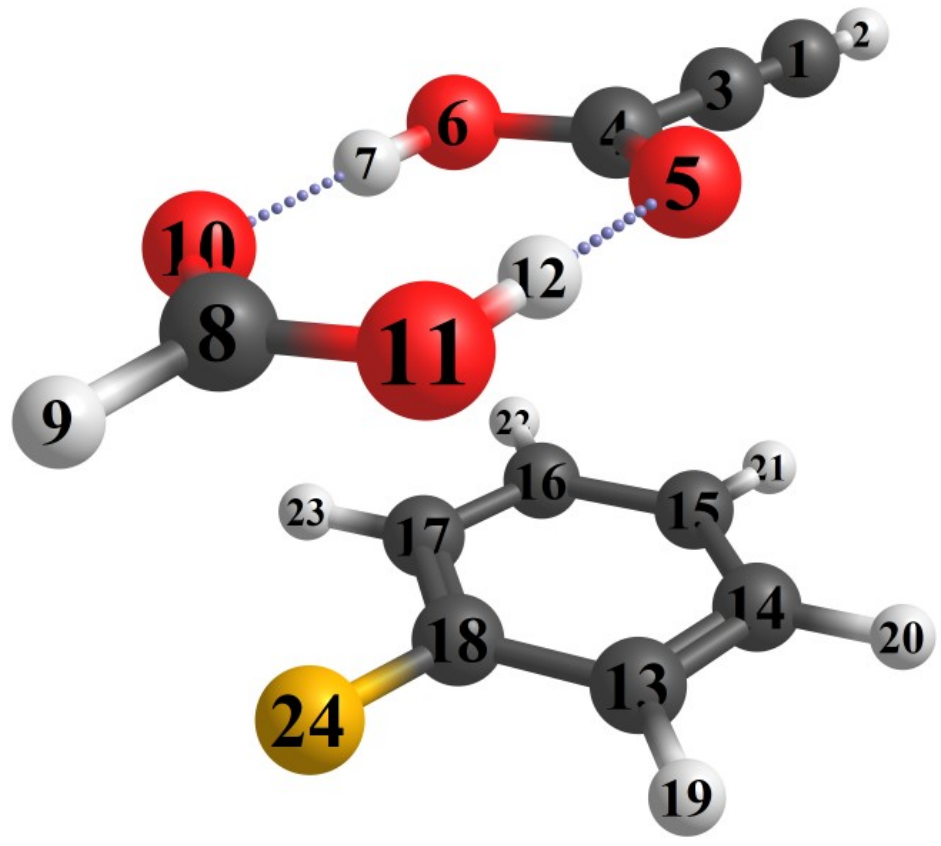

Figure S10: The results of SMWTPL for each atom in FA-PA@PhF Conformer #1.

Atom	Movement (Å)	Length (Å·amu$^{1/2}$)	Devotion (%)
1 (C)	0.3012	1.0435	1.20
2 (H)	0.3387	0.3400	0.10
3 (C)	0.2895	1.0030	1.70
4 (C)	0.2724	0.9435	2.70
5 (O)	0.4293	1.7173	8.40
6 (O)	0.4293	1.7173	8.40
7 (H)	0.9175	0.9211	21.60
8 (C)	0.1858	0.6437	3.10
9 (H)	0.2304	0.2313	0.40
10 (O)	0.3566	1.4262	8.00
11 (O)	0.3566	1.4262	8.00
12 (H)	0.9175	0.9211	21.60
13 (C)	0.3703	1.2829	2.00
14 (C)	0.4480	1.5519	2.80
15 (C)	0.3616	1.2525	1.70
16 (C)	0.4480	1.5519	2.80
17 (C)	0.3703	1.2829	2.00
18 (C)	0.0536	0.1856	0.10
19 (H)	0.6280	0.6304	0.50
20 (H)	0.7172	0.7200	0.60
21 (H)	0.4961	0.4980	0.30
22 (O)	0.7172	0.7200	0.60
23 (C)	0.6280	0.6304	0.50
24 (F)	0.1414	0.6163	0.40
25 (He)	0.3888	0.7778	0.50

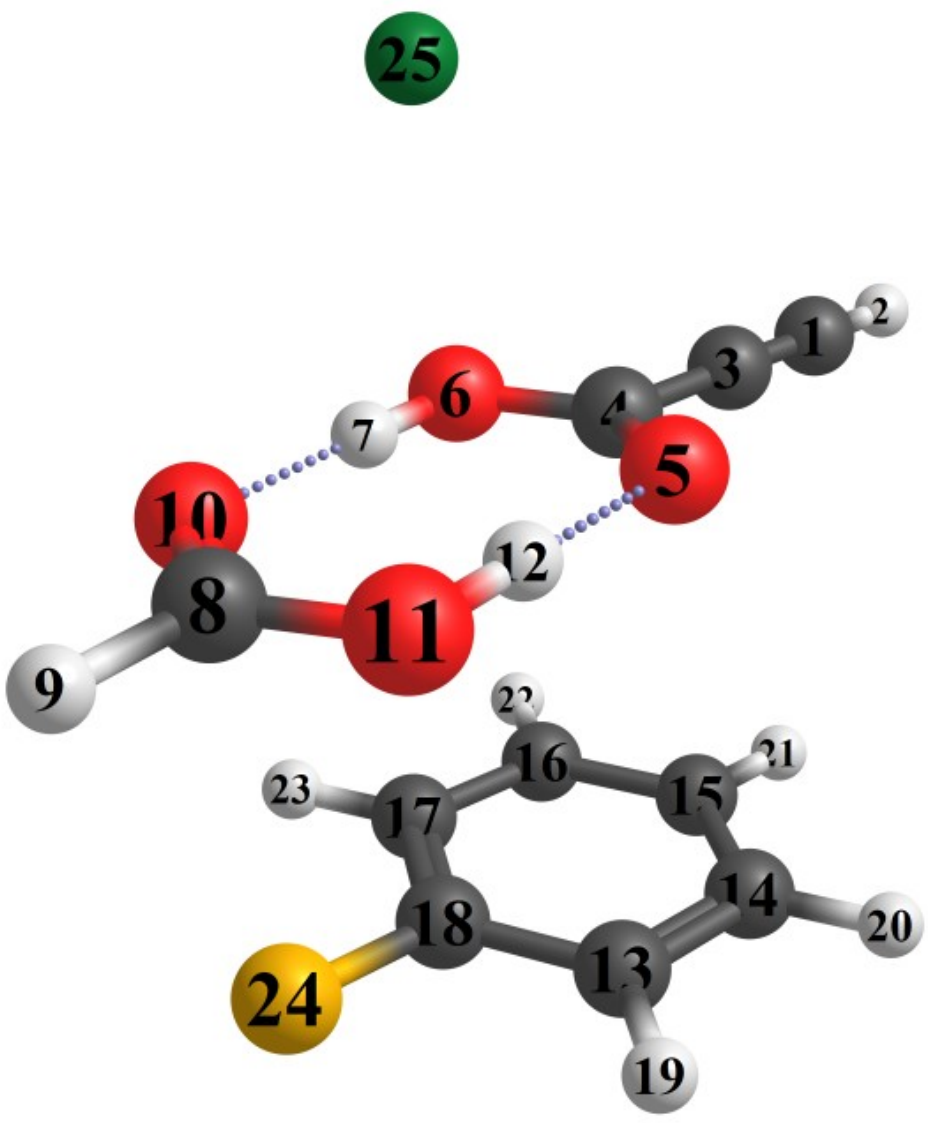

Figure S11: The results of SMWTPL for each atom in FA-PA@PhF$\cdots$He Conformer #1.

Atom	Movement (Å)	Length (Å·amu$^{1/2}$)	Devotion (%)
1 (C)	0.1256	0.4352	0.40
2 (H)	0.1365	0.1370	0.10
3 (C)	0.1718	0.5952	1.00
4 (C)	0.2320	0.8036	2.40
5 (O)	0.5139	2.0554	10.00
6 (O)	0.5130	2.0516	10.00
7 (H)	1.0263	1.0303	19.80
8 (C)	0.2911	1.0084	3.10
9 (H)	0.3638	0.3653	0.40
10 (O)	0.4686	1.8741	8.10
11 (O)	0.4703	1.8810	8.10
12 (H)	1.0259	1.0299	19.70
13 (C)	0.4500	1.5587	2.60
14 (C)	0.5118	1.7730	3.30
15 (C)	0.3658	1.2673	1.60
16 (C)	0.5104	1.7679	3.30
17 (C)	0.4492	1.5559	2.60
18 (C)	0.0935	0.3239	0.20
19 (H)	0.7533	0.7562	0.60
20 (H)	0.8267	0.8299	0.70
21 (H)	0.4830	0.4849	0.20
22 (H)	0.8244	0.8277	0.70
23 (H)	0.7529	0.7559	0.60
24 (F)	0.0854	0.3724	0.10

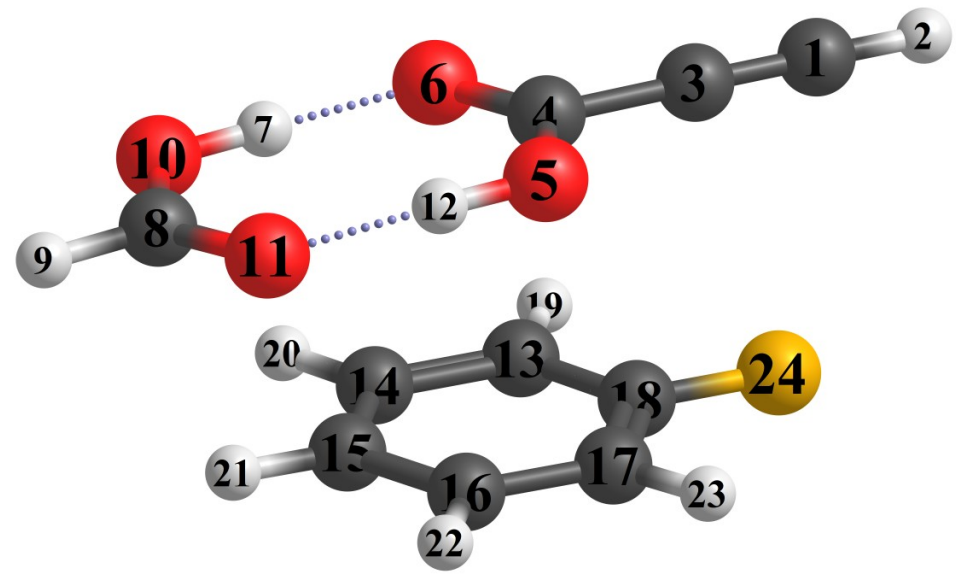

Figure S12: The results of SMWTPL for each atom in FA-PA@PhF Conformer #2.

Atom	Movement (Å)	Length (Å·amu$^{1/2}$)	Devotion (%)
1 (C)	0.1209	0.4189	0.40
2 (H)	0.128	0.1285	0.10
3 (C)	0.1752	0.6068	1.00
4 (C)	0.2500	0.8660	2.50
5 (O)	0.5138	2.0550	10.00
6 (O)	0.5141	2.0561	10.00
7 (H)	1.0278	1.0318	19.80
8 (C)	0.3318	1.1496	3.30
9 (H)	0.4098	0.4114	0.40
10 (O)	0.4728	1.8907	7.80
11 (O)	0.4721	1.8882	7.80
12 (H)	1.0269	1.0309	19.80
13 (C)	0.4371	1.5141	2.40
14 (C)	0.5103	1.7678	3.20
15 (C)	0.3937	1.3640	1.80
16 (C)	0.5117	1.7724	3.20
17 (C)	0.4364	1.5116	2.40
18 (C)	0.0897	0.3108	0.20
19 (H)	0.7252	0.7280	0.50
20 (H)	0.8103	0.8135	0.70
21 (H)	0.5212	0.5233	0.30
22 (H)	0.8127	0.8159	0.70
23 (H)	0.7234	0.7262	0.50
24 (F)	0.0830	0.3619	0.20
25 (He)	0.5451	1.0905	1.10

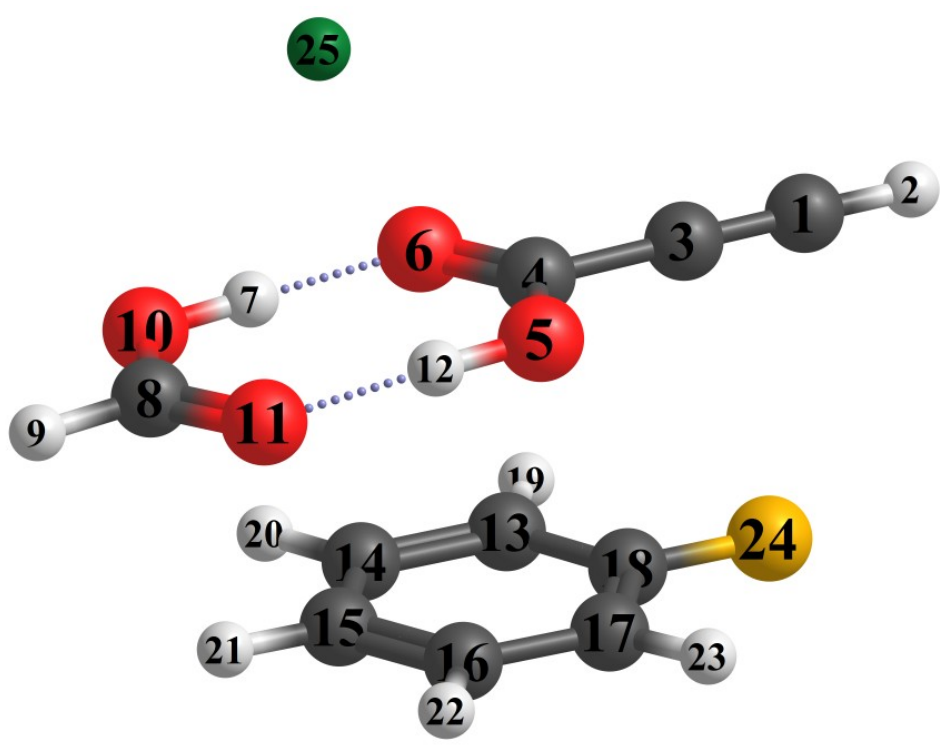

Figure S13: The results of SMWTPL for each atom in FA-PA@PhF$\cdots$He Conformer #2.

S10.2 Reorganization energy estimation from the intrinsic reaction coordinate optimization

The theoretical reorganization energies of the PhF molecule and the He atom were obtained from *ab initio* calculations at the B3LYP-D3(BJ)/def2-TZVP level. We performed intrinsic reaction coordinate (IRC) calculations based on the classical transition-state (TS) structures of the FA-FA@PhF, FA-PA@PhF, and FA-PA@PhF···He clusters, which are shown in Fig S14, Fig S15, and Fig. S16, respectively. Owing to the lack of reorganization of the environmental molecules, the two ends of the IRC path have different single-point energies; therefore, the energy difference (ΔE) between the two ends is taken as the theoretical reorganization energy. Compared with the experimentally determined reorganization energies reported in the manuscript [PhF: 0.14 kJ/mol for FA-FA@PhF and 0.43 kJ/mol for FA-PA@PhF; He: 0.06 kJ/mol for FA-PA@PhF···He], the theoretically calculated values are of the same order of magnitude, supporting the reliability of our computational approach.

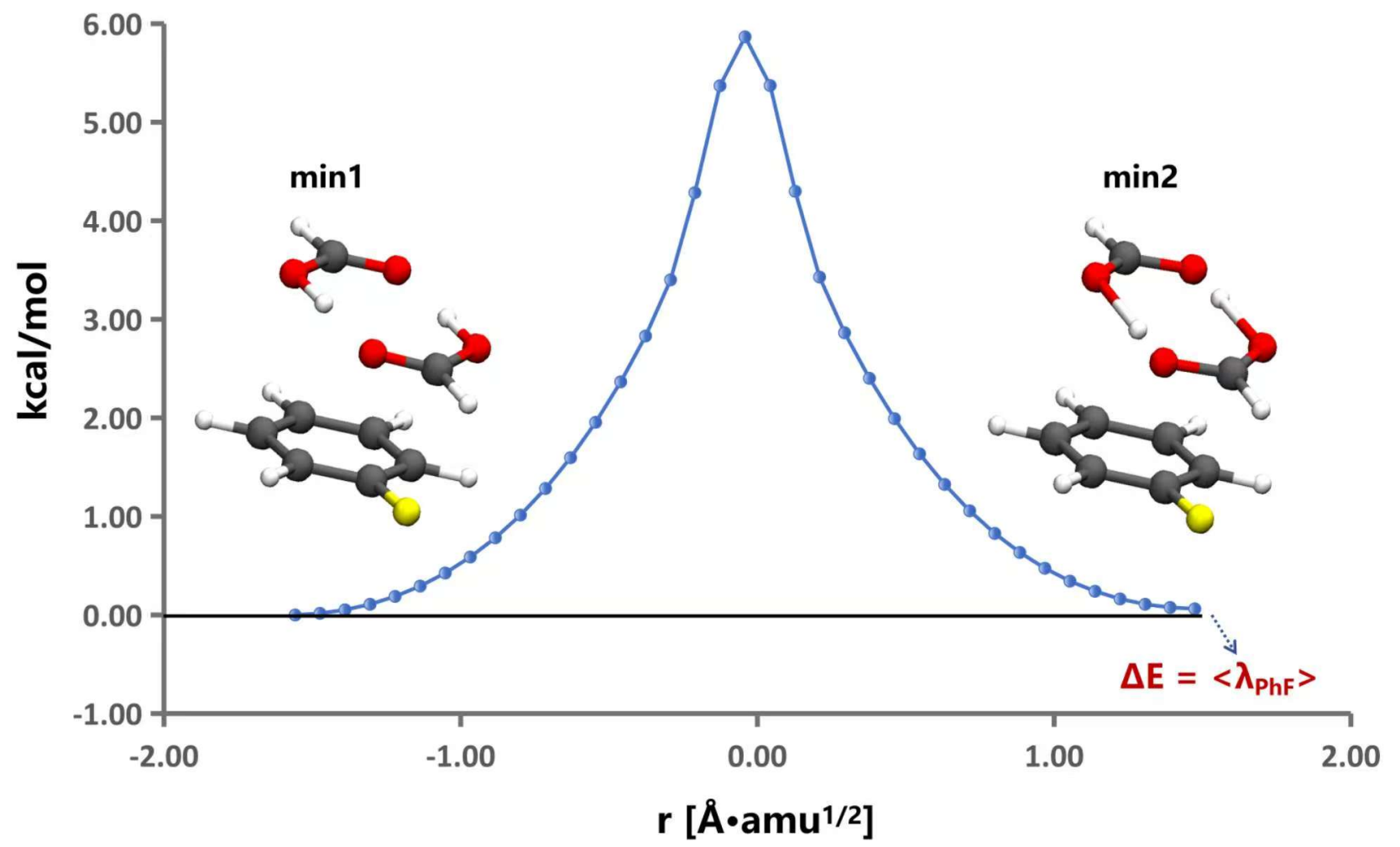

Figure S14: Minimum energy pathway of the FA-FA@PhF complex performed at the B3LYP-D3(BJ)/def2-TZVP level, showing the bare double proton transfer action without the reorganization of PhF.

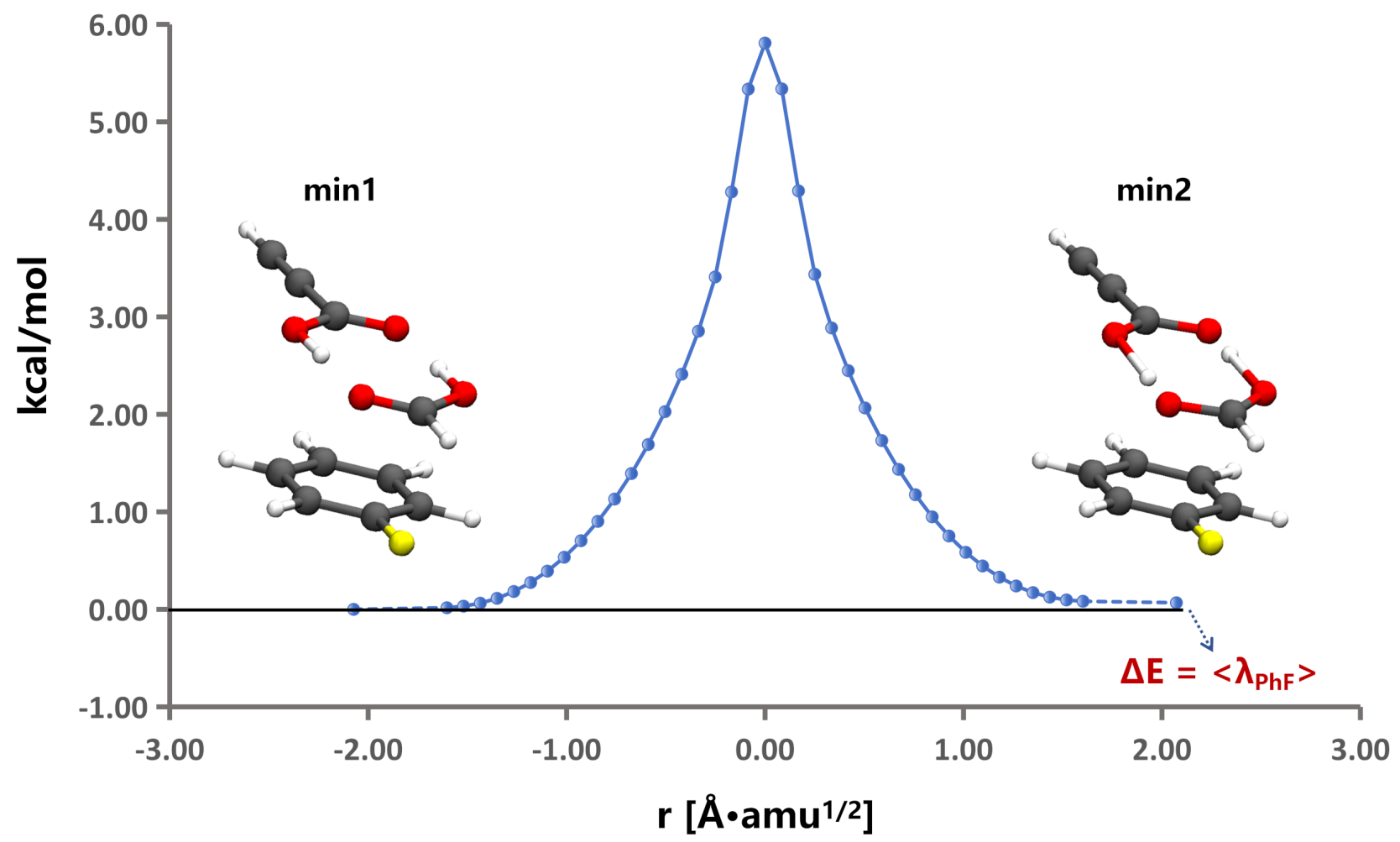

Figure S15: Minimum energy pathway of the FA-PA@PhF complex performed at the B3LYP-D3(BJ)/def2-TZVP level, showing the bare double proton transfer action without the reorganization of PhF.

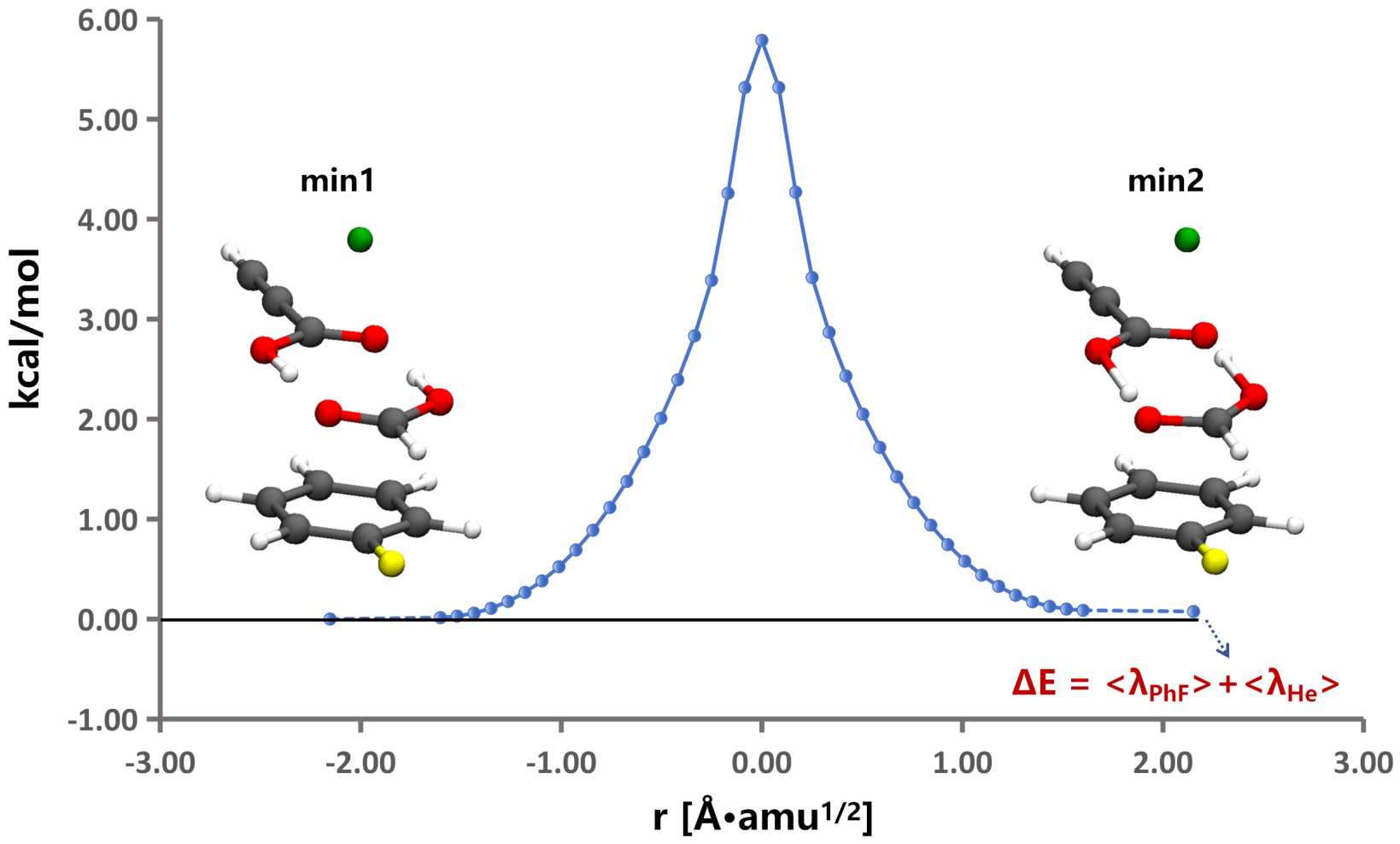

Figure S16: Minimum energy pathway of the FA-PA@PhF$\cdots$He complex performed at the B3LYP-D3(BJ)/def2-TZVP level, showing the bare double proton transfer action without the reorganization of PhF and He.

S11 Rotational transitions of the FA-PA@PhF complex and its isotopologues

Table S21: Measured frequencies of the rotational transitions of FA-PA@PhF.

J'	Ka'	Kc'	v'	J	Ka	Kc	v	obs/MHz	obs-cal/MHz
7	0	7	1	6	1	6	1	5543.7973	-0.0011
7	1	7	1	6	1	6	1	5544.0130	-0.0016
7	0	7	0	6	1	6	0	5544.2415	-0.0006
7	1	7	0	6	1	6	0	5544.4559	-0.0021
7	0	7	1	6	0	6	1	5544.6554	-0.0012
7	1	7	1	6	0	6	1	5544.8717	-0.0012
7	0	7	0	6	0	6	0	5545.0979	-0.0025
7	1	7	0	6	0	6	0	5545.3073	-0.0090
8	0	8	1	7	1	7	1	6304.3080	0.0163
8	1	8	1	7	1	7	1	6304.3080	0.0163
8	0	8	1	7	0	7	1	6304.4879	-0.0201
8	1	8	1	7	0	7	1	6304.4879	-0.0201
8	0	8	0	7	1	7	0	6304.7486	0.0171
8	1	8	0	7	1	7	0	6304.7486	0.0171
8	0	8	0	7	0	7	0	6304.9274	-0.0201
8	1	8	0	7	0	7	0	6304.9274	-0.0201
9	0	9	1	8	1	8	1	7064.4928	-0.0017
9	1	9	1	8	1	8	1	7064.4928	-0.0017
9	0	9	1	8	0	8	1	7064.4928	-0.0017
9	1	9	1	8	0	8	1	7064.4928	-0.0017
9	0	9	0	8	1	8	0	7064.9242	-0.0060
9	1	9	0	8	1	8	0	7064.9242	-0.0060
9	0	9	0	8	0	8	0	7064.9242	-0.0060
9	1	9	0	8	0	8	0	7064.9242	-0.0060
0	0	10	1	9	1	9	1	7824.5957	-0.0042
0	1	10	1	9	1	9	1	7824.5957	-0.0042
0	0	10	1	9	0	9	1	7824.5957	-0.0042
0	1	10	1	9	0	9	1	7824.5957	-0.0042
0	0	10	0	9	1	9	0	7825.0275	-0.0043
0	1	10	0	9	1	9	0	7825.0275	-0.0043
0	0	10	0	9	0	9	0	7825.0275	-0.0043
0	1	10	0	9	0	9	0	7825.0275	-0.0043
3	0	3	1	2	0	2	1	2530.5646	-0.0006
3	0	3	0	2	0	2	0	2531.0796	-0.0030
4	0	4	1	3	0	3	1	3277.2823	-0.0010
4	0	4	0	3	0	3	0	3277.7674	0.0001
5	0	5	1	4	0	4	1	4028.6648	-0.0002
5	0	5	0	4	0	4	0	4029.1242	-0.0009
6	0	6	1	5	0	5	1	4785.5431	-0.0009
6	0	6	0	5	0	5	0	4785.9930	-0.0004
3	1	2	1	2	1	1	1	2900.7033	0.0013
3	1	2	0	2	1	1	0	2901.9623	0.0021
4	1	4	1	3	1	3	1	3252.1083	0.0076
4	1	4	0	3	1	3	0	3252.5268	-0.0026

Continued on next page

J'	Ka'	Kc'	v'	J	Ka	Kc	v	obs/MHz	obs-cal/MHz
4	1	3	1	3	1	2	1	3758.4171	0.0002
4	1	3	0	3	1	2	0	3759.8727	0.0003
5	1	5	1	4	1	4	1	4020.3666	0.0005
5	1	5	0	4	1	4	0	4020.8104	0.0000
5	1	4	1	4	1	3	1	4530.6188	-0.0003
5	1	4	0	4	1	3	0	4532.0754	-0.0009
6	1	6	1	5	1	5	1	4783.1461	0.0000
6	1	6	0	5	1	5	0	4783.5925	0.0002
6	1	5	1	5	1	4	1	5265.2076	0.0023
6	1	5	0	5	1	4	0	5266.5802	0.0018
7	1	6	1	6	1	5	1	6005.3201	0.0009
7	1	6	0	6	1	5	0	6006.6271	0.0001
8	1	7	1	7	1	6	1	6756.3554	0.0040
8	1	7	0	7	1	6	0	6757.6251	0.0028
9	1	8	1	8	1	7	1	7512.9600	0.0005
9	1	8	0	8	1	7	0	7514.2058	-0.0004
3	2	2	0	2	2	1	0	2728.4649	-0.0083
3	2	2	1	2	2	1	1	2730.7769	-0.0040
3	2	1	0	2	2	0	0	2921.4883	0.0027
3	2	1	1	2	2	0	1	2923.4040	0.0011
4	2	3	1	3	2	2	1	3587.2034	-0.0006
4	2	3	0	3	2	2	0	3588.2059	0.0009
4	2	2	1	3	2	1	1	3941.9013	-0.0024
4	2	2	0	3	2	1	0	3943.6055	-0.0008
5	2	4	1	4	2	3	1	4412.4056	-0.0005
5	2	4	0	4	2	3	0	4413.5982	-0.0001
5	2	3	1	4	2	2	1	4900.0172	0.0003
5	2	3	0	4	2	2	0	4902.1644	-0.0006
6	2	5	1	5	2	4	1	5207.8343	0.0013
6	2	5	0	5	2	4	0	5209.0952	0.0008
6	2	4	1	5	2	3	1	5767.9352	0.0012
6	2	4	0	5	2	3	0	5770.2654	0.0000
7	2	6	0	6	2	5	0	5984.8058	0.0010
7	2	5	1	6	2	4	1	6539.4917	-0.0012
7	2	5	0	6	2	4	0	6541.7685	0.0000
8	2	7	1	7	2	6	1	6749.1912	0.0019
8	2	7	0	7	2	6	0	6750.4589	0.0026
8	2	6	1	7	2	5	1	7262.2595	0.0034
8	2	6	0	7	2	5	0	7264.3732	0.0016
9	2	8	1	8	2	7	1	7510.8083	0.0016
9	2	8	0	8	2	7	0	7512.0588	0.0037
9	2	7	1	8	2	6	1	7989.1663	0.0073
9	2	7	0	8	2	6	0	7991.1540	0.0041
4	3	2	1	3	3	1	1	3716.3365	-0.0012
4	3	2	0	3	3	1	0	3725.3551	-0.0003
4	3	1	1	3	3	0	1	3810.3596	-0.0108
5	3	3	1	4	3	2	1	4638.4105	0.0009
5	3	3	0	4	3	2	0	4638.9640	0.0047
5	3	2	1	4	3	1	1	4869.5526	-0.0043
5	3	2	0	4	3	1	0	4870.5516	-0.0020

Continued on next page

J’	Ka’	Kc’	v’	J	Ka	Kc	v	obs/MHz	obs-cal/MHz
6	3	4	1	5	3	3	1	5517.9806	0.0001
6	3	4	0	5	3	3	0	5519.6855	-0.0007
6	3	3	1	5	3	2	1	5921.0224	0.0007
6	3	3	0	5	3	2	0	5923.5050	0.0007
7	3	5	1	6	3	4	1	6360.0042	0.0005
7	3	5	0	6	3	4	0	6361.9095	0.0016
7	3	4	1	6	3	3	1	6903.0102	-0.0013
7	3	4	0	6	3	3	0	6905.9362	-0.0016
8	3	6	1	7	3	5	1	7167.1118	0.0023
8	3	5	1	7	3	4	1	7783.2976	-0.0067
8	3	5	0	7	3	4	0	7786.3612	-0.0036
9	3	7	1	8	3	6	1	7949.3372	0.0044
9	3	7	0	8	3	6	0	7951.2990	0.0034
6	4	3	1	5	4	2	1	5615.0729	-0.0006
6	4	3	0	5	4	2	0	5632.3638	0.0035
6	4	2	1	5	4	1	1	5735.3468	0.0000
6	4	2	0	5	4	1	0	5752.8261	-0.0025
7	4	4	1	6	4	3	1	6561.0146	0.0029
7	4	4	0	6	4	3	0	6562.5239	0.0059
7	4	3	1	6	4	2	1	6820.0733	-0.0020
7	4	3	0	6	4	2	0	6822.0855	0.0002
8	4	5	1	7	4	4	1	7457.0613	0.0037
8	4	5	0	7	4	4	0	7459.3481	-0.0006
8	4	4	1	7	4	3	1	7900.6943	-0.0021
8	4	4	0	7	4	3	0	7903.8380	-0.0052
7	5	3	0	6	5	2	0	6587.2981	0.0011
7	5	3	1	6	5	2	1	6598.7062	-0.0029
7	5	2	0	6	5	1	0	6605.2515	-0.0086
7	5	2	1	6	5	1	1	6616.6190	0.0057
8	5	4	0	7	5	3	0	7550.3159	0.0011
8	5	3	1	7	5	2	1	7666.0514	-0.0101
4	1	4	1	3	0	3	1	3288.8377	-0.0008
4	1	4	0	3	0	3	0	3289.3387	-0.0026
5	1	5	1	4	0	4	1	4031.9205	-0.0008
5	1	5	0	4	0	4	0	4032.3855	0.0009
6	1	6	1	5	0	5	1	4786.4029	0.0005
6	1	6	0	5	0	5	0	4786.8516	0.0000
4	0	4	1	3	1	3	1	3240.5414	-0.0040
4	0	4	0	3	1	3	0	3240.9618	0.0065
5	0	5	1	4	1	4	1	4017.1154	0.0055
5	0	5	0	4	1	4	0	4017.5500	-0.0009
6	0	6	1	5	1	5	1	4782.2895	0.0016
6	0	6	0	5	1	5	0	4782.7336	-0.0004
2	2	1	1	1	1	0	1	2515.5660	0.0020
2	2	1	0	1	1	0	0	2520.8174	-0.0025
3	2	2	1	2	1	1	1	3279.4359	-0.0019
3	2	2	0	2	1	1	0	3281.5570	-0.0007
4	2	3	1	3	1	2	1	3965.9457	0.0058
4	2	3	0	3	1	2	0	3967.8117	0.0091
5	2	4	1	4	1	3	1	4619.9283	-0.0007

Continued on next page

J'	Ka'	Kc'	v'	J	Ka	Kc	v	obs/MHz	obs-cal/MHz
6	2	5	1	5	1	4	1	5297.1441	0.0013
6	2	5	0	5	1	4	0	5298.5511	0.0045
7	2	6	1	6	1	5	1	6015.4663	0.0009
7	2	6	0	6	1	5	0	6016.7733	0.0005
8	2	7	1	7	1	6	1	6759.3394	0.0038
8	2	7	0	7	1	6	0	6760.6046	0.0024
9	2	8	1	8	1	7	1	7513.7822	-0.0085
9	2	8	0	8	1	7	0	7515.0410	0.0062
4	1	3	1	3	2	2	1	3379.6806	-0.0005
4	1	3	0	3	2	2	0	3380.2714	-0.0034
5	1	4	1	4	2	3	1	4323.0957	-0.0005
5	1	4	0	4	2	3	0	4324.1453	-0.0010
6	1	5	1	5	2	4	1	5175.8949	-0.0004
6	1	5	0	5	2	4	0	5177.1259	-0.0004
7	1	6	1	6	2	5	1	5973.3794	-0.0022
7	1	6	0	6	2	5	0	5974.6573	-0.0016
8	1	7	1	7	2	6	1	6746.1912	-0.0139
8	1	7	0	7	2	6	0	6747.4846	0.0081
9	1	8	1	8	2	7	1	7509.9750	-0.0003
9	1	8	0	8	2	7	0	7511.2263	-0.0001
2	2	0	0	1	1	1	0	2735.5639	0.0027
3	2	1	1	2	1	2	1	3983.8884	0.0042
4	2	2	1	3	1	3	1	5454.5034	0.0088
4	3	2	1	3	2	1	1	4788.9862	0.0000
4	3	2	0	3	2	1	0	4793.4266	-0.0046
5	3	3	1	4	2	2	1	5485.4968	0.0047
6	3	4	1	5	2	3	1	6103.4575	0.0018
6	3	4	0	5	2	3	0	6106.3029	-0.0024
7	3	5	0	6	2	4	0	6697.9505	0.0026
8	3	6	1	7	2	5	1	7323.1440	0.0021
8	3	6	0	7	2	5	0	7325.2622	0.0048
4	2	2	0	3	3	1	0	2875.5316	0.0011
5	2	3	0	4	3	2	0	4052.3430	0.0028
5	2	3	1	4	3	2	1	4052.9377	0.0033
6	2	4	1	5	3	3	1	5182.4558	-0.0029
6	2	4	0	5	3	3	0	5183.6420	-0.0042
7	2	5	1	6	3	4	1	6203.9828	0.0115
7	2	5	0	6	3	4	0	6205.7304	0.0018
8	2	6	1	7	3	5	1	7106.2234	-0.0002
8	2	6	0	7	3	5	0	7108.1933	0.0009
9	2	7	1	8	3	6	1	7928.2824	0.0093
3	3	0	0	2	2	1	0	4069.7082	0.0121
3	3	0	1	2	2	1	1	4076.0412	0.0037
4	3	1	1	3	2	2	1	5155.6290	0.0020
4	3	1	0	3	2	2	0	5160.7761	0.0020
5	3	2	1	4	2	3	1	6437.9838	0.0038
5	3	2	0	4	2	3	0	6443.1319	0.0091
5	4	2	0	4	3	1	0	6312.6862	-0.0050
5	4	2	1	4	3	1	1	6323.8469	-0.0086
6	4	3	1	5	3	2	1	7069.3731	0.0010

Continued on next page

J’	Ka’	Kc’	v’	J	Ka	Kc	v	obs/MHz	obs-cal/MHz
6	4	3	0	5	3	2	0	7074.4933	-0.0045
7	4	4	1	6	3	3	1	7709.3581	-0.0039
7	4	4	0	6	3	3	0	7713.5028	-0.0087
5	4	1	0	4	3	2	0	6445.3528	-0.0052
5	4	1	1	4	3	2	1	6456.2727	-0.0061
6	4	2	1	5	3	3	1	7553.2133	-0.0028
6	4	2	0	5	3	3	0	7559.2237	-0.0036
8	3	5	1	7	4	4	1	6976.9541	0.0003
8	3	5	0	7	4	4	0	6978.7875	-0.0035

Table S23: Measured frequencies of the rotational transitions of FACD-PA@PhF species.

J'	Ka'	Kc'	v'	J	Ka	Kc	v	obs/MHz	obs-cal/MHz
7	0	7	1	6	1	6	1	5486.5419	-0.0007
7	1	7	1	6	1	6	1	5486.7501	-0.0030
7	0	7	0	6	1	6	0	5486.9882	0.0001
7	1	7	0	6	1	6	0	5487.1982	-0.0006
7	0	7	1	6	0	6	1	5487.3788	-0.0024
7	1	7	1	6	0	6	1	5487.5960	0.0040
7	0	7	0	6	0	6	0	5487.8268	-0.0012
7	1	7	0	6	0	6	0	5488.0338	-0.0049
8	0	8	1	7	1	7	1	6239.0109	0.0162
8	1	8	1	7	1	7	1	6239.0109	0.0162
8	0	8	1	7	0	7	1	6239.1862	-0.0191
8	1	8	1	7	0	7	1	6239.1862	-0.0191
8	0	8	0	7	1	7	0	6239.4588	0.0221
8	1	8	0	7	1	7	0	6239.4588	0.0221
8	0	8	0	7	0	7	0	6239.6271	-0.0204
8	1	8	0	7	0	7	0	6239.6271	-0.0204
9	0	9	1	8	1	8	1	6991.1580	-0.0047
9	1	9	1	8	1	8	1	6991.1580	-0.0047
9	0	9	1	8	0	8	1	6991.1580	-0.0047
9	1	9	1	8	0	8	1	6991.1580	-0.0047
9	0	9	0	8	1	8	0	6991.5991	-0.0018
9	1	9	0	8	1	8	0	6991.5991	-0.0018
9	0	9	0	8	0	8	0	6991.5991	-0.0018
9	1	9	0	8	0	8	0	6991.5991	-0.0018
10	0	10	1	9	1	9	1	7743.2359	-0.0003
10	1	10	1	9	1	9	1	7743.2359	-0.0003
10	0	10	1	9	0	9	1	7743.2359	-0.0003
10	1	10	1	9	0	9	1	7743.2359	-0.0003
10	0	10	0	9	1	9	0	7743.6726	0.0021
10	1	10	0	9	1	9	0	7743.6726	0.0021
10	0	10	0	9	0	9	0	7743.6726	0.0021
10	1	10	0	9	0	9	0	7743.6726	0.0021
3	0	3	1	2	0	2	1	2505.2368	-0.0011
3	0	3	0	2	0	2	0	2505.7612	-0.0026
4	0	4	1	3	0	3	1	3243.9558	-0.0025
4	0	4	0	3	0	3	0	3244.4552	0.0022
5	0	5	1	4	0	4	1	3987.3895	-0.0030
5	0	5	0	4	0	4	0	3987.8558	-0.0035
6	0	6	1	5	0	5	1	4736.2815	-0.0023
6	0	6	0	5	0	5	0	4736.7364	-0.0010
3	1	3	1	2	1	2	1	2446.4738	0.0067
3	1	3	0	2	1	2	0	2446.8344	-0.0010
3	1	2	1	2	1	1	1	2874.3184	0.0045
3	1	2	0	2	1	1	0	2875.5310	0.0019
4	1	4	1	3	1	3	1	3219.0777	0.0001
4	1	4	0	3	1	3	0	3219.5042	0.0033

Continued on next page

J'	Ka'	Kc'	v'	J	Ka	Kc	v	obs/MHz	obs-cal/MHz
4	1	3	1	3	1	2	1	3723.2037	0.0034
4	1	3	0	3	1	2	0	3724.6460	0.0015
5	1	5	1	4	1	4	1	3979.2195	-0.0015
5	1	5	0	4	1	4	0	3979.6629	-0.0015
5	1	4	1	4	1	3	1	4486.8703	0.0005
5	1	4	0	4	1	3	0	4488.3541	0.0005
6	1	6	1	5	1	5	1	4733.9276	-0.0029
6	1	6	0	5	1	5	0	4734.3764	-0.0015
6	1	5	1	5	1	4	1	5213.4509	-0.0013
6	1	5	0	5	1	4	0	5214.8578	-0.0014
7	1	6	1	6	1	5	1	5945.7397	0.0001
7	1	6	0	6	1	5	0	5947.0731	0.0013
8	1	7	1	7	1	6	1	6688.8754	0.0000
8	1	7	0	7	1	6	0	6690.1633	0.0001
9	1	8	1	8	1	7	1	7437.5193	0.0056
9	1	8	0	8	1	7	0	7438.7779	0.0039
4	2	3	1	3	2	2	1	3553.0781	-0.0125
4	2	3	0	3	2	2	0	3554.0393	-0.0054
4	2	2	1	3	2	1	1	3906.9159	-0.0054
4	2	2	0	3	2	1	0	3908.5110	-0.0047
5	2	4	1	4	2	3	1	4369.8100	0.0039
5	2	4	0	4	2	3	0	4370.9737	0.0016
5	2	3	1	4	2	2	1	4855.8472	0.0076
5	2	3	0	4	2	2	0	4857.9200	0.0063
6	2	5	1	5	2	4	1	5156.8678	0.0019
6	2	5	0	5	2	4	0	5158.1172	0.0003
6	2	4	1	5	2	3	1	5714.5330	0.0033
6	2	4	0	5	2	3	0	5716.8516	0.0022
7	2	6	1	6	2	5	1	5924.3300	0.0018
7	2	6	0	6	2	5	0	5925.6085	0.0024
7	2	5	1	6	2	4	1	6477.2276	-0.0024
7	2	5	0	6	2	4	0	6479.5579	0.0000
8	2	7	1	7	2	6	1	6681.8640	0.0011
8	2	7	0	7	2	6	0	6683.1384	0.0014
8	2	6	1	7	2	5	1	7191.9236	-0.0034
8	2	6	0	7	2	5	0	7194.1091	-0.0021
9	2	8	1	8	2	7	1	7435.4129	0.0000
9	2	8	0	8	2	7	0	7436.6702	-0.0009
9	2	7	1	8	2	6	1	7911.1450	0.0011
9	2	7	0	8	2	6	0	7913.1795	-0.0085
5	3	3	1	4	3	2	1	4595.6155	-0.0092
5	3	3	0	4	3	2	0	4595.9661	-0.0097
5	3	2	1	4	3	1	1	4826.6436	-0.0040
5	3	2	0	4	3	1	0	4827.3510	-0.0032
6	3	4	1	5	3	3	1	5466.3331	0.0049
6	3	4	0	5	3	3	0	5467.9655	0.0030
6	3	3	1	5	3	2	1	5868.7963	0.0038
6	3	3	0	5	3	2	0	5871.1128	0.0070
7	3	5	1	6	3	4	1	6299.6641	0.0039
7	3	5	0	6	3	4	0	6301.5257	0.0020

Continued on next page

J'	Ka'	Kc'	v'	J	Ka	Kc	v	obs/MHz	obs-cal/MHz
7	3	4	1	6	3	3	1	6841.1516	0.0014
7	3	4	0	6	3	3	0	6843.9734	0.0027
8	3	6	1	7	3	5	1	7098.2229	0.0007
8	3	6	0	7	3	5	0	7100.1785	0.0016
8	3	5	1	7	3	4	1	7711.7384	-0.0041
8	3	5	0	7	3	4	0	7714.7874	-0.0058
9	3	7	1	8	3	6	1	7872.0882	0.0012
7	4	4	1	6	4	3	1	6500.8590	0.0007
7	4	4	0	6	4	3	0	6502.1830	0.0028
7	4	3	1	6	4	2	1	6760.3189	0.0034
7	4	3	0	6	4	2	0	6761.9966	-0.0107
8	4	5	1	7	4	4	1	7387.8967	0.0016
8	4	5	0	7	4	4	0	7390.0962	0.0042
8	4	4	1	7	4	3	1	7831.3921	-0.0098
8	4	4	0	7	4	3	0	7834.3180	0.0004
4	1	4	1	3	0	3	1	3255.3377	0.0158
4	1	4	0	3	0	3	0	3255.8453	-0.0019
5	1	5	1	4	0	4	1	3990.5802	-0.0044
5	1	5	0	4	0	4	0	3991.0534	-0.0054
6	1	6	1	5	0	5	1	4737.1222	-0.0004
6	1	6	0	5	0	5	0	4737.5758	-0.0015
4	0	4	1	3	1	3	1	3207.7200	0.0060
4	0	4	0	3	1	3	0	3208.1011	-0.0053
5	0	5	1	4	1	4	1	3976.0269	-0.0021
5	0	5	0	4	1	4	0	3976.4628	-0.0021
6	0	6	1	5	1	5	1	4733.0882	-0.0036
6	0	6	0	5	1	5	0	4733.5345	-0.0034
5	2	4	1	4	1	3	1	4574.7843	-0.0040
6	2	5	1	5	1	4	1	5244.7916	0.0070
6	2	5	0	5	1	4	0	5246.2608	0.0002
7	2	6	1	6	1	5	1	5955.6629	0.0024
7	2	6	0	6	1	5	0	5957.0110	0.0037
8	2	7	1	7	1	6	1	6691.7832	-0.0004
8	2	7	0	7	1	6	0	6693.0727	0.0002
9	2	8	1	8	1	7	1	7438.3186	-0.0026
9	2	8	0	8	1	7	0	7439.5830	0.0026
5	1	4	1	4	2	3	1	4281.8852	-0.0022
5	1	4	0	4	2	3	0	4282.8278	-0.0005
6	1	5	1	5	2	4	1	5125.5427	0.0090
6	1	5	0	5	2	4	0	5126.7253	0.0096
7	1	6	1	6	2	5	1	5914.4121	0.0047
7	1	6	0	6	2	5	0	5915.6732	0.0026
8	1	7	1	7	2	6	1	6678.9551	0.0006
8	1	7	0	7	2	6	0	6680.2295	0.0018
9	1	8	1	8	2	7	1	7434.6001	-0.0052
9	1	8	0	8	2	7	0	7435.8675	0.0028
4	3	2	1	3	2	1	1	4746.2159	0.0042
4	3	2	0	3	2	1	0	4750.9846	-0.0052
5	3	3	0	4	2	2	0	5438.4572	0.0073
6	3	4	0	5	2	3	0	6048.4992	0.0005

Continued on next page

J'	Ka'	Kc'	v'	J	Ka	Kc	v	obs/MHz	obs-cal/MHz
7	3	5	1	6	2	4	1	6630.5264	-0.0075
7	3	5	0	6	2	4	0	6633.1687	-0.0043
8	3	6	1	7	2	5	1	7251.5250	-0.0011
8	3	6	0	7	2	5	0	7253.7970	0.0050
9	3	7	1	8	2	6	1	7931.6853	-0.0007
7	2	5	1	6	3	4	1	6146.3582	0.0019
8	2	6	1	7	3	5	1	7038.6256	0.0024
8	2	6	0	7	3	5	0	7040.4993	0.0032
6	4	3	1	5	3	2	1	7005.6966	-0.0064
7	4	4	1	6	3	3	1	7637.7771	0.0083
7	4	4	0	6	3	3	0	7642.3014	-0.0028
5	4	1	1	4	3	2	1	6402.2558	-0.0028
6	4	2	1	5	3	3	1	7490.8754	-0.0022
6	4	2	0	5	3	3	0	7497.0370	0.0023

Table S25: Measured frequencies of the rotational transitions of FAOD-PA@PhF species.

J'	Ka'	Kc'	J	Ka	Kc	obs/MHz	obs-cal/MHz
9	0	9	8	1	8	7042.1690	0.0004
9	1	9	8	1	8	7042.1690	0.0004
9	0	9	8	0	8	7042.1690	0.0004
9	1	9	8	0	8	7042.1690	0.0004
10	0	10	9	1	9	7799.8745	0.0004
10	1	10	9	1	9	7799.8745	0.0004
10	0	10	9	0	9	7799.8745	0.0004
10	1	10	9	0	9	7799.8745	0.0004
3	0	3	2	0	2	2522.3262	-0.0042
4	0	4	3	0	3	3266.5951	-0.0020
5	0	5	4	0	4	4015.7587	-0.0006
6	0	6	5	0	5	4770.3478	-0.0026
7	0	7	6	0	6	5527.1084	-0.0001
3	1	2	2	1	1	2893.6148	-0.0004
4	1	4	3	1	3	3242.1292	0.0019
4	1	3	3	1	2	3747.3799	0.0033
5	1	5	4	1	4	4007.7976	0.0054
5	1	4	4	1	3	4515.5663	0.0002
6	1	6	5	1	5	4768.0730	-0.0020
6	1	5	5	1	4	5247.5160	-0.0012
7	1	7	6	1	6	5526.5009	-0.0054
7	1	6	6	1	5	5985.6776	0.0001
8	1	7	7	1	6	6734.6513	-0.0002
9	1	8	8	1	7	7489.0112	-0.0080
3	2	2	2	2	1	2720.5056	0.0067
3	2	1	2	2	0	2918.6817	0.0082
4	2	3	3	2	2	3577.5075	-0.0083
5	2	4	4	2	3	4399.7619	-0.0002
5	2	3	4	2	2	4888.2960	0.0023
6	2	5	5	2	4	5192.1152	0.0039
6	2	4	5	2	3	5750.9634	0.0031
7	2	6	6	2	5	5964.9209	0.0073
7	2	5	6	2	4	6517.3326	-0.0018
8	2	7	7	2	6	6727.9203	0.0082
8	2	6	7	2	5	7237.0826	-0.0004
9	2	8	8	2	7	7487.0291	0.0111
9	2	7	8	2	6	7962.3364	-0.0009
5	3	3	4	3	2	4625.8921	-0.0031
5	3	2	4	3	1	4862.4066	-0.0077
6	3	4	5	3	3	5503.5399	0.0045
6	3	3	5	3	2	5910.2382	0.0054
7	3	5	6	3	4	6342.1270	0.0031
7	3	4	6	3	3	6886.9524	-0.0027
8	3	6	7	3	5	7145.6881	0.0022
8	3	5	7	3	4	7760.4474	-0.0107
9	3	7	8	3	6	7924.6478	-0.0025

Continued on next page

J'	Ka'	Kc'	J	Ka	Kc	obs/MHz	obs-cal/MHz
6	4	3	5	4	2	5616.5717	-0.0153
6	4	2	5	4	1	5728.1540	-0.0075
7	4	4	6	4	3	6544.6476	0.0077
7	4	3	6	4	2	6810.9206	-0.0005
8	4	4	7	4	3	7887.3599	-0.0024
8	5	4	7	5	3	7530.0830	0.0045
8	5	3	7	5	2	7656.8002	0.0056
4	1	4	3	0	3	3277.6420	0.0001
5	1	5	4	0	4	4018.8401	0.0032
6	1	6	5	0	5	4771.1657	0.0133
7	1	7	6	0	6	5527.3116	0.0032
4	0	4	3	1	3	3231.0802	-0.0024
5	0	5	4	1	4	4004.7137	-0.0010
6	0	6	5	1	5	4767.2601	-0.0129
7	0	7	6	1	6	5526.2896	-0.0170
8	0	8	7	1	7	6284.3534	0.0038
4	2	3	3	1	2	3948.8544	0.0033
5	2	4	4	1	3	4601.2301	-0.0064
7	2	6	6	1	5	5995.1809	0.0026
8	2	7	7	1	6	6737.4197	0.0068
7	1	6	6	2	5	5955.4207	0.0079
8	1	7	7	2	6	6725.1505	-0.0001
9	1	8	8	2	7	7486.2468	-0.0110
3	3	0	2	2	1	4050.9343	-0.0048
4	3	2	3	2	1	4767.8304	0.0054
6	3	4	5	2	3	6074.7939	-0.0080
7	3	5	6	2	4	6665.9629	-0.0027
8	3	6	7	2	5	7294.3174	0.0002
4	3	1	3	2	2	5135.7679	0.0029
5	3	2	4	2	3	6420.6661	0.0026
7	2	5	6	3	4	6193.4983	0.0055
6	4	3	5	3	2	7035.9874	0.0092
7	4	4	6	3	3	7670.3866	0.0013
5	4	1	4	3	2	6424.1162	-0.0006
6	4	2	5	3	3	7526.3751	-0.0079

Table S27: Measured frequencies of the rotational transitions of FA-PAOD@PhF species.

J'	Ka'	Kc'	J	Ka	Kc	obs/MHz	obs-cal/MHz
8	1	8	7	1	7	6288.3445	-0.0035
8	0	8	7	0	7	6288.3445	-0.0035
9	0	9	8	1	8	7046.5766	-0.0023
9	1	9	8	1	8	7046.5766	-0.0023
9	0	9	8	0	8	7046.5766	-0.0023
9	1	9	8	0	8	7046.5766	-0.0023
10	0	10	9	1	9	7804.8167	-0.0040
10	1	10	9	1	9	7804.8167	-0.0040
10	0	10	9	0	9	7804.8167	-0.0040
10	1	10	9	0	9	7804.8167	-0.0040
3	0	3	2	0	2	2523.5584	-0.0014
4	0	4	3	0	3	3268.4184	0.0000
5	0	5	4	0	4	4018.0768	-0.0001
6	0	6	5	0	5	4773.1700	-0.0031
7	0	7	6	0	6	5530.4538	0.0001
3	1	2	2	1	1	2893.3370	-0.0043
4	1	4	3	1	3	3243.7687	0.0008
4	1	3	3	1	2	3747.8940	0.0001
5	1	5	4	1	4	4010.0209	0.0042
5	1	4	4	1	3	4517.1437	-0.0027
6	1	6	5	1	5	4770.8625	0.0007
6	1	5	5	1	4	5249.7810	-0.0016
7	1	7	6	1	6	5529.8389	-0.0006
7	1	6	6	1	5	5988.3720	-0.0013
9	1	8	8	1	7	7492.6382	-0.0018
5	2	4	4	2	3	4400.8168	0.0010
5	2	3	4	2	2	4887.5966	0.0019
6	2	5	5	2	4	5193.8457	0.0002
6	2	4	5	2	3	5751.4749	-0.0032
7	2	6	6	2	5	5967.3118	-0.0001
7	2	5	6	2	4	6519.3136	-0.0064
8	2	7	7	2	6	6730.9257	0.0058
8	2	6	7	2	5	7239.9068	-0.0007
9	2	8	8	2	7	7490.5961	0.0031
9	2	7	8	2	6	7965.5107	-0.0034
5	3	3	4	3	2	4625.6529	-0.0095
5	3	2	4	3	1	4860.2814	-0.0064
6	3	4	5	3	3	5503.7878	0.0028
6	3	3	5	3	2	5908.0924	0.0006
7	3	5	6	3	4	6343.0911	0.0009
7	3	4	6	3	3	6885.6877	-0.0017
8	3	6	7	3	5	7147.4660	0.0029
8	3	5	7	3	4	7760.9164	0.0049
9	3	7	8	3	6	7927.1966	-0.0005
6	4	3	5	4	2	5615.7052	0.0029
6	4	2	5	4	1	5725.8134	-0.0080

Continued on next page

J'	Ka'	Kc'	J	Ka	Kc	obs/MHz	obs-cal/MHz
7	4	3	6	4	2	6807.5543	-0.0027
8	4	4	7	4	3	7884.0616	-0.0061
8	5	4	7	5	3	7528.6422	0.0073
8	5	3	7	5	2	7653.3326	-0.0012
4	1	4	3	0	3	3279.6159	0.0066
5	1	5	4	0	4	4021.2121	0.0047
6	1	6	5	0	5	4773.9978	0.0057
7	1	7	6	0	6	5530.6564	-0.0021
5	0	5	4	1	4	4006.8838	-0.0024
6	0	6	5	1	5	4770.0378	-0.0050
7	0	7	6	1	6	5529.6409	0.0060
5	2	4	4	1	3	4603.8377	-0.0018
6	2	5	5	1	4	5280.5356	-0.0028
7	2	6	6	1	5	5998.0684	0.0006
8	2	7	7	1	6	6740.6212	0.0068
6	1	5	5	2	4	5163.0908	0.0011
7	1	6	6	2	5	5957.6272	0.0096
8	1	7	7	2	6	6728.0869	-0.0041
9	1	8	8	2	7	7489.8149	0.0036
4	3	2	3	2	1	4769.5894	0.0043
5	3	3	4	2	2	5462.2893	0.0068
6	3	4	5	2	3	6078.4620	-0.0106
7	3	5	6	2	4	6670.0788	-0.0059
8	3	6	7	2	5	7298.2278	0.0000
9	3	7	8	2	6	7985.5194	0.0019
4	3	1	3	2	2	5134.8133	-0.0052
5	3	2	4	2	3	6417.1381	0.0115
7	2	5	6	3	4	6192.3315	0.0061
8	2	6	7	3	5	7089.1422	-0.0004

S12 Rotational transitions of the FA-PA@PhF···He complex

Table S29: Measured frequencies of the rotational transitions of FA-PA@PhF···He species.

J'	Ka'	Kc'	v'	J	Ka	Kc	v	obs/MHz	obs-cal/MHz
3	0	3	1	2	0	2	1	2379.8027	-0.0079
3	0	3	0	2	0	2	0	2380.1225	0.0072
4	0	4	1	3	0	3	1	3082.6853	-0.0019
4	0	4	0	3	0	3	0	3082.9889	-0.0015
5	0	5	1	4	0	4	1	3793.5160	0.0000
5	0	5	0	4	0	4	0	3793.8275	-0.0003
6	0	6	1	5	0	5	1	4507.3857	-0.0038
6	0	6	0	5	0	5	0	4507.7008	-0.0028
3	1	3	1	2	1	2	1	2345.5108	-0.0075
3	1	3	0	2	1	2	0	2345.8685	0.0017
3	1	2	1	2	1	1	1	2804.5103	0.0016
3	1	2	0	2	1	1	0	2805.6710	0.0012
4	1	4	1	3	1	3	1	3073.1605	0.0002
4	1	4	0	3	1	3	0	3073.4970	0.0009
4	1	3	1	3	1	2	1	3568.2210	0.0006
4	1	3	0	3	1	2	0	3569.2125	0.0000
5	1	5	1	4	1	4	1	3791.4171	-0.0008
5	1	5	0	4	1	4	0	3791.7434	0.0017
5	1	4	1	4	1	3	1	4260.1188	0.0009
5	1	4	0	4	1	3	0	4260.9778	-0.0084
6	1	6	1	5	1	5	1	4506.9746	-0.0038
6	1	6	0	5	1	5	0	4507.2931	-0.0029
6	1	5	1	5	1	4	1	4954.1324	0.0006
6	1	5	0	5	1	4	0	4955.0143	0.0002
7	1	6	1	6	1	5	1	5661.2563	0.0006
7	1	6	0	6	1	5	0	5662.1585	0.0012
8	1	7	1	7	1	6	1	6373.7683	0.0035
8	2	7	0	7	2	6	0	6373.7683	0.0035
8	1	7	0	7	1	6	0	6374.6660	0.0022
9	1	8	1	8	1	7	1	7087.8270	-0.0020
9	1	8	0	8	1	7	0	7088.7197	-0.0074
7	0	7	1	6	1	6	1	5221.9247	-0.0030
7	1	7	1	6	1	6	1	5221.9247	-0.0030
7	0	7	1	6	0	6	1	5222.0127	-0.0057
7	1	7	1	6	0	6	1	5222.0127	-0.0057
7	0	7	0	6	1	6	0	5222.2407	-0.0012
7	1	7	0	6	1	6	0	5222.2407	-0.0012
7	0	7	0	6	0	6	0	5222.3286	-0.0031
7	1	7	0	6	0	6	0	5222.3286	-0.0031
8	0	8	1	7	1	7	1	5936.7848	-0.0023
8	1	8	1	7	1	7	1	5936.7848	-0.0023
8	0	8	1	7	0	7	1	5936.7848	-0.0023
8	1	8	1	7	0	7	1	5936.7848	-0.0023
8	0	8	0	7	1	7	0	5937.0980	-0.0006

Continued on next page

J'	Ka'	Kc'	v'	J	Ka	Kc	v	obs/MHz	obs-cal/MHz
8	1	8	0	7	1	7	0	5937.0980	-0.0006
8	0	8	0	7	0	7	0	5937.0980	-0.0006
8	1	8	0	7	0	7	0	5937.0980	-0.0006
9	0	9	1	8	1	8	1	6651.6005	-0.0075
9	1	9	1	8	1	8	1	6651.6005	-0.0075
9	0	9	1	8	0	8	1	6651.6005	-0.0075
9	1	9	1	8	0	8	1	6651.6005	-0.0075
9	0	9	0	8	1	8	0	6651.9114	-0.0057
9	1	9	0	8	1	8	0	6651.9114	-0.0057
9	0	9	0	8	0	8	0	6651.9114	-0.0057
9	1	9	0	8	0	8	0	6651.9114	-0.0057
10	0	10	1	9	1	9	1	7366.4142	-0.0131
10	1	10	1	9	1	9	1	7366.4142	-0.0131
10	0	10	1	9	0	9	1	7366.4142	-0.0131
10	1	10	1	9	0	9	1	7366.4142	-0.0131
10	0	10	0	9	1	9	0	7366.7245	-0.0097
10	1	10	0	9	1	9	0	7366.7245	-0.0097
10	0	10	0	9	0	9	0	7366.7245	-0.0097
10	1	10	0	9	0	9	0	7366.7245	-0.0097
3	2	2	1	2	2	1	1	2641.1214	0.0057
3	2	2	0	2	2	1	0	2641.7367	0.0036
3	2	1	1	2	2	0	1	2901.3476	0.0047
3	2	1	0	2	2	0	0	2902.6376	0.0012
4	2	3	1	3	2	2	1	3445.0283	0.0003
4	2	3	0	3	2	2	0	3446.0608	0.0014
4	2	2	1	3	2	1	1	3870.6604	0.0115
4	2	2	0	3	2	1	0	3872.5916	0.0035
5	2	4	1	4	2	3	1	4205.3977	-0.0003
5	2	4	0	4	2	3	0	4206.4128	-0.0002
5	2	3	1	4	2	2	1	4731.5846	0.0016
6	2	5	1	5	2	4	1	4937.4966	0.0000
6	2	5	0	5	2	4	0	4938.4629	-0.0002
6	2	4	1	5	2	3	1	5465.1449	0.0055
6	2	4	0	5	2	3	0	5466.6106	0.0003
7	2	6	1	6	2	5	1	5657.1445	0.0012
7	2	6	0	6	2	5	0	5658.0777	0.0019
7	2	5	1	6	2	4	1	6140.0197	0.0108
7	2	5	0	6	2	4	0	6141.3411	0.0036
8	2	7	1	7	2	6	1	6372.8585	0.0017
8	2	6	1	7	2	5	1	6826.7635	0.0058
8	2	6	0	7	2	5	0	6828.1247	0.0001
9	2	8	1	8	2	7	1	7087.6546	0.0090
9	2	8	0	8	2	7	0	7088.5556	0.0096
9	2	7	1	8	2	6	1	7530.5126	0.0019
4	3	2	0	3	3	1	0	3639.9549	-0.0063
4	3	1	1	3	3	0	1	3803.6611	0.0094
5	3	3	1	4	3	2	1	4500.7237	0.0113
5	3	3	0	4	3	2	0	4502.3392	-0.0028
5	3	2	1	4	3	1	1	4854.5490	-0.0034
5	3	2	0	4	3	1	0	4857.1843	-0.0002

Continued on next page

J’	Ka’	Kc’	v’	J	Ka	Kc	v	obs/MHz	obs-cal/MHz
6	3	4	1	5	3	3	1	5310.1179	0.0018
6	3	4	0	5	3	3	0	5311.8168	0.0027
6	3	3	1	5	3	2	1	5822.5073	-0.0039
6	3	3	0	5	3	2	0	5825.2649	-0.0043
7	3	5	1	6	3	4	1	6072.5409	0.0026
7	3	5	0	6	3	4	0	6074.1485	0.0029
7	3	4	1	6	3	3	1	6656.2367	-0.0020
7	3	4	0	6	3	3	0	6658.5545	-0.0099
8	3	6	1	7	3	5	1	6805.3100	0.0021
8	3	6	0	7	3	5	0	6806.8271	0.0073
9	3	7	1	8	3	6	1	7524.8312	0.0019
9	3	7	0	8	3	6	0	7526.2693	-0.0083
4	3	1	1	3	1	2	1	5107.7509	-0.0012
4	3	1	0	3	1	2	0	5110.6263	-0.0018
4	3	2	0	3	1	3	0	5862.9910	0.0027
6	4	3	1	5	4	2	1	5511.0087	0.0040
6	4	3	0	5	4	2	0	5512.5273	-0.0041
6	4	2	1	5	4	1	1	5754.5535	0.0000
7	4	4	1	6	4	3	1	6375.2813	0.0044
7	4	4	0	6	4	3	0	6377.6227	0.0065
7	4	3	1	6	4	2	1	6820.3787	-0.0077
7	4	3	0	6	4	2	0	6824.0040	-0.0048
8	4	5	1	7	4	4	1	7183.8870	-0.0041
8	4	5	0	7	4	4	0	7186.1398	-0.0082
3	1	3	1	2	0	2	1	2391.9389	0.0014
3	1	3	0	2	0	2	0	2392.1931	-0.0001
4	1	4	1	3	0	3	1	3085.2863	-0.0007
4	1	4	0	3	0	3	0	3085.5756	0.0015
5	1	5	1	4	0	4	1	3794.0175	-0.0001
5	1	5	0	4	0	4	0	3794.3264	0.0011
6	1	6	1	5	0	5	1	4507.4760	-0.0042
6	1	6	0	5	0	5	0	4507.7926	-0.0008
3	0	3	1	2	1	2	1	2333.3945	0.0029
3	0	3	0	2	1	2	0	2333.7868	-0.0018
4	0	4	1	3	1	3	1	3070.5606	0.0001
4	0	4	0	3	1	3	0	3070.9142	0.0017
5	0	5	1	4	1	4	1	3790.9197	0.0035
5	0	5	0	4	1	4	0	3791.2457	0.0014
6	0	6	1	5	1	5	1	4506.8836	-0.0041
6	0	6	0	5	1	5	0	4507.2026	-0.0036
2	2	1	0	1	1	0	0	2290.0674	-0.0008
3	2	2	1	2	1	1	1	3004.2926	-0.0055
3	2	2	0	2	1	1	0	3005.1407	-0.0054
4	2	3	1	3	1	2	1	3644.8163	-0.0011
4	2	3	0	3	1	2	0	3645.5332	-0.0023
5	2	4	1	4	1	3	1	4281.9932	-0.0020
5	2	4	0	4	1	3	0	4282.7363	0.0001
6	2	5	1	5	1	4	1	4959.3722	-0.0017
6	2	5	0	5	1	4	0	4960.2117	-0.0012
7	2	6	1	6	1	5	1	5662.3844	-0.0010

Continued on next page

J'	Ka'	Kc'	v'	J	Ka	Kc	v	obs/MHz	obs-cal/MHz
7	2	6	0	6	1	5	0	5663.2707	-0.0041
8	2	7	1	7	1	6	1	6373.9852	-0.0013
8	2	7	0	7	1	6	0	6374.8917	0.0043
4	1	3	1	3	2	2	1	3368.4319	0.0011
5	1	4	1	4	2	3	1	4183.5211	0.0005
5	1	4	0	4	2	3	0	4184.6601	-0.0030
6	1	5	1	5	2	4	1	4932.2542	-0.0001
6	1	5	0	5	2	4	0	4933.2674	0.0032
7	1	6	1	6	2	5	1	5656.0163	0.0028
7	1	6	0	6	2	5	0	5656.9597	0.0014
8	1	7	1	7	2	6	1	6372.6327	0.0028
8	1	7	0	7	2	6	0	6373.5498	0.0034
2	2	1	1	1	1	0	1	2288.8672	-0.0031
2	2	1	0	1	1	0	0	2290.0674	-0.0008
2	2	0	1	1	1	1	1	2545.5478	-0.0022
2	2	0	0	1	1	1	0	2547.3833	0.0058
3	2	1	1	2	1	2	1	3851.7562	0.0038
3	2	1	0	2	1	2	0	3854.5674	-0.0007
4	2	2	1	3	1	3	1	5376.8979	0.0149
4	2	2	0	3	1	3	0	5381.2947	0.0052
9	2	8	1	8	1	7	1	7087.8697	-0.0026
9	2	8	0	8	1	7	0	7088.7679	-0.0017
10	1	9	1	9	2	8	1	7802.2793	-0.0103
10	2	9	1	9	2	8	1	7802.2793	-0.0103
10	1	9	1	9	1	8	1	7802.3476	0.0147
10	2	9	1	9	1	8	1	7802.3476	0.0147
10	1	9	0	9	2	8	0	7803.1694	-0.0101
10	2	9	0	9	2	8	0	7803.1694	-0.0101
10	1	9	0	9	1	8	0	7803.2356	0.0174
10	2	9	0	9	1	8	0	7803.2353	0.0094
3	3	1	0	2	2	0	0	3616.9609	-0.0010
4	3	2	1	3	2	1	1	4352.8601	0.0001
4	3	2	0	3	2	1	0	4354.2732	-0.0137
5	3	3	1	4	2	2	1	4982.9294	0.0058
5	3	3	0	4	2	2	0	4984.0430	0.0020
6	3	4	1	5	2	3	1	5561.4629	0.0063
6	3	4	0	5	2	3	0	5562.4246	-0.0030
7	3	5	1	6	2	4	1	6168.8545	-0.0009
8	3	6	1	7	2	5	1	6834.1565	0.0021
8	3	6	0	7	2	5	0	6835.4530	0.0077
9	3	7	1	8	2	6	1	7532.2234	-0.0025
9	3	7	0	8	2	6	0	7533.5972	-0.0013
3	3	0	0	2	2	1	0	3741.5981	-0.0031
3	3	0	1	2	2	1	1	3745.4208	-0.0058
4	3	1	1	3	2	2	1	4907.9634	0.0008
4	3	1	0	3	2	2	0	4911.1539	0.0021
5	3	2	1	4	2	3	1	6317.4852	-0.0019
5	3	2	0	4	2	3	0	6322.2753	-0.0016
4	2	2	0	3	3	1	0	3158.2697	0.0071
5	2	3	1	4	3	2	1	4249.3639	-0.0079

Continued on next page

J'	Ka'	Kc'	v'	J	Ka	Kc	v	obs/MHz	obs-cal/MHz
6	2	4	1	5	3	3	1	5213.8067	0.0078
6	2	4	0	5	3	3	0	5216.0004	0.0038
7	2	5	1	6	3	4	1	6043.6939	0.0021
7	2	5	0	6	3	4	0	6045.5201	0.0001
8	2	6	1	7	3	5	1	6797.9135	0.0023
8	2	6	0	7	3	5	0	6799.5032	0.0041
9	2	7	1	8	3	6	1	7523.1198	0.0058
9	2	7	0	8	3	6	0	7524.5803	-0.0044
5	4	2	1	4	3	1	1	5730.8727	-0.0046
5	4	2	0	4	3	1	0	5733.5165	0.0010
6	4	3	1	5	3	2	1	6387.3311	0.0014
6	4	3	0	5	3	2	0	6388.8576	-0.0046
7	4	4	1	6	3	3	1	6940.0924	-0.0027
7	4	4	0	6	3	3	0	6941.2112	0.0020
8	4	5	1	7	3	4	1	7467.7503	0.0027
8	4	5	0	7	3	4	0	7468.8108	0.0180
5	4	1	1	4	3	2	1	6030.2067	-0.0025
5	4	1	0	4	3	2	0	6034.1552	0.0059
6	4	2	1	5	3	3	1	7284.0474	-0.0028
6	4	2	0	5	3	3	0	7288.8980	-0.0007
7	3	4	1	6	4	3	1	6091.4096	-0.0108
7	3	4	0	6	4	3	0	6094.9615	-0.0098
5	5	1	0	4	4	0	0	6247.0592	-0.0117
6	5	2	0	5	4	1	0	7096.9121	0.0079
7	5	3	1	6	4	2	1	7820.0466	0.0064
7	5	3	0	6	4	2	0	7822.2072	-0.0089
2	2	0	1	1	0	1	1	2666.7609	-0.0026
2	2	0	0	1	0	1	0	2668.5764	0.0096
3	2	1	1	2	0	2	1	3898.1712	-0.0002
3	2	1	0	2	0	2	0	3900.8967	0.0020
4	2	2	1	3	0	3	1	5389.0067	-0.0030
5	3	2	1	4	1	3	1	6394.0884	0.0042
5	3	2	0	4	1	3	0	6398.6110	0.0108
5	4	1	1	4	2	2	1	6512.4138	-0.0065
5	4	1	0	4	2	2	0	6515.8474	-0.0007
5	4	2	0	4	2	3	0	7198.6115	0.0035
6	4	2	1	5	2	3	1	7535.3731	-0.0177
6	4	2	0	5	2	3	0	7539.5013	-0.0110

S13 Metadynamics simulations

$$\mathrm{CV} = \frac{r_1(\mathrm{OH}) + r_2(\mathrm{OH}) - r_1(\mathrm{O}\cdots\mathrm{H}) - r_2(\mathrm{O}\cdots\mathrm{H})}{2}\ , \tag{12}$$

The metadynamics[38,39,40] simulations of the proton transfer in conformer #1 of FA-PA@PhF were performed using BBBMTD (bond breaking Bohmian metadynamics) script[41] from PyRAMD software (`https://gitlab.desy.de/denis.tikhonov/pyramd`)[42] using ORCA 6 as a source of the potential energy surface at the PBEh-3c level of theory[43]. The time step in the simulations was 1 fs. The equilibrium geometry of FA-FA, FA-PA, FA-FA@PhF, and conformer #1 of FA-PA@PhF at the PBEh-3c level of theory were taken as the initial coordinates for the trajectories, with the initial velocities sampled from the Maxwell-Boltzmann distribution at a temperature of 100 K. To prevent overheating of the system by the time-dependent bias potential, the Berendsen thermostat[44] was used, set to the desired temperature of 100 K and with the relaxation time of 150 fs. The collective variable (CV) was given by Eq. 12, which led to the effective mass of 0.95 Da. The width of the bias potential was 0.5 Å, the update time of the bias potential was 20 fs, and the bias potential components' energies were 36 cm^{-1} (0.4 kJ/mol). Ten trajectories, each 5 ps long, were obtained, but some of them resulted in the dissociation of the clusters into molecular fragments or conformational rearrangement (for FA-PA@PhF) and were therefore disregarded. The number of undissociated trajectories was eight for FA-FA, seven for FA-PA, five for FA-FA@PhF, and four for FA-PA@PhF. The mean potential was calculated and symmetrized by taking the bias point trajectory twice: the normal one and with the CV values multiplied by -1.

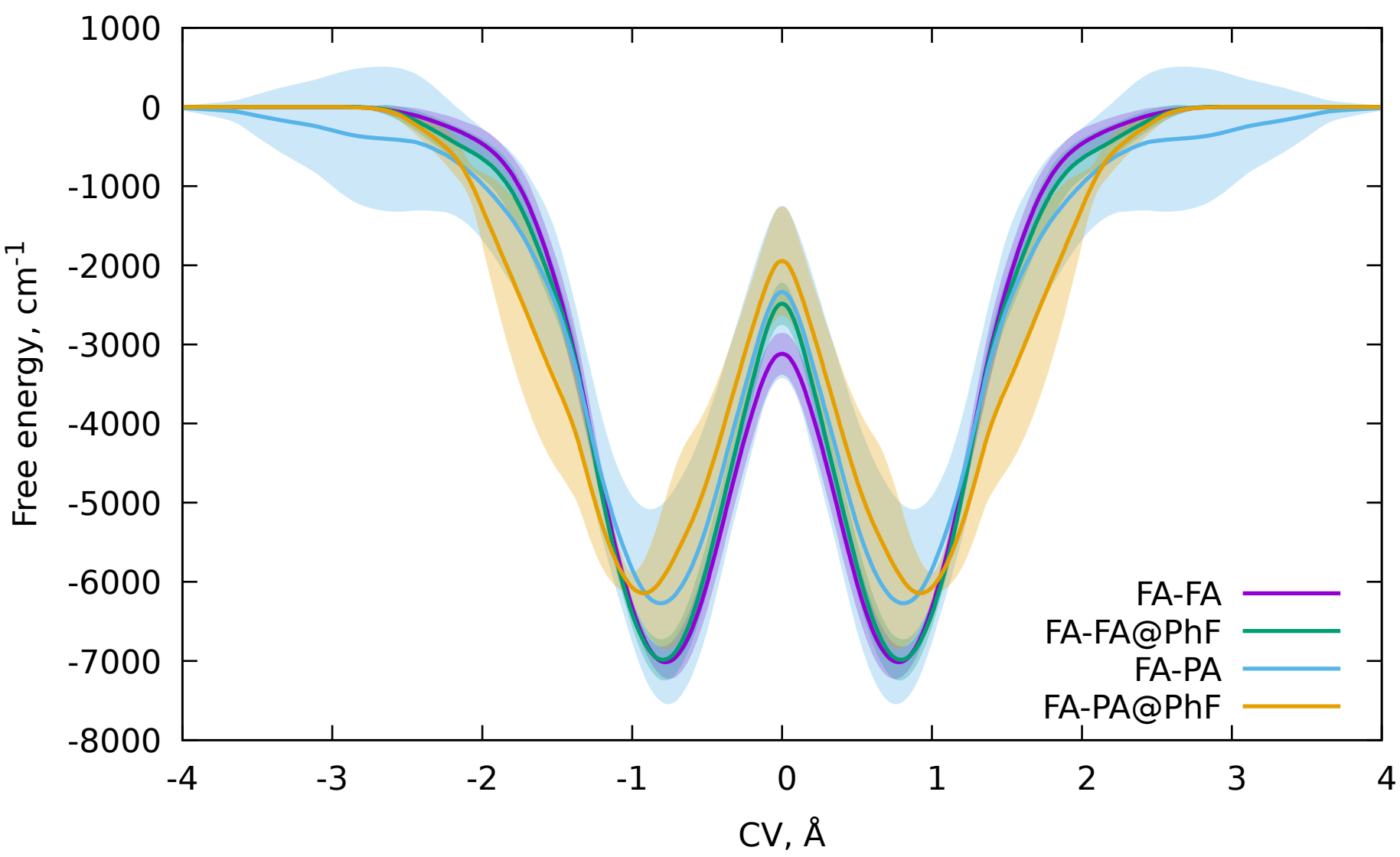

Figure S17: Mean bias potential of the double proton transfer FA-FA, FA-PA, FA-FA@PhF, and conformer #1 of FA-PA@PhF at the PBEh-3c level of theory.

References

[1] Brown, G. G. *et al.* A broadband Fourier transform microwave spectrometer based on chirped pulse excitation. *Rev. Sci. Instrum.* **79** (2008).

[2] Li, W. *et al.* Evolution of solute-water interactions in the benzaldehyde-$(H_2O)_{1-6}$ clusters by rotational spectroscopy. *J. Am. Chem. Soc.* **145**, 4119–4128 (2023).

[3] Kisiel, Z. *et al.* Rotational spectrum of trans-trans diethyl ether in the ground and three excited vibrational states. *J. Mol. Spectrosc.* **233**, 231–243 (2005).

[4] Pickett, H. M. The fitting and prediction of vibration-rotation spectra with spin interactions. *J. Mol. Spectrosc.* **148**, 371–377 (1991).

[5] Pracht, P., Bohle, F. & Grimme, S. Automated exploration of the low-energy chemical space with fast quantum chemical methods. *Phys. Chem. Chem. Phys.* **22**, 7169–7192 (2020).

[6] Becke, A. D. Density-functional thermochemistry. III. The role of exact exchange. *J. Chem. Phys.* **98**, 5648–5652 (1993).

[7] Lee, C., Yang, W. & Parr, R. G. Development of the Colle-Salvetti correlation-energy formula into a functional of the electron density. *Phys. Rev. B* **37**, 785–789 (1988).

[8] Vosko, S. H., Wilk, L. & Nusair, M. Accurate spin-dependent electron liquid correlation energies for local spin density calculations: a critical analysis. *Can. J. Phys.* **58**, 1200–1211 (1980).

[9] Grimme, S., Ehrlich, S. & Goerigk, L. Effect of the damping function in dispersion corrected density functional theory. *J. Comput. Chem.* **32**, 1456–1465 (2011).

[10] Weigend, F. & Ahlrichs, R. Balanced basis sets of split valence, triple zeta valence and quadruple zeta valence quality for H to R_n: design and assessment of accuracy. *Phys. Chem. Chem. Phys.* **7**, 3297–3305 (2005).

[11] Neese, F., Wennmohs, F., Becker, U. & Riplinger, C. The ORCA quantum chemistry program package. *J. Chem. Phys.* **152**, 224108 (2020).

[12] Smith, D. G. *et al.* Psi4 1.4: open-source software for high-throughput quantum chemistry. *J. Chem. Phys.* **152**, 184108 (2020).

[13] Jeziorski, B., Moszynski, R. & Szalewicz, K. Perturbation theory approach to intermolecular potential energy surfaces of van der Waals complexes. *Chem. Rev.* **94**, 1887–1930 (1994).

[14] Ásgeirsson, V. *et al.* Nudged elastic band method for molecular reactions using energy-weighted springs combined with eigenvector following. *J. Chem. Theory Comput.* **17**, 4929–4945 (2021).

[15] Guo, Y. *et al.* Communication: an improved linear scaling perturbative triples correction for the domain based local pair-natural orbital based singles and doubles coupled cluster method [DLPNO-CCSD(T)]. *J. Chem. Phys.* **148**, 011101 (2018).

[16] Dunning Jr, T. H. Gaussian-basis sets for use in correlated molecular calculations. I. The atoms boron through neon and hydrogen. *J. Chem. Phys.* **90**, 1007–1023 (1989).

[17] Kendall, R. A., Dunning, T. H. & Harrison, R. J. Electron affinities of the first-row atoms revisited. Systematic basis sets and wave functions. *J. Chem. Phys.* **96**, 6796–6806 (1992).

[18] Saragi, R. T. *et al.* Switching hydrogen bonding to π-stacking: the thiophenol dimer and trimer. *J. Phys. Chem. Lett.* **12**, 1367–1373 (2021).

[19] Tikhonov, D. S. A simplistic computational procedure for tunneling splittings caused by proton transfer. *Structural Chemistry* **33**, 351–362 (2022).

[20] Li, W., Tikhonov, D. S. & Schnell, M. Double proton transfer across a table: the formic acid dimer-fluorobenzene complex. *Angew. Chem. Int. Ed.* **60**, 25674–25679 (2021).

[21] May, V. & Kühn, O. *Charge and Energy Transfer Dynamics in Molecular Systems* (John Wiley & Sons, 2023).

[22] Atkins, P. & Friedman, R. *Molecular Quantum Mechanics* (OUP Oxford, 2011).

[23] Kraitchman, J. Determination of molecular structure from microwave spectroscopic data. *Am. J. Phys.* **21**, 17–24 (1953).

[24] Lu, T. & Chen, Q. Independent gradient model based on Hirshfeld partition: A new method for visual study of interactions in chemical systems. *J. Comput. Chem.* **43**, 539–555 (2022).

[25] Lu, T. & Chen, F. Multiwfn: A multifunctional wavefunction analyzer. *J. Comput. Chem.* **33**, 580–592 (2012).

[26] Marquardt, R. & Quack, M. *Molecular Spectroscopy and Quantum Dynamics* (Elsevier, 2021).

[27] Sun, W. *et al.* Inducing transient enantiomeric excess in a molecular quantum racemic mixture with microwave fields. *Nat. Commun.* **14**, 934 (2023).

[28] Zhang, Y., Li, W., Luo, W., Zhu, Y. & Duan, C. High resolution jet-cooled infrared absorption spectra of $(HCOOH)_2$, $(HCOOD)_2$, and $HCOOH$-$HCOOD$ complexes in 7.2 μm region. *J. Chem. Phys.* **146**, 244306 (2017).

[29] Daly, A. M. *et al.* Microwave measurements of proton tunneling and structural parameters for the propiolic acid–formic acid dimer. *J. Chem. Phys.* **135**, 154304 (2011).

[30] Frisch, M. J. *et al.* Gaussian16 Revision A.03 (Gaussian Inc., Wallingford, CT, 2016).

[31] Richardson, J. O. & Althorpe, S. C. Ring-polymer instanton method for calculating tunneling splittings. *J. Chem. Phys.* **134**, 054109 (2011).

[32] Richardson, J. O. Ring-polymer instanton theory. *Int. Rev. Phys. Chem.* **37**, 171–216 (2018).

[33] Laude, G., Calderini, D., Tew, D. P. & Richardson, J. O. *Ab initio* instanton rate theory made efficient using Gaussian process regression. *Faraday Discuss.* **212**, 237–258 (2018).

[34] Fang, W., Zhu, Y.-C., Cheng, Y., Hao, Y.-P. & Richardson, J. O. Robust Gaussian process regression method for efficient tunneling pathway optimization: application to surface processes. *J. Chem. Theory Comput.* **20**, 3766–3778 (2024).

[35] Shavitt, I. & Bartlett, R. J. *Many-Body Methods in Chemistry and Physics: MBPT and Coupled-Cluster Theory* (Cambridge University Press, 2009).

[36] Valeev, E. F. Coupled-cluster methods with perturbative inclusion of explicitly correlated terms: a preliminary investigation. *Phys. Chem. Chem. Phys.* **10**, 106–113 (2008).

[37] Meisner, J. & Kästner, J. Dual-level approach to instanton theory. *J. Chem. Theory Comput.* **14**, 1865–1872 (2018).

[38] Laio, A. & Parrinello, M. Escaping free-energy minima. *Proc. Natl. Acad. Sci. U.S.A.* **99**, 12562–12566 (2002).

[39] Barducci, A., Bonomi, M. & Parrinello, M. Metadynamics. *WIREs Comput. Mol. Sci.* **1**, 826–843 (2011).

[40] Bussi, G. & Laio, A. Using metadynamics to explore complex free-energy landscapes. *Nat. Rev. Phys.* **2**, 200–212 (2020).

[41] Tikhonov, D. S. Metadynamics simulations with Bohmian-style bias potential. *J. Comput. Chem.* **44**, 1771–1775 (2023).

[42] Tikhonov, D. S. Pyramd scheme: A protocol for computing the infrared spectra of polyatomic molecules using ab initio molecular dynamics. *Spectroscopy Journal* **2**, 171–187 (2024). URL `https://www.mdpi.com/2813-446X/2/3/12`.

[43] Grimme, S., Brandenburg, J. G., Bannwarth, C. & Hansen, A. Consistent structures and interactions by density functional theory with small atomic orbital basis sets. *The Journal of Chemical Physics* **143**, 054107 (2015). URL `https://doi.org/10.1063/1.4927476`. `https://pubs.aip.org/aip/jcp/article-pdf/doi/10.1063/1.4927476/15497850/054107_1_online.pdf`.

[44] Berendsen, H. J. C., Postma, J. P. M., van Gunsteren, W. F., DiNola, A. & Haak, J. R. Molecular dynamics with coupling to an external bath. *J. Chem. Phys.* **81**, 3684–3690 (1984).
