# Supplementary material for: Environment Rearrangement Slows Down the Tunneling Rates of Proton Transfer in Sandwich-Like Molecular Clusters
Source: J Phys Chem Lett. 2026 Jul 30;17(32):9112–21. doi: 10.1021/acs.jpclett.6c01033 (PMC13488451; doi:10.1021/acs.jpclett.6c01033)
Supplement: Supplementary file 2 [file jz6c01033_si_001.zip › FiguresAndTables/Figure3/fa-pa-phf_metadynamics.pdf]

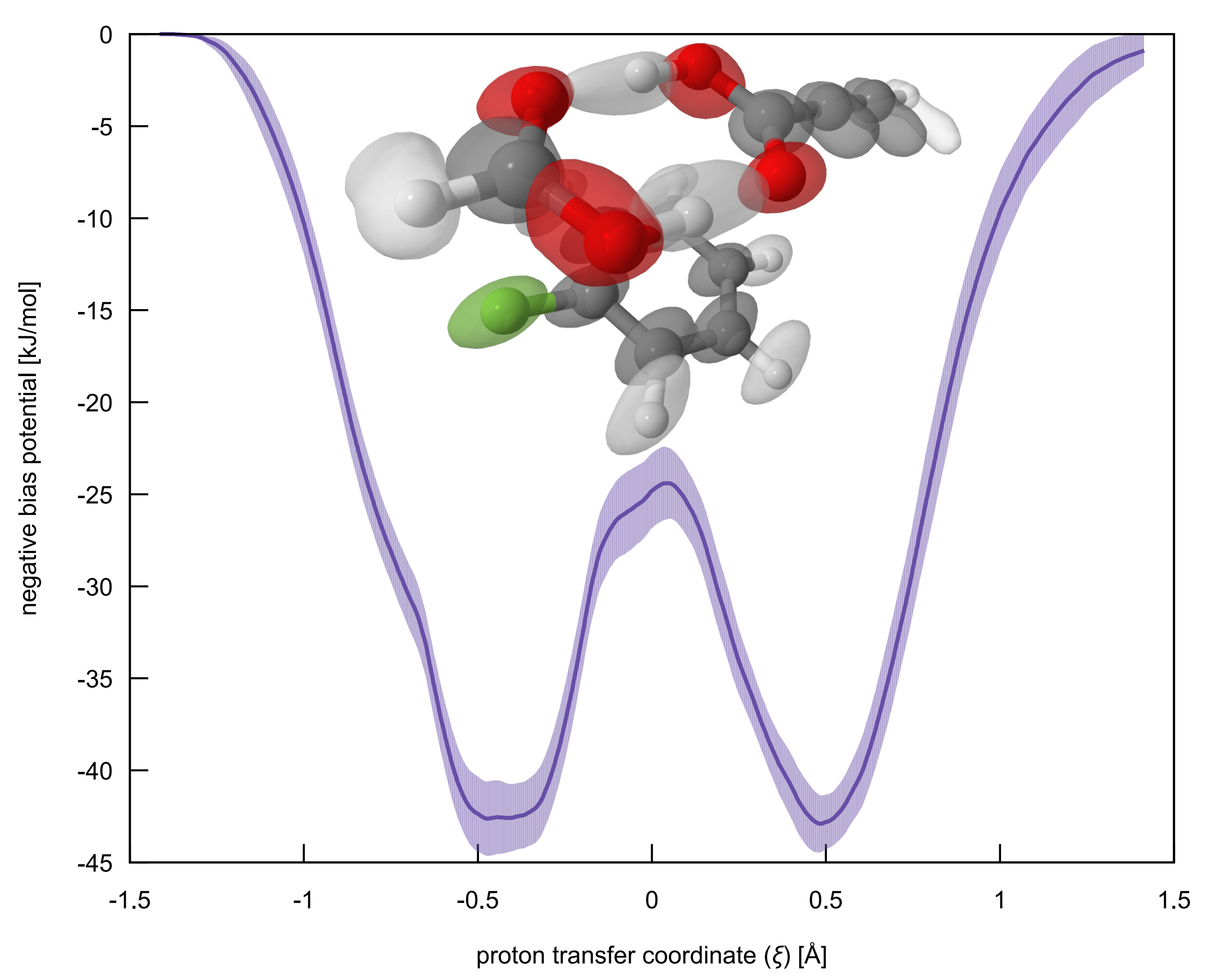

0
-5
-10
-15
-20
-25
-30
-35
-40
-45
negative bias potential [kJ/mol]
-1.5
-1
-0.5
0
0.5
1
1.5
proton transfer coordinate (ξ) [Å]
