# Supplementary material for: Environment Rearrangement Slows Down the Tunneling Rates of Proton Transfer in Sandwich-Like Molecular Clusters
Source: J Phys Chem Lett. 2026 Jul 30;17(32):9112–21. doi: 10.1021/acs.jpclett.6c01033 (PMC13488451; doi:10.1021/acs.jpclett.6c01033)
Supplement: Supplementary file 2 [file jz6c01033_si_001.zip › FiguresAndTables/Figure3/fa-x_potentials.pdf]

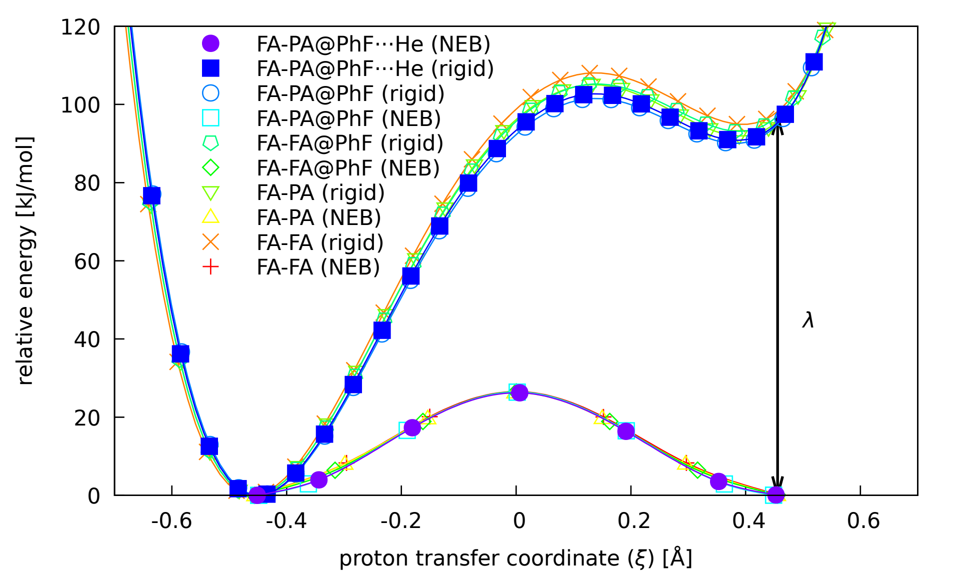

FA-PA@PhF···He (NEB)
FA-PA@PhF···He (rigid)
FA-PA@PhF (rigid)
FA-PA@PhF (NEB)
FA-FA@PhF (rigid)
FA-FA@PhF (NEB)
FA-PA (rigid)
FA-PA (NEB)
FA-FA (rigid)
FA-FA (NEB)
λ
relative energy [kJ/mol]
proton transfer coordinate (ξ) [Å]
0
20
40
60
80
100
120
-0.6
-0.4
-0.2
0
0.2
0.4
0.6
