# Supplementary material for: Environment Rearrangement Slows Down the Tunneling Rates of Proton Transfer in Sandwich-Like Molecular Clusters
Source: J Phys Chem Lett. 2026 Jul 30;17(32):9112–21. doi: 10.1021/acs.jpclett.6c01033 (PMC13488451; doi:10.1021/acs.jpclett.6c01033)
Supplement: Supplementary file 2 [file jz6c01033_si_001.zip › FiguresAndTables/IllustrativeModel/just_pes.pdf]

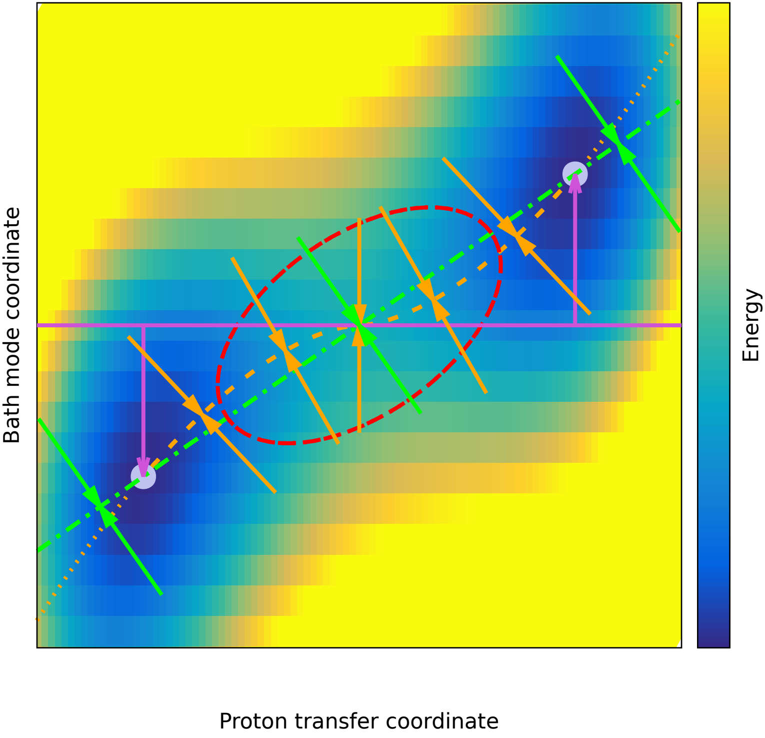
Bath mode coordinate
Proton transfer coordinate
Energy
